# Multi-shelled ECIF: improved extended connectivity interaction features for accurate binding affinity prediction

**DOI:** 10.1093/bioadv/vbad155

**Published:** 2023-10-20

**Authors:** Koji Shiota, Tatsuya Akutsu

**Affiliations:** Department of Intelligence Science and Technology, Graduate School of Informatics, Kyoto University, Kyoto, Kyoto 606-8501, Japan; Department of Intelligence Science and Technology, Graduate School of Informatics, Kyoto University, Kyoto, Kyoto 606-8501, Japan

## Abstract

**Motivation:**

Extended connectivity interaction features (ECIF) is a method developed to predict protein–ligand binding affinity, allowing for detailed atomic representation. It performed very well in terms of Comparative Assessment of Scoring Functions 2016 (CASF-2016) scoring power. However, ECIF has the limitation of not being able to adequately account for interatomic distances.

**Results:**

To investigate what kind of distance representation is effective for P-L binding affinity prediction, we have developed two algorithms that improved ECIF’s feature extraction method to take distance into account. One is multi-shelled ECIF, which takes into account the distance between atoms by dividing the distance between atoms into multiple layers. The other is weighted ECIF, which weights the importance of interactions according to the distance between atoms. A comparison of these two methods shows that multi-shelled ECIF outperforms weighted ECIF and the original ECIF, achieving a CASF-2016 scoring power Pearson correlation coefficient of 0.877.

**Availability and implementation:**

All the codes and data are available on GitHub (https://github.com/koji11235/MSECIFv2).

## 1 Introduction

Prediction of protein–ligand (P-L) binding affinity plays a very important role in virtual screening (VS). The docking-based VS is the large-scale application of the docking methodology. The components of the docking method are a search algorithm that generates poses within the binding site, scoring functions that quantify the quality of the docking poses, and one or more scoring functions to predict binding affinity. Examples of this scoring function include a classifier for active/inactive classes, a regressor for the absolute value of the binding free energy, and a compound ranking system that sorts compounds according to a certain score (Gilson and Zhou, 2007, Liu and Wang 2015, Pason and Sotriffer 2016, Maia *et al.* 2020, Kimber *et al.* 2021, Yang *et al.* 2022). For accurate and fast VS, various methods for affinity prediction have been developed, including physics-based (Abel *et al.* 2017, Khalak *et al.* 2021, King *et al.* 2021) and machine learning-based methods (Jimenez *et al.* 2018, Stepniewska-Dziubinska *et al.* 2018, Wojcikowski *et al.* 2019, Zheng *et al.* 2019, Jiang *et al.* 2021, Sanchez-Cruz *et al.* 2021, Moon *et al.* 2022, Zhang *et al.* 2023a,b).

Comparative Assessment of Scoring Functions 2016 (CASF-2016) (Li *et al.* 2014, Su *et al.* 2019) is a benchmark dataset created with the concept of evaluating scoring performance and docking performance separately. Four metrics are provided at CASF-2016: scoring power, which evaluates the linear correlation between predicted and experimental binding affinity values given a crystal structure; ranking power, which evaluates the accuracy of the binding affinity rank prediction for a given target protein; docking power, which evaluates the accuracy of the prediction of the native binding pose from 100 generated ligand configurations; and screening power, which predicts the binding ligand for a given protein. The performance of many machine learning methods developed in recent years has been evaluated by CASF-2016 and reported. Especially in terms of scoring power, methods using machine learning have been very successful, and their performance far exceeds that of physics-based methods. For example, the following have demonstrated very good performance in terms of CASF-2016 scoring power. PLEC-nn (Wojcikowski *et al.* 2019) is a model with fingerprint-based features trained on a neural network, and it has achieved a Pearson Correlation Coefficient (Pearson’s R) of 0.820. Kdeep (Jimenez *et al.* 2018) and Pafnucy (Stepniewska-Dziubinska *et al.* 2018) are convolutional neural networks (CNNs) trained on a 3D voxel representation of the P-L complex, with Pearson’s Rs of 0.82 and 0.78, respectively. InteractionGraphNet (IGN) (Jiang *et al.* 2021) represents the P-L complex as a graph and is trained by a graph neural network, with a Pearson’s R of 0.837. OnionNet (Zheng *et al.* 2019) is a multiple-layer inter-molecular contact-based feature that has been trained using a CNN, and it achieved a Pearson’s R of 0.816. Extended connectivity interaction features (ECIF) (Sanchez-Cruz *et al.* 2021) achieved a Pearson’s R of 0.866 in terms of CASF-2016 scoring power, the best performance reported to date. ECIF is unique in its ability to represent atoms in very fine detail. While many methods rely solely on elemental species to represent an atom, ECIF takes into account five additional factors in its representation: explicit valence, the number of attached heavy atoms, the number of attached hydrogens, aromaticity, and ring membership. The number of interactions between atoms represented by this method that exist within a certain threshold distance is defined as a feature value. A gradient boosting tree (GBT) was used for the model and trained with the features created above. For the training dataset, the PDBbind (Wang *et al.* 2004) v2016 plus a part of PDBbind v2019 was used. For more details, please refer to the original publication.

While feature extraction using the above method has the great advantage of being able to represent the atoms in detail, it has the problem of not being able to take into account any differences in distance within 6 Å. This is because the count value of how many interactions are within 6 Å is used as the feature value. For example, hydrogen bonds are generally reported to be 2.5–3.5 Å away and play a very important role in P-L interactions. The magnitude of the contribution to the P-L interaction is likely to be different for interactions at this distance and for more distant interactions. Therefore, we have attempted to improve the performance of ECIF by modifying it to take into account the interatomic distance. Despite the availability of multiple methods for quantifying distances, no systematic comparison has been conducted to determine the most effective approach. In this study, we prepared two methods for expressing distances that can be freely combined with methods of expressing atoms qualitatively. By comparing them, we seek to gain insight into the usefulness of various distance considerations.

The first method is to divide the distance into multiple shells. We call this method multi-shelled ECIF. With it, the distance can be taken into account by explicitly representing the count value region, such as 0–2.5 Å, 2.5–3.5 Å, 3.5–6.0 Å, etc. The second method is to apply weights that are the inverse of the square of the distance and make the sum of the weights the feature value. We call this method weighted ECIF. It was created based on the hypothesis that interactions at close distances are more important.

A comparison of multi-shelled ECIF, weighted ECIF, and ECIF shows that the multi-shell ECIF significantly outperformed the weighted ECIF and the original ECIF, achieving a scoring Pearson’s R of 0.877 for the CASF-2016. On the other hand, weighted ECIF was inferior to the original ECIF concerning CASF-2016, but outperformed the original ECIF on evaluation with the LIT-PCBA dataset. Both multi-shelled ECIF and weighted ECIF are freely available on GitHub (https://github.com/koji11235/MSECIFv2).

## 2 Methods

### 2.1 Feature extraction

All code used in this study was developed in Python 3.9.0. RDkit version 2022.09.5 was used. Both the multi-shelled ECIF and weighted ECIF used in this study were developed based on ECIF; see the original publication for more information on ECIF. An overview of ECIF is given below. ECIF represents each atom by six elements: an atom’s symbol, explicit valence, number of attached heavy atoms, number of attached hydrogens, aromaticity, and ring membership, and joined by a semicolon. For example, nitrogen is represented as N;3;2;1;0;0 if it has a valence of 3, two attached heavy atoms, one attached hydrogen, no aromaticity, and is not contained in a ring. The pairs of protein side atoms and ligand side atoms expressed in this way are then joined by hyphens. For example, the interaction between N;3;2;1;0;0 on the protein side and O;2;1;1;0;0 on the ligand side is expressed as N;3;2;1;0;0-O;2;1;1;0;0, which is the name of the feature. The count value of how many of the relevant interactions are present in the P-L complex within the distance threshold is then used as the feature value. The above process is then applied to all ECIF-type representation combinations to generate features of 1540 dimensions.

We found that the information on aromaticity in the original ECIF is almost the same as that of ring membership, and its contribution to the prediction is small. Therefore, we removed the aromaticity entry and represented each atom with five elements: an atom’s symbol, explicit valence, number of attached heavy atoms, number of attached hydrogens, and ring membership. For example, nitrogen is represented as N;3;2;1;0 if it has a valence of 3, two attached heavy atoms, one attached hydrogen, and is not contained in a ring. Both of the following two methods are based on this feature extraction method with modifications to allow distance to be accounted for.

Regarding the treatment of tautomers, each atom on the protein side is treated uniformly by dictionary-based mapping, and other tautomers are not considered (e.g. HIS-NE2 is treated only as N;3;2;1;1). For ligands, only the states described in the sdf file are considered and other tautomers are not considered. For the PDBbind dataset, only the states in the provided ligand sdf are considered, and for LIT-PCBA, only the states expressed by the SMILES described in the smi file are considered. The protonation/tautomeric state is an important element in accurately characterizing the details of biological systems. The dataset used in this study is based on standard protonation/tautomeric states using automated procedures. In particular, histidine residues are treated without a hydrogen atom bonded to the nitrogen. Furthermore, models deposited in the PDB often do not include curated flips of histidine, asparagine, and glutamine residues. This can lead to the phenomenon of protein–ligand complexes adopting non-optimal protonation states at the binding site and problems with hydrogen bond optimization not being taken into account.

#### 2.1.1 Multi-shelled ECIF

Multi-shelled ECIF is a subdivision of the feature counts of the original ECIF by dividing them into several distance regions. The feature name is defined by appending the upper limit distance of each region to the end of the original ECIF feature, together with a hyphen. The lower limit is identical to the upper limit of one previous feature but is not explicitly shown. For example, if we divide N;3;2;1;0-O;2;1;1;0 into three steps of 0–2.5 Å, 2.5–4.5 Å, and 4.5–6.0 Å, then N;3;2;1;0-O;2;1;1;0 is represented by dividing it into N;3;2;1;0-O;2;1;1;0–2.5, N;3;2;1;0-O;2;1;1;0–4.5, and N;3;2;1;0-O;2;1;1;0–6.0. The number of relevant interactions in each region is then used as the feature. For example, if there are three N;3;2;1;0-O;2;1;1;0 interactions within the distance threshold and the distances are 2, 3, and 4 Å, respectively, then the value of N;3;2;1;0-O;2;1;1;0–2.5 is 1, N;3;2;1;0-O;2;1;1;0–4.5 is 2, and the value of N;3;2;1;0-O;2;1;1;0–6.0 is 0.

#### 2.1.2 Weighted ECIF

Instead of treating interactions between atoms at any distance as one count, as in the original ECIF, the following feature extraction was performed to reflect the intuition that interactions at closer distances are more important. Weights were assigned as the inverse of the inter-atomic distance or the inverse of the square of the inter-atomic distance, and the weighted sum of the weights was used as the feature. The formulations are as follows.


(1) fX–Y=∑i∈PXj∈LYall(X,Y)pair1dij,

(2) fX–Y=∑i∈PXj∈LYall(X,Y)pair1dij2,

$d_{ij}$ denotes interatomic distance. *X* and *Y* indicate ECIF-style atom representations such as N;3;2;1;0;0. $P_{X}$ and $L_{Y}$ designate all X atoms on the protein side and all Y atoms on the ligand side, respectively. The procedure is as follows. Interaction is expressed in the similar way as ECIF, as in N;3;2;1;0-O;2;1;1;0. Atom pairs that exist at distances within a threshold are obtained. The weights above are calculated using $d_{ij}$ as the distance between atoms in each pair. The sum of the weights is calculated for each interaction and is defined as the feature value. For example, if three interactions of N;3;2;1;0-O;2;1;1;0 are within the distance threshold and the distances are 2, 3, and 4 Å, respectively, then the value of N;3;2;1;0-O;2;1;1;0 is 1/4 + 1/9 + 1/16 = 0.4236. The resulting feature has the same 1540 dimensions as the original ECIF.

#### 2.1.3 Ligand descriptor

To compare the performance with the original ECIF in CASF-2016, we used the same ligand descriptor as in the original work. A summary is given below. Of the 200 molecular descriptors available in the “Descriptors” module of RDkit (2020.03.1), those with zero variance, null values, and extreme values for the entire data set were removed. As a result, 170 molecular descriptors were used. All the ligand descriptors used are listed in the Supplementary Table S3. The 170-dimensional ligand descriptors generated were added to the multi-shelled ECIF and weighted ECIF features to train the model. The ligand descriptor for the LIT-PCBA dataset was calculated using RDkit (2022.09.5).

### 2.2 Training data

To compare the performance with the original ECIF, we used the same training data as in the original study. In brief, the “core set” (n = 285) from the PDBbind 2016 was used as the test set first. Then, what remained from the PDBbind 2016 “refined set” (*n* = 4,057) minus the “core set” was set as the primary source of training data. In addition to this, a “general set” of the PDBbind 2019 that meets the following criteria was added to the training data. (i) structures resolved by X-ray crystallography with a resolution better or equal than 3.0 Å, (ii) binding data reported accurately (not “>,” “<,” or “∼”) as inhibition constant (Ki) or dissociation constant (Kd) with a range from 1 pM to 10 mM, (iii) atom types of the ligand already included in the “refined set,” and (iv) structures not containing any protein–ligand atom pair at a distance of 2.0 Å or less. Three complexes (PDB ID: 2YLC, 3O7U, 3ZNR) with ligands containing atomic types that appear only once in the data set were discarded. In summary, the training set used in this study consists of 9299 P-L complexes, and the test set consists of 285 structures from CASF-2016. A list of all complexes included in the training and test sets is in the Supplementary Table S2. All protein structures were used without any other processing. On the other hand, Standardizer, JChem 22.6.0 was used for protonation and aromatization of the ligand.

### 2.3 Model

To compare the performance with the original ECIF, we also used GBT. It was implemented using the Scikit-learn Python library (1.0.1). All the models were trained to predict the binding affinity of the P-L complex denoted as pK, which is the logarithm of the negative base 10 of Ki or Kd. Before the hyperparameter optimization, the GBT model was built using the same hyperparameters described in ECIF (Sanchez-Cruz *et al.* 2021). The specific parameters are 20 000 boosting stages, a maximum depth of 8, a learning rate of 0.005, least squares regression as the loss function to optimize, 0.7 as the fraction of samples that fit individual learners, and “sqrt” as the fraction of features to look at for the optimal split. All remaining parameters were set to default. The best GBT hyperparameter for multi-shelled ECIF is as follows: 20 000 boosting stages, a maximum depth of 10, a learning rate of 0.005, least squares regression as the loss function to optimize, 0.6 as the fraction of samples that fit individual learners, min sample split of 3, and “sqrt” as the fraction of features to look at for the optimal split. The best GBT hyperparameter for weighted ECIF is as follows: 30 000 boosting stages, a maximum depth of 10, a learning rate of 0.005, least squares regression as the loss function to optimize, 0.6 as the fraction of samples that fit individual learners, min sample split of 2, and “sqrt” as the fraction of features to look at for the optimal split.

### 2.4 Cross-validation

The hyperparameters of the multi-shelled ECIF and weighted ECIF features and the GBT hyperparameters were adjusted by 10-fold cross-validation over the training set. We performed 10-fold cross-validation over the training set with 10 trials each with different random seeds in each condition. The average of the 10 trials was used to compare the conditions. In the cross-validation for the hyperparameters of the multi-shelled ECIF and weighted ECIF features, the GBT hyperparameters were fixed to the same hyperparameters described in ECIF (Sanchez-Cruz *et al.* 2021). The specific parameters are 20 000 boosting stages, a maximum depth of 8, a learning rate of 0.005, least squares regression as the loss function to optimize, 0.7 as the fraction of samples that fit individual learners, and “sqrt” as the fraction of features to look at for the optimal split. All remaining parameters were set to default. For computational convenience, the optimization of the GBT parameters was performed in two stages. In the first stage, n_estimators, learning_rate, and max_depth were examined, and in the second stage, min_sample_split, max_features, and subsample were examined. In the first stage, parameters other than n_estimators, learning_rate, and max_depth used values reported in ECIF. In the second phase, we fixed n_estimators, learning_rate, and max_depth, which were optimal in the first phase and examined the target parameters.

### 2.5 Evaluation method

We used CASF-2016 and LIT-PCBA dataset as independent test sets. For evaluation by CASF-2016, Model performance was evaluated using the Pearson’s R and the root mean square error (RMSE) of CASF-2016 scoring power. For evaluation by LIT-PCBA, all 15 targets of LIT-PCBA were predicted by the indicated model and evaluated with enrichment factor (EF) of 1%. For those that were given more than one protein template, evaluation was performed for all templates. Preparation of the protein pdb and ligand sdf for the LIT-PCBA dataset was done as follows. The smi file of ligand was loaded using RDkit (https://www.rdkit.org) to generate 3D conformation and add hydrogens. The files were then saved as sdf files. All template PDB files were protonated by MOE (ULC, 2023). Then, docking of ligand to protein was performed using GNINA (McNutt *et al.* 2021). If multiple templates were given in the target, docking was performed on all templates. The docked ligands were then saved as sdf files. Standardizer, JChem 22.6.0 was used for protonation and aromatization of the ligand. Additionally, CASF-2007, 2013, 2016, and 2019 defined by Orhobor *et al.* were evaluated using the training and test sets provided by them. Ligands were provided as mol files and used as is. Proteins were provided as mol2 files and were converted to pdb files using openbabel. It should be noted here that the training set differs between CASF-2016 as defined by Orhobor *et al.* and CASF-2016 by Sanchez-Cruz *et al.* mentioned above. The hyperparameters for the features and model used were those obtained in the 10-fold cross-validation described above.

### 2.6 Permutation feature importance

A feature importance analysis was conducted on the best model of multi-shelled ECIF. The feature importance was calculated by the permutation_importance function of the Scikit-learn Python library using 30 repeats with different random seeds.

## 3 Results

### 3.1 Results of multi-shelled ECIF

**Figure 1.**
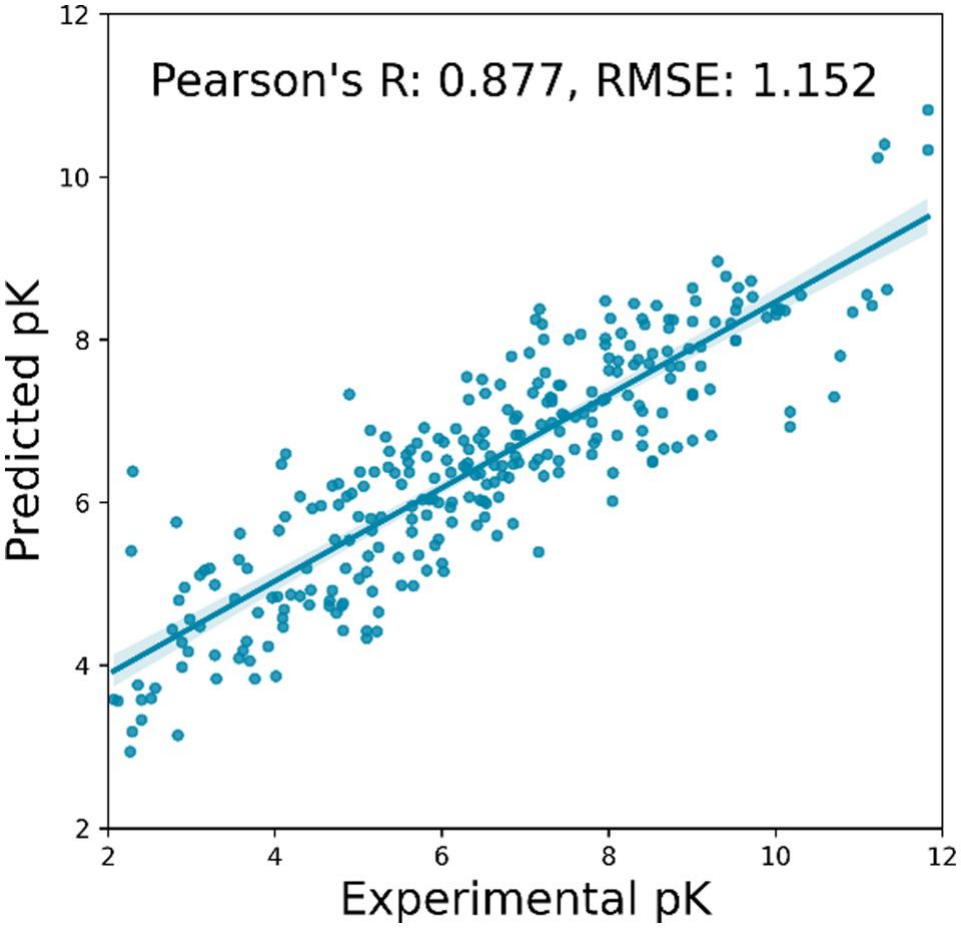
The best result of multi-shelled ECIF. Scatterplot of experimental values of CASF-2016 “core set” (*n* = 285) and predicted values by the best model of multi-shelled ECIF.

We first investigated the method of shell segmentation, which is a hyperparameter of multi-shelled ECIF features. Next, we examined the GBT hyperparameters. We then trained 5000 models with different random seeds using the best conditions obtained above and examined the distribution of CASF-2016 scoring power, Pearson’s R and RMSE. The best multi-shelled ECIF model achieved a Pearson’s R of 0.877 and an RMSE of 1.152 (Fig. 1). In the following, we describe the results of our examination of the hyperparameters of multi-shelled ECIF features and GBT hyperparameters, and finally compare multi-shelled ECIF with other methods.

### 3.2 Exploration of the multi-shelled ECIF feature parameter

Since the shell-splitting method potentially affects performance, we searched for the optimal shell-splitting method. To systematically study the approximate optimal shell-splitting method, we chose to split the shell with a constant step width. We performed a conditional study on the distance threshold and the step width. We checked the distribution of interatomic distances between ligand proteins and found that the shortest interatomic distance was about 2.0 Å and that interactions of 2.5 Å or less existed in only 0.03% of the total interactions existing within 6.0 Å. For this reason, we fixed the minimum shell at 2.5 Å for our study. To align the distance thresholds, the step width may be different for the terminal portion of each partitioning method than for the other portions. The conditions for the distance threshold and the step width were examined by 10-fold cross-validation over the training set. Ten-fold cross-validation was performed in each condition with 10 trials each with 10 different random seeds. A Bonferroni-corrected independent *t*-test was also used to check whether the differences between conditions were statistically significant. Initially, to determine the maximum distance at which interactions should be taken into consideration, we varied the distance threshold under conditions of 6–12 Å with a constant step width. Four different step widths were examined: 2.0, 1.5, 1.0, and 0.5 Å. Comparisons of distance thresholds within the same step width showed the maximum performance at a distance threshold of 10 Å for all conditions (Supplementary Fig. S1). A similar study was conducted for step width. With the distance threshold fixed, a comparison was made for different step widths of 0.5, 1.0, 1.5, and 2.0 Å. Comparisons of step widths showed the maximum performance at step width 2.0 Å for all conditions (Supplementary Fig. S2). Although there was no significant difference between 1.5 Å and 2.0 Å for almost all results, we decided to use 2.0 Å for step width because 2.0 Å showed higher performance under all conditions. Based on the above considerations, we decided to use 10.0 Å for the distance threshold and 2.0 Å for the step width. A heatmap listing all results is shown in Supplementary Fig. S3.

### 3.3 GBT parameters optimization for multi-shelled ECIF

The distance threshold and step width of multi-shelled ECIF were fixed at 10 Å and 2.0 Å, respectively, and the hyperparameter of GBT was optimized by 10-fold cross-validation over the training set. As well as exploration of the shell partitioning method of multi-shelled ECIF, 10-fold cross-validation was performed in each condition with 10 runs each with 10 different random seeds. Details are described in Section 2. The results are shown in Supplementary Fig. S4. The best GBT hyperparameter for multi-shelled ECIF is shown in Section 2.

### 3.4 Result of weighted ECIF

**Figure 2.**
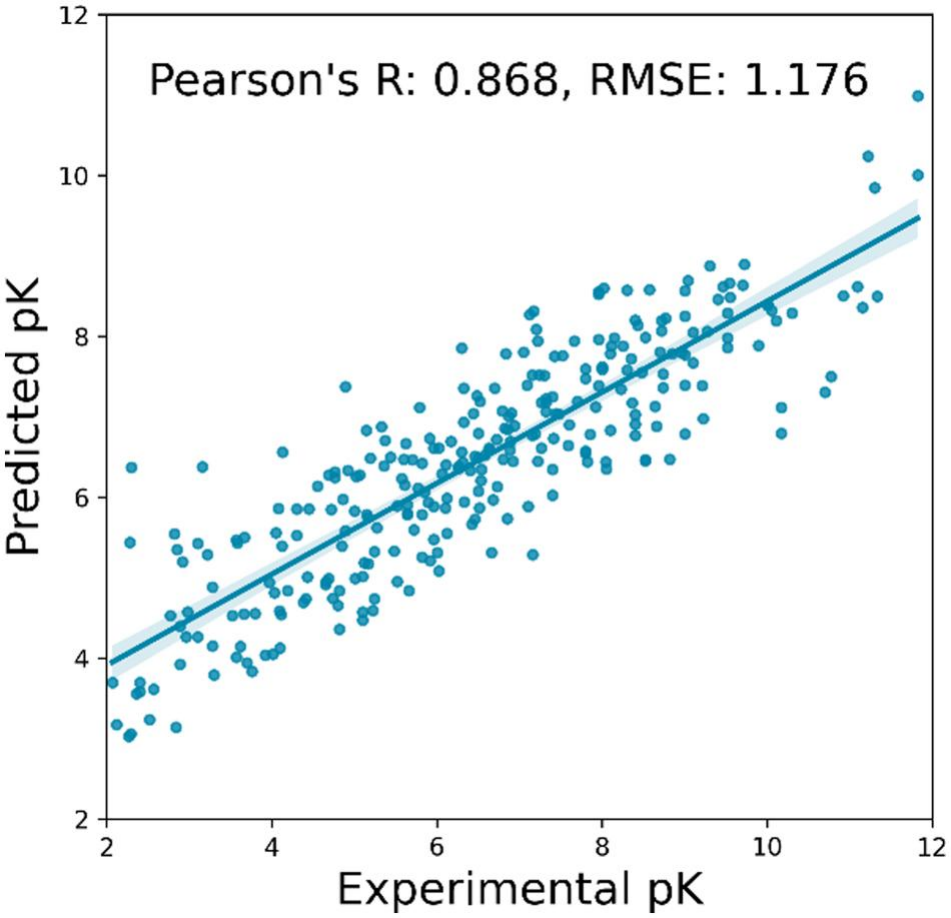
The best result of weighted ECIF. Scatterplot of experimental values of CASF-2016 “core set” (*n* = 285) and predicted values by the best model of weighted ECIF.

As well as multi-shelled ECIF, We first investigated the hyperparameter of weighted ECIF features. Next, we examined the GBT hyperparameters. We then trained 5000 models with different random seeds using the best conditions obtained above and examined the distribution of two metrics of CASF-2016 scoring power, Pearson’s R and RMSE. The best weighted ECIF model achieved a Pearson’s R of 0. 868 and an RMSE of 1.176 (Fig. 2). This is not as good as multi-shelled ECIF.

### 3.5 Exploration of the weighted ECIF feature parameter

Weighted ECIF has two hyperparameters: distance threshold and squared. The distance threshold is the threshold to which extent P-L interactions are considered, and “squared” is the choice of whether the weights are assigned as the inverse of the inter-atomic distance or the inverse of the square of the inter-atomic distance. As well as multi-shelled ECIF, the conditions for the distance threshold and “squared” were examined by 10-fold cross-validation over the training set. 10-fold cross-validation was performed in each condition with 10 trials each with 10 different random seeds. Bonferroni-corrected independent *t*-tests were also used to check whether the differences between conditions were statistically significant. First, the search for the optimal distance threshold was conducted with “squared” fixed. To investigate the optimal distance threshold, we examined it under the conditions of 4.0–12.0 Å. When “squared” is false, the best performance is obtained when the distance threshold is 8.0 Å, and when “squared” is true, the best performance is obtained when the distance threshold is 10.0 Å (Supplementary Fig. S5). Next, we checked whether squared was True or False for the same distance threshold to see which performed better. The performance was significantly better when “squared” was True for all distance thresholds >8 Å (Supplementary Fig. S6). As a result, the distance threshold 10 Å, squared True had the best performance, thus these values were used. The results are shown in Supplementary Fig. S7.

### 3.6 GBT parameters optimization for weighted ECIF

The distance threshold and “squared” of weighted ECIF were fixed at 10 Å and True respectively, and the hyperparameter of GBT was optimized by 10-fold cross-validation over the training set. As well as the exploration of GBT parameters for multi-shelled ECIF, 10-fold cross-validation was performed in each condition with 10 trials each with 10 different random seeds. The results are shown in Supplementary Fig. S8. The best GBT hyperparameter for weighted ECIF is shown in the method.

### 3.7 Comparing performance through statistical testing

**Figure 3.**
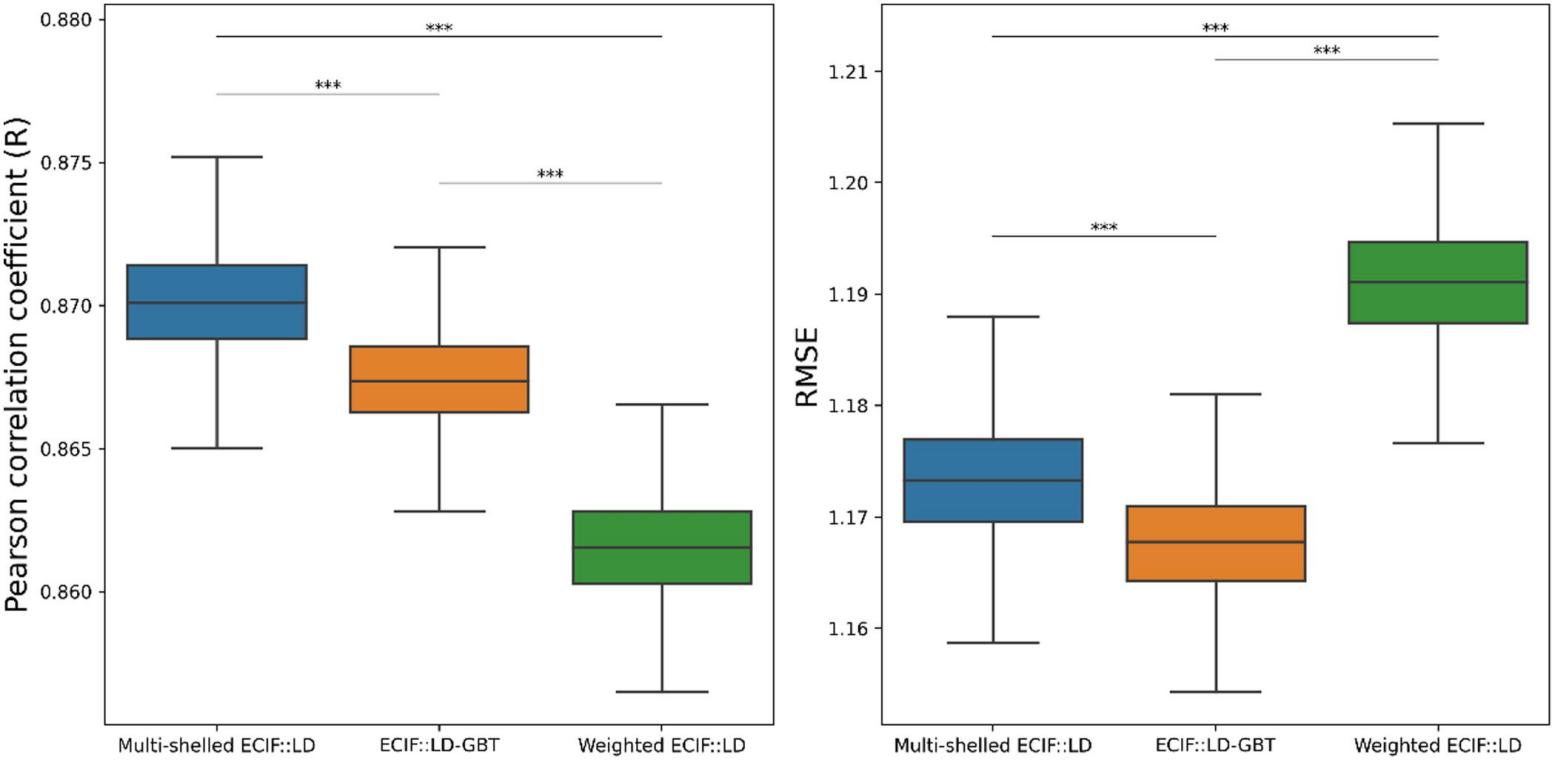
Comparing performance through statistical testing. Each boxplot represents the results of 5000 models trained with different random seeds and evaluated with CASF-2016 “core set” Pearson’s R (left) and RMSE (right). Combinations that are statistically significant by Bonferroni-corrected independent *t*-test are marked with *. (***P < 0.001, **0.001 ≤ *P* < 0.01, *0.01 ≤ *P* < 0.05).

To compare the quality of features rather than the performance of the best models, we trained 5000 models with different random seeds using the best parameters obtained above and compared their CASF-2016 Pearson’s R and RMSE distributions for multi-shelled ECIF, weighted ECIF, and original ECIF. Then, a Bonferroni-corrected independent *t*-test was used to check whether the difference was statistically significant. The results showed that multi-shelled ECIF performed significantly better than the original ECIF and weighted ECIF in Pearson’s R (Fig. 3). The t-statistic is 75.53, and the *P*-value is smaller than the smallest value that can be represented in Python (∼2.2e−308). The effect size for this difference was very large, with a Cohen’s d of 1.51. On the other hand, the original ECIF was significantly lower than multi-shelled ECIF for RMSE. The Cohen’s d was 1.08, indicating a large effect. In VS, ranking binding affinities is more important than accurately predicting binding affinity values. Therefore, multi-shelled ECIF with higher Pearson’s R is more useful in VS. In addition to the difference between the averages of the 5000 models, the performance of the best models of each of the multi-shelled ECIF and ECIF was compared by Mann–Whitney *U*-test at 10 000 bootstrapped Pearson’s R and RMSE. The Pearson’s R of the best multi-shelled ECIF model is 0.877 and the RMSE is 1.152. The Pearson’s R of the best ECIF model is 0.874 and the RMSE is 1.151. The result showed that the best multi-shelled ECIF model was significantly higher than the best ECIF model for Pearson’s R (*P*-value = 4.9e−56). The effect size, as measured by Cliff’s delta, was 0.128, indicating a small effect. On the other hand, no significant difference was found for RMSE (*P*-value = 0.147). The Cliff’s delta was 0.00856, indicating that there was almost no difference between the groups.

### 3.8 Comparison with other reported scoring functions

**Figure 4.**
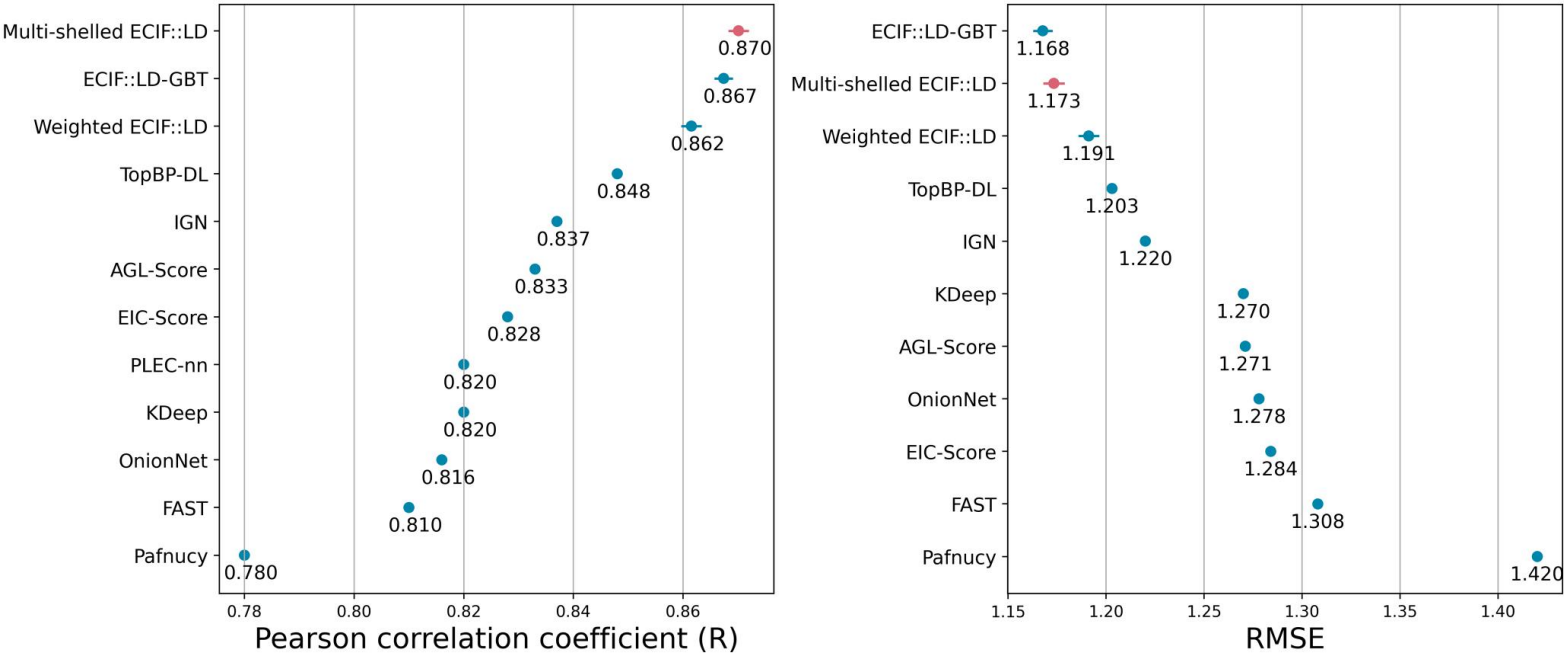
Comparison with other reported scoring functions. Comparison of reported evaluation results by CASF-2016 Pearson’s R (left panel) and RMSE (right panel). Multi-shell ECIF results are highlighted in pink. For multi-shelled ECIF, weighted ECIF, and ECIF, the mean of 5000 models is displayed and the standard deviation is indicated by error bars (Cang *et al.* 2018, Jiang *et al.* 2021, Jimenez *et al.* 2018, Jones *et al.* 2021, Nguyen and Wei 2019a, 2019b, Sanchez-Cruz *et al.* 2021, Stepniewska-Dziubinska *et al.* 2018, Wojcikowski *et al.* 2019, Zheng *et al.* 2019).

A comparison of our results with the evaluation results by CASF-2016 of previously reported methods is shown in Fig. 4. Multi-shelled ECIF achieved the best performance in terms of average Pearson’s R for CASF-2016 scoring power among the methods reported to date. The results show that the distance consideration improves the performance of ECIF.

### 3.9 Evaluation by other CASF dataset

A previous report on a modification to consider distance in ECIF was made by Orhobor *et al.* (2022). They defined four datasets, CASF-2007, CASF-2013, CASF-2016, and CASF-2019, for a comprehensive comparison between ECIF and their developed method, pair distance ECIF (PDECIF). It should be noted here that the training set differs between CASF-2016 as defined by Orhobor *et al.* and CASF-2016 by Sanchez-Cruz *et al.* mentioned above. As PDECIF is a similar method to our multi-shelled ECIF, we also evaluated multi-shelled ECIF on the four CASF datasets used by Orhobor *et al.* and compared their results. The results are presented in Supplementary Table S4; as Orhobor *et al.* reported all the results for PDECIF and ECIF for several distance hyperparameters, only the best ones for each dataset are extracted and cited. For those without ligand descriptors, multi-shelled ECIF showed the best Pearson’s R for all four datasets. For those containing Ligand descriptors, multi-shelled ECIF::LD showed the best results, except for CASF-2013, where PDECIF::LD showed the best results. While Orhobor adjusted GBT hyperparameters using CV for each dataset, we achieved great results with the above parameters without individual adjustments.

### 3.10 Evaluation by LIT-PCBA dataset

**Figure 5.**
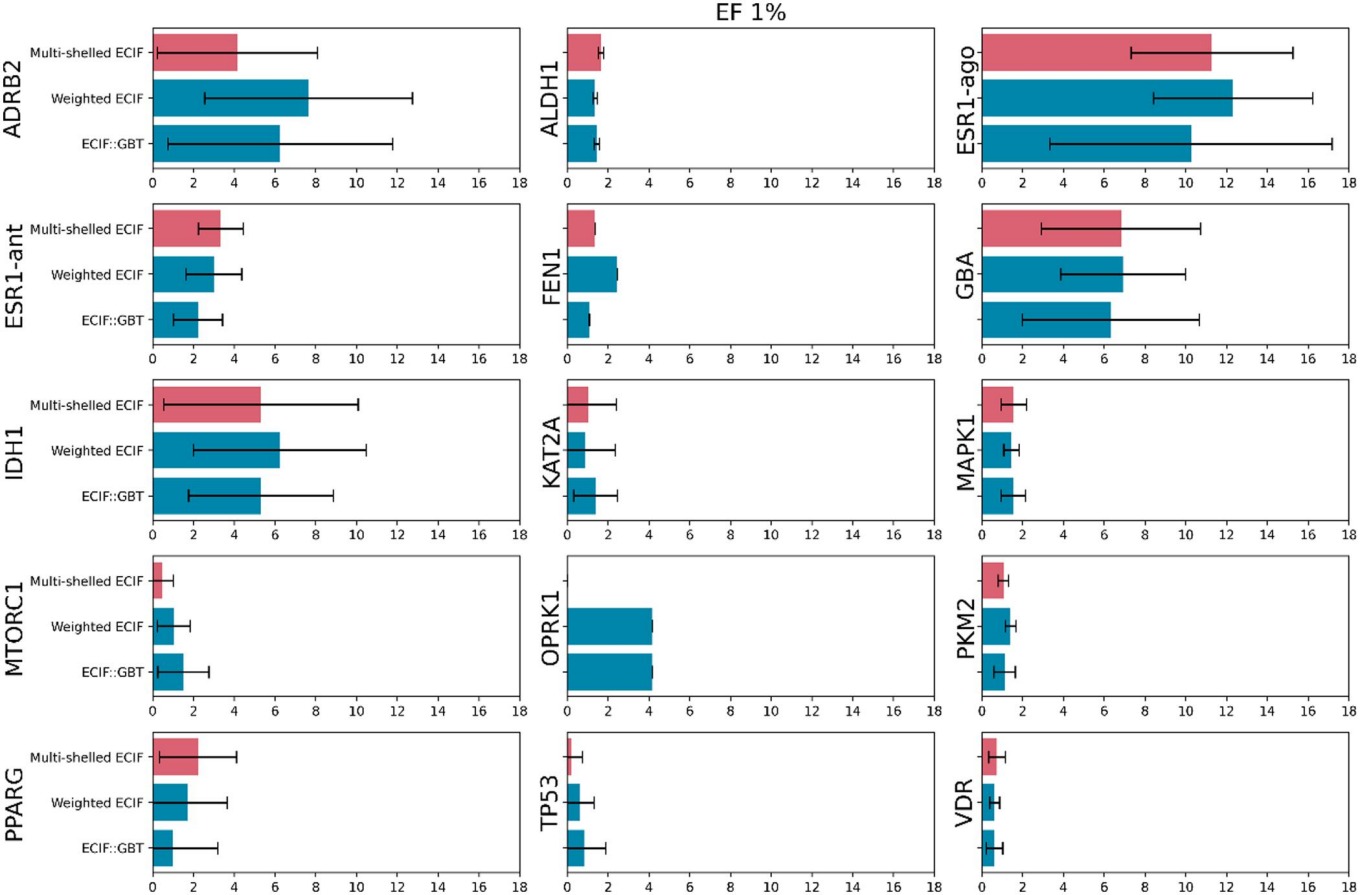
Result of the evaluation by LIT-PCBA dataset. The bars indicate the mean value of the Enrichment Factor 1% (EF1%) and the error bars indicate the standard deviation (SD). If only one template is provided for a given target (i.e. FEN1), the width of the error bar is 0.

Validation was performed on the LIT-PCBA dataset to confirm that multi-shelled ECIF’s performance is not due to a bias present in CASF-2016, but rather that it performs well against other datasets. Predictions were made using the best models of multi-shelled ECIF, weighted ECIF, and ECIF respectively for the 15 targets of LIT-PCBA and evaluated with EF of 1%. As a result, in 9 of 15 targets, multi-shelled ECIF performed as well or better than ECIF in terms of mean EF1% (Fig. 5). Results from CASF-2016 and LIT-PCBA show that multi-shelled ECIF, modified to account for interatomic distances, outperforms ECIF in VS. On the other hand, weighted ECIF outperformed original ECIF in 11 out of 15 LIT-PCBA targets in terms of mean EF1%, even though weighted ECIF was inferior to original ECIF for CASF-2016. Depending on the data set, weighted ECIF may be a worthwhile choice.

### 3.11 Feature importance

**Figure 6.**
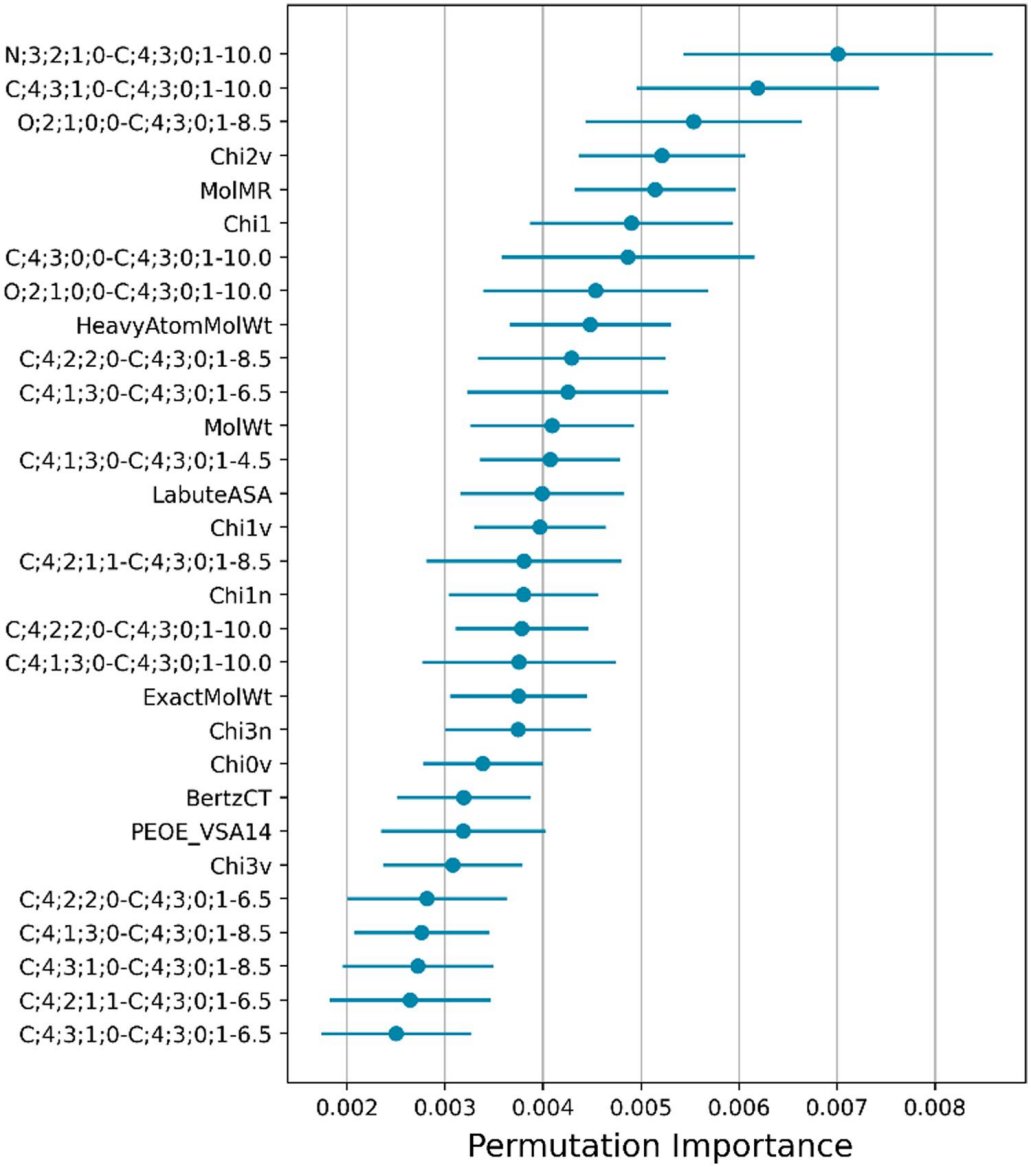
Feature importance of the best multi-shelled ECIF model. The top 30 permutation feature importance of the best model of multi-shelled ECIF is shown. All non-multi-shelled ECIF descriptors are ligand descriptors. For more details about each ligand descriptor, please refer to RDkit (https://www.rdkit.org/).

To investigate the performance improvement of adding our features to the ligand descriptors, we compared the performance of our model with that of a model trained with only ligand descriptors as features (Supplementary Fig. S9). Ten models trained with only ligand descriptors were evaluated in CASF-2016, with an average Pearson’s R of about 0.76, which is 0.1 lower than when multi-shell ECIF or weighted ECIF features were added (about 0.87). The results show that there is a significant performance improvement by adding our features. We conducted a feature importance analysis on the best model of multi-shelled ECIF. The top 30 features with the highest feature importance are shown in Fig. 6. Multi-shelled ECIF features appear at the top of the list, indicating their substantial contribution to prediction. Of the 71 features in the top 1% of the total 7,100 features, 43 features were from multi-shelled ECIF. For the ligand side, those containing C;4;3;0;1, which are derived from aromatic rings, are in the top positions. For the protein side, N;3;2;1;0, O;2;1;0;0, and C;4;3;1;0, were found in the top positions. As for N;3;2;1;0;0 and O;2;1;0;0, they are derived from the peptide bond, while C;4;3;1;0, is derived from the alpha carbon. This finding suggests that the interaction between these atoms on the protein side and the aromatic ring of the ligand is crucial. Contrary to our intuition, the highest levels of permutation feature importance included many interactions at relatively distant distances of 10 Å and 8.5 Å. Features at 2–4 Å were predicted to be important, where hydrogen bonding and ionic bonding are dominant, but only 3 of the 71 features in the top 1% contained features of this range of distances. Therefore, to compare the importance of features by distance, we split the multi-shelled ECIF features by distance and compared the performance of models trained only with features at specific distances. Ligand descriptors were not used to simply compare the importance of the features only. We trained 10 models each with different random seeds in each condition and compared them with the average CASF-2016 Pearson’s R and RMSE. As a result, the model trained with only 4.5–6.5 Å features had the best performance (Supplementary Fig. S10). The results show that the features at distances 4.5–6.5 Å have the highest contribution to the prediction. Considering the permutation feature importance results, it seems that the 4.5–6.5 Å distance features play a major role in the prediction, with some particularly important interactions above 6.5 Å (such as the hydrophobic interaction with alpha carbon) making the prediction more accurate.

## 4 Conclusions

We have made several modifications to ECIF to allow it to account for distance. One is multi-shelled ECIF, in which several virtual shells are created by dividing the inter-atomic distance into several regions, and the count values of interactions in each shell are used as the feature. The other is weighted ECIF, in which the features are weighted sums of the squared inverse of the interatomic distances. The above two methods and the original ECIF were compared in terms of CASF-2016 scoring power. The results showed no improvement from the original ECIF for weighted ECIF, but significant improvement for multi-shelled ECIF. This indicates that the multi-shelled type is more effective in considering interatomic distances. For multi-shelled ECIF, a Pearson’s R of 0.877 and RMSE of 1.152 were achieved in terms of CASF-2016 scoring power. Weighted ECIF was not as good as original ECIF in CASF-2016 but was superior to the original ECIF in the evaluation on the LIT-PCBA dataset. Multi-shelled ECIF is a method that can describe P-L interactions more precisely as the distance threshold is set farther away and as the step width is set smaller. As more experimental data become available in the future, it is expected that multi-shelled ECIF will be trainable using thresholds at farther distances and smaller step widths, thus further improving its performance. Our method is highly dependent on the number of hydrogen atoms and explicit valences. Therefore, the present method using an automatic procedure and standard protonation/tautomeric states has the limitations described in the Feature extraction section. Therefore, the preparation of protein–ligand complexes that take into account the optimized structure of the hydrogen bonding network at the desired pH may improve the accuracy of the model.

## Supplementary Material

vbad155_Supplementary_Data

## Data Availability

All the codes and data are available on GitHub (https://github.com/koji11235/MSECIFv2).
