# Supplementary material for: Multi-shelled ECIF: improved extended connectivity interaction features for accurate binding affinity prediction
Source: Bioinform Adv. 2023 Oct 20;3(1):vbad155. doi: 10.1093/bioadv/vbad155 (PMC10625475; doi:10.1093/bioadv/vbad155)
Supplement: vbad155_Supplementary_Data [file vbad155_supplementary_data.docx]

**Supplementary data for "Multi-shelled ECIF: Improved extended connectivity interaction features for accurate binding affinity prediction."**

Koji Shiota, Tatsuya Akutsu

Department of Intelligence Science and Technology, Graduate School of Informatics, Kyoto University, Kyoto, Kyoto 606-8501, Japan

Bioinformatics Center, Institute for Chemical Research, Kyoto University, Uji, Kyoto 611-0011, Japan

Table of contents

[Table S1. Correspondence between protein side atoms in PDB and ECIF atom type. 2](#_Toc137157707)

[Table S2. PDB IDs used for training set and validation set. 4](#_Toc137157708)

[Table S3. RDkit ligand descriptors used for training along with multi-shelled ECIF and weighted ECIF features. 11](#_Toc137157709)

[Supplementary Figure 3. Result of the multi-shelled ECIF feature parameter exploration. 12](#_Toc137157710)

[Supplementary Figure 4. Result of the GBT parameters optimization of the multi-shelled ECIF. 13](#_Toc137157711)

[Supplementary Figure 7. Result of the weighted ECIF feature parameter exploration. 14](#_Toc137157712)

[Supplementary Figure 8. Result of the GBT parameters optimization of the weighted ECIF. 15](#_Toc137157713)

[Supplementary Figure 9. Comparison with the models trained by only ligand descriptors. 16](#_Toc137157714)

[Supplementary Figure 10. Comparison with the models trained by the features at specific distance range. 17](#_Toc137157715)

# Table S1. Correspondence between protein side atoms in PDB and ECIF atom type.

| PDB residue | PDB atom | ECIF atom type |
| --- | --- | --- |
| ALA | ALA-C | C;4;3;0;0 |
| ALA | ALA-CA | C;4;3;1;0 |
| ALA | ALA-CB | C;4;1;3;0 |
| ALA | ALA-N | N;3;2;1;0 |
| ALA | ALA-O | O;2;1;0;0 |
| ALA | ALA-OXT | O;2;1;0;0 |
| ARG | ARG-C | C;4;3;0;0 |
| ARG | ARG-CA | C;4;3;1;0 |
| ARG | ARG-CB | C;4;2;2;0 |
| ARG | ARG-CD | C;4;2;2;0 |
| ARG | ARG-CG | C;4;2;2;0 |
| ARG | ARG-CZ | C;6;3;0;0 |
| ARG | ARG-N | N;3;2;1;0 |
| ARG | ARG-NE | N;4;2;1;0 |
| ARG | ARG-NH1 | N;4;1;2;0 |
| ARG | ARG-NH2 | N;4;1;2;0 |
| ARG | ARG-O | O;2;1;0;0 |
| ARG | ARG-OXT | O;2;1;0;0 |
| ASN | ASN-C | C;4;3;0;0 |
| ASN | ASN-CA | C;4;3;1;0 |
| ASN | ASN-CB | C;4;2;2;0 |
| ASN | ASN-CG | C;4;3;0;0 |
| ASN | ASN-N | N;3;2;1;0 |
| ASN | ASN-ND2 | N;3;1;2;0 |
| ASN | ASN-O | O;2;1;0;0 |
| ASN | ASN-OD1 | O;2;1;0;0 |
| ASN | ASN-OXT | O;2;1;0;0 |
| ASP | ASP-C | C;4;3;0;0 |
| ASP | ASP-CA | C;4;3;1;0 |
| ASP | ASP-CB | C;4;2;2;0 |
| ASP | ASP-CG | C;5;3;0;0 |
| ASP | ASP-N | N;3;2;1;0 |
| ASP | ASP-O | O;2;1;0;0 |
| ASP | ASP-OD1 | O;2;1;0;0 |
| ASP | ASP-OD2 | O;2;1;0;0 |
| ASP | ASP-OXT | O;2;1;0;0 |
| CYS | CYS-C | C;4;3;0;0 |
| CYS | CYS-CA | C;4;3;1;0 |
| CYS | CYS-CB | C;4;2;2;0 |
| CYS | CYS-N | N;3;2;1;0 |
| CYS | CYS-O | O;2;1;0;0 |
| CYS | CYS-OXT | O;2;1;0;0 |
| CYS | CYS-SG | S;2;1;1;0 |
| GLN | GLN-C | C;4;3;0;0 |
| GLN | GLN-CA | C;4;3;1;0 |
| GLN | GLN-CB | C;4;2;2;0 |
| GLN | GLN-CD | C;4;3;0;0 |
| GLN | GLN-CG | C;4;2;2;0 |
| GLN | GLN-N | N;3;2;1;0 |
| GLN | GLN-NE2 | N;3;1;2;0 |
| GLN | GLN-O | O;2;1;0;0 |
| GLN | GLN-OE1 | O;2;1;0;0 |
| GLN | GLN-OXT | O;2;1;0;0 |
| GLU | GLU-C | C;4;3;0;0 |
| GLU | GLU-CA | C;4;3;1;0 |
| GLU | GLU-CB | C;4;2;2;0 |
| GLU | GLU-CD | C;5;3;0;0 |
| GLU | GLU-CG | C;4;2;2;0 |
| GLU | GLU-N | N;3;2;1;0 |
| GLU | GLU-O | O;2;1;0;0 |
| GLU | GLU-OE1 | O;2;1;0;0 |
| GLU | GLU-OE2 | O;2;1;0;0 |
| GLU | GLU-OXT | O;2;1;0;0 |
| GLY | GLY-C | C;4;3;0;0 |
| GLY | GLY-CA | C;4;2;2;0 |
| GLY | GLY-N | N;3;2;1;0 |
| GLY | GLY-O | O;2;1;0;0 |
| GLY | GLY-OXT | O;2;1;0;0 |
| HIS | HIS-C | C;4;3;0;0 |
| HIS | HIS-CA | C;4;3;1;0 |
| HIS | HIS-CB | C;4;2;2;0 |
| HIS | HIS-CD2 | C;4;2;1;1 |
| HIS | HIS-CE1 | C;4;2;1;1 |
| HIS | HIS-CG | C;4;3;0;1 |
| HIS | HIS-N | N;3;2;1;0 |
| HIS | HIS-ND1 | N;3;2;0;1 |
| HIS | HIS-NE2 | N;3;2;1;1 |
| HIS | HIS-O | O;2;1;0;0 |
| HIS | HIS-OXT | O;2;1;0;0 |
| ILE | ILE-C | C;4;3;0;0 |
| ILE | ILE-CA | C;4;3;1;0 |
| ILE | ILE-CB | C;4;3;1;0 |
| ILE | ILE-CD1 | C;4;1;3;0 |
| ILE | ILE-CG1 | C;4;2;2;0 |
| ILE | ILE-CG2 | C;4;1;3;0 |
| ILE | ILE-N | N;3;2;1;0 |
| ILE | ILE-O | O;2;1;0;0 |
| ILE | ILE-OXT | O;2;1;0;0 |
| LEU | LEU-C | C;4;3;0;0 |
| LEU | LEU-CA | C;4;3;1;0 |
| LEU | LEU-CB | C;4;2;2;0 |
| LEU | LEU-CD1 | C;4;1;3;0 |
| LEU | LEU-CD2 | C;4;1;3;0 |
| LEU | LEU-CG | C;4;3;1;0 |
| LEU | LEU-N | N;3;2;1;0 |
| LEU | LEU-O | O;2;1;0;0 |
| LEU | LEU-OXT | O;2;1;0;0 |
| LYS | LYS-C | C;4;3;0;0 |
| LYS | LYS-CA | C;4;3;1;0 |
| LYS | LYS-CB | C;4;2;2;0 |
| LYS | LYS-CD | C;4;2;2;0 |
| LYS | LYS-CE | C;4;2;2;0 |
| LYS | LYS-CG | C;4;2;2;0 |
| LYS | LYS-N | N;3;2;1;0 |
| LYS | LYS-NZ | N;4;1;3;0 |
| LYS | LYS-O | O;2;1;0;0 |
| LYS | LYS-OXT | O;2;1;0;0 |
| MET | MET-C | C;4;3;0;0 |
| MET | MET-CA | C;4;3;1;0 |
| MET | MET-CB | C;4;2;2;0 |
| MET | MET-CE | C;4;1;3;0 |
| MET | MET-CG | C;4;2;2;0 |
| MET | MET-N | N;3;2;1;0 |
| MET | MET-O | O;2;1;0;0 |
| MET | MET-OXT | O;2;1;0;0 |
| MET | MET-SD | S;2;2;0;0 |
| PHE | PHE-C | C;4;3;0;0 |
| PHE | PHE-CA | C;4;3;1;0 |
| PHE | PHE-CB | C;4;2;2;0 |
| PHE | PHE-CD1 | C;4;2;1;1 |
| PHE | PHE-CD2 | C;4;2;1;1 |
| PHE | PHE-CE1 | C;4;2;1;1 |
| PHE | PHE-CE2 | C;4;2;1;1 |
| PHE | PHE-CG | C;4;3;0;1 |
| PHE | PHE-CZ | C;4;2;1;1 |
| PHE | PHE-N | N;3;2;1;0 |
| PHE | PHE-O | O;2;1;0;0 |
| PHE | PHE-OXT | O;2;1;0;0 |
| PRO | PRO-C | C;4;3;0;0 |
| PRO | PRO-CA | C;4;3;1;1 |
| PRO | PRO-CB | C;4;2;2;1 |
| PRO | PRO-CD | C;4;2;2;1 |
| PRO | PRO-CG | C;4;2;2;1 |
| PRO | PRO-N | N;3;3;0;1 |
| PRO | PRO-O | O;2;1;0;0 |
| PRO | PRO-OXT | O;2;1;0;0 |
| SER | SER-C | C;4;3;0;0 |
| SER | SER-CA | C;4;3;1;0 |
| SER | SER-CB | C;4;2;2;0 |
| SER | SER-N | N;3;2;1;0 |
| SER | SER-O | O;2;1;0;0 |
| SER | SER-OG | O;2;1;1;0 |
| SER | SER-OXT | O;2;1;0;0 |
| THR | THR-C | C;4;3;0;0 |
| THR | THR-CA | C;4;3;1;0 |
| THR | THR-CB | C;4;3;1;0 |
| THR | THR-CG2 | C;4;1;3;0 |
| THR | THR-N | N;3;2;1;0 |
| THR | THR-O | O;2;1;0;0 |
| THR | THR-OG1 | O;2;1;1;0 |
| THR | THR-OXT | O;2;1;0;0 |
| TRP | TRP-C | C;4;3;0;0 |
| TRP | TRP-CA | C;4;3;1;0 |
| TRP | TRP-CB | C;4;2;2;0 |
| TRP | TRP-CD1 | C;4;2;1;1 |
| TRP | TRP-CD2 | C;4;3;0;1 |
| TRP | TRP-CE2 | C;4;3;0;1 |
| TRP | TRP-CE3 | C;4;2;1;1 |
| TRP | TRP-CG | C;4;3;0;1 |
| TRP | TRP-CH2 | C;4;2;1;1 |
| TRP | TRP-CZ2 | C;4;2;1;1 |
| TRP | TRP-CZ3 | C;4;2;1;1 |
| TRP | TRP-N | N;3;2;1;0 |
| TRP | TRP-NE1 | N;3;2;1;1 |
| TRP | TRP-O | O;2;1;0;0 |
| TRP | TRP-OXT | O;2;1;0;0 |
| TYR | TYR-C | C;4;3;0;0 |
| TYR | TYR-CA | C;4;3;1;0 |
| TYR | TYR-CB | C;4;2;2;0 |
| TYR | TYR-CD1 | C;4;2;1;1 |
| TYR | TYR-CD2 | C;4;2;1;1 |
| TYR | TYR-CE1 | C;4;2;1;1 |
| TYR | TYR-CE2 | C;4;2;1;1 |
| TYR | TYR-CG | C;4;3;0;1 |
| TYR | TYR-CZ | C;4;3;0;1 |
| TYR | TYR-N | N;3;2;1;0 |
| TYR | TYR-O | O;2;1;0;0 |
| TYR | TYR-OH | O;2;1;1;0 |
| TYR | TYR-OXT | O;2;1;0;0 |
| VAL | VAL-C | C;4;3;0;0 |
| VAL | VAL-CA | C;4;3;1;0 |
| VAL | VAL-CB | C;4;3;1;0 |
| VAL | VAL-CG1 | C;4;1;3;0 |
| VAL | VAL-CG2 | C;4;1;3;0 |
| VAL | VAL-N | N;3;2;1;0 |
| VAL | VAL-O | O;2;1;0;0 |
| VAL | VAL-OXT | O;2;1;0;0 |

# Table S2. PDB IDs used for training set and validation set.

Train set

| 10gs, 11gs, 13gs, 16pk, 184l, 185l, 186l, 187l, 188l, 1a07, 1a08, 1a0q, 1a1b, 1a1c, 1a1e, 1a28, 1a3e, 1a42, 1a4h, 1a4k, 1a4r, 1a4w, 1a50, 1a52, 1a5h, 1a69, 1a8i, 1a8t, 1a94, 1a99, 1a9m, 1a9q, 1aaq, 1abf, 1add, 1adl, 1ado, 1af2, 1afk, 1afl, 1ag9, 1agm, 1ahx, 1ahy, 1ai4, 1ai5, 1ai6, 1ai7, 1aid, 1aj6, 1aj7, 1ajn, 1ajp, 1ajq, 1ajv, 1ajx, 1akq, 1akr, 1akt, 1aku, 1akv, 1akw, 1al7, 1al8, 1alw, 1amk, 1amw, 1anf, 1ao0, 1apb, 1apv, 1apw, 1aqc, 1aqi, 1aqj, 1at5, 1at6, 1atl, 1atr, 1avn, 1avp, 1awi, 1ax0, 1ax1, 1ax2, 1axr, 1axs, 1axz, 1azl, 1azm, 1azx, 1b05, 1b0h, 1b11, 1b1h, 1b2h, 1b32, 1b38, 1b39, 1b3f, 1b3g, 1b3h, 1b3l, 1b40, 1b42, 1b46, 1b4d, 1b4h, 1b4z, 1b51, 1b52, 1b55, 1b56, 1b57, 1b58, 1b5h, 1b5i, 1b5j, 1b6h, 1b6j, 1b6k, 1b6l, 1b6m, 1b6p, 1b7h, 1b8n, 1b8o, 1b8y, 1b9j, 1bai, 1bap, 1bbz, 1bcd, 1bcj, 1bdl, 1bdq, 1bdr, 1bgq, 1bhf, 1bhx, 1bju, 1bjv, 1bkj, 1bky, 1bm7, 1bma, 1bmm, 1bmn, 1bn1, 1bn3, 1bn4, 1bnm, 1bnn, 1bnq, 1bnt, 1bnu, 1bnv, 1bnw, 1bp0, 1bq3, 1bq4, 1br6, 1br8, 1bsk, 1bt6, 1btn, 1bty, 1bux, 1bv7, 1bv9, 1bwa, 1bwb, 1bwn, 1bxo, 1bxq, 1bxr, 1bzh, 1bzj, 1bzy, 1c12, 1c1r, 1c1u, 1c1v, 1c2t, 1c3e, 1c3x, 1c4u, 1c4v, 1c4y, 1c5c, 1c5n, 1c5o, 1c5p, 1c5q, 1c5s, 1c5t, 1c5x, 1c5y, 1c6y, 1c70, 1c7e, 1c7f, 1c83, 1c84, 1c85, 1c86, 1c87, 1c88, 1caq, 1cbr, 1cbx, 1ce5, 1cea, 1ceb, 1cet, 1cgl, 1ch8, 1ci7, 1cil, 1cim, 1cin, 1ciz, 1cka, 1ckb, 1clu, 1cnw, 1cnx, 1cny, 1cps, 1cr6, 1csh, 1csi, 1csr, 1css, 1ct8, 1ctr, 1ctt, 1ctu, 1cwb, 1czc, 1cze, 1czk, 1czl, 1czo, 1czr, 1d04, 1d09, 1d1p, 1d2e, 1d2s, 1d3d, 1d3p, 1d3q, 1d4h, 1d4i, 1d4j, 1d4k, 1d4l, 1d4p, 1d4t, 1d4w, 1d4y, 1d6n, 1d6s, 1d6v, 1d6w, 1d7i, 1d7j, 1d9i, 1dar, 1db1, 1dbb, 1det, 1df8, 1dfo, 1dg9, 1dgm, 1dhi, 1dhj, 1dif, 1dkd, 1dl7, 1dmb, 1dmp, 1dqn, 1dqx, 1drj, 1drk, 1drv, 1dub, 1dud, 1duv, 1dva, 1dwc, 1dwd, 1dxp, 1dy4, 1dzk, 1e03, 1e1v, 1e1x, 1e2k, 1e2l, 1e3g, 1e3v, 1e4h, 1e5a, 1e5j, 1e6q, 1e6s, 1e72, 1e8h, 1eb1, 1eb2, 1ebg, 1ebw, 1ebz, 1ec0, 1ec1, 1ec2, 1ec3, 1ec9, 1ecq, 1ecv, 1eed, 1efy, 1egh, 1eix, 1ej4, 1ejn, 1ek2, 1ela, 1elb, 1elc, 1eld, 1ele, 1elr, 1els, 1ent, 1enu, 1eoc, 1epo, 1epp, 1erb, 1ets, 1ett, 1etz, 1evh, 1ew8, 1ew9, 1ewj, 1ex8, 1ez9, 1ezq, 1f0r, 1f0s, 1f0t, 1f0u, 1f28, 1f3e, 1f47, 1f4e, 1f4f, 1f4g, 1f4x, 1f4y, 1f57, 1f5k, 1f5l, 1f73, 1f74, 1f8a, 1f8b, 1f8c, 1f8d, 1f8e, 1f90, 1f92, 1fao, 1fax, 1fbm, 1fch, 1fcx, 1fcy, 1fcz, 1fd0, 1fdq, 1fe3, 1fh7, 1fh8, 1fh9, 1fhd, 1fig, 1fiv, 1fj4, 1fjs, 1fkb, 1fkf, 1fkg, 1fkh, 1fki, 1fkn, 1fkw, 1fl3, 1fl6, 1flm, 1flr, 1fm9, 1fmb, 1fo0, 1fpc, 1fpp, 1fpy, 1fq4, 1fq5, 1fq6, 1fq7, 1fq8, 1ft7, 1ftm, 1fv0, 1fw0, 1fwe, 1fwu, 1fwv, 1fzj, 1fzk, 1fzm, 1fzo, 1fzq, 1g1d, 1g2l, 1g2o, 1g30, 1g32, 1g35, 1g36, 1g3b, 1g3c, 1g3d, 1g3e, 1g45, 1g46, 1g48, 1g4j, 1g4o, 1g50, 1g52, 1g53, 1g54, 1g5f, 1g6r, 1g6s, 1g74, 1g7f, 1g7g, 1g7p, 1g7q, 1g7v, 1g85, 1g98, 1g9r, 1g9s, 1g9t, 1gaf, 1gag, 1gah, 1gai, 1gar, 1gca, 1gcz, 1gfy, 1gfz, 1ghv, 1ghw, 1ghy, 1ghz, 1gi1, 1gi4, 1gi6, 1gi7, 1gi8, 1gi9, 1gj4, 1gj6, 1gj7, 1gj8, 1gja, 1gjb, 1gjc, 1gjd, 1gni, 1gnj, 1gnm, 1gnn, 1gno, 1gny, 1gpy, 1gqs, 1grp, 1gt1, 1gt3, 1gt4, 1gt5, 1gu1, 1gu3, 1gui, 1gux, 1gvu, 1gvw, 1gvx, 1gwm, 1gwq, 1gwr, 1gwv, 1gww, 1gx0, 1gx4, 1gx8, 1gyx, 1gyy, 1gz3, 1gz4, 1gz9, 1gzc, 1h0a, 1h0r, 1h1d, 1h1h, 1h1p, 1h1s, 1h24, 1h25, 1h26, 1h27, 1h2k, 1h2t, 1h2u, 1h46, 1h4n, 1h4w, 1h5v, 1h60, 1h61, 1h62, 1h6h, 1h79, 1h7a, 1h8l, 1h8s, 1h9z, 1ha2, 1hbv, 1hdq, 1hdt, 1hee, 1heg, 1hfs, 1hge, 1hgi, 1hgt, 1hi3, 1hi4, 1hi5, 1hih, 1hii, 1hiy, 1hk1, 1hk2, 1hk3, 1hk4, 1hk5, 1hlf, 1hlk, 1hmr, 1hms, 1hmt, 1hn2, 1hn4, 1hnn, 1hos, 1hp0, 1hp5, 1hpo, 1hps, 1hpv, 1hpx, 1hqf, 1hqg, 1hsg, 1hsh, 1hsl, 1hti, 1hv5, 1hvh, 1hvi, 1hvj, 1hvk, 1hvl, 1hvr, 1hvs, 1hwr, 1hxb, 1hxw, 1hyo, 1i00, 1i1e, 1i2s, 1i37, 1i3z, 1i5r, 1i7z, 1i80, 1i8i, 1i9l, 1i9m, 1i9n, 1i9o, 1i9p, 1i9q, 1ibg, 1icj, 1ie9, 1if7, 1if8, 1igb, 1igj, 1iht, 1ihy, 1ii5, 1iih, 1iiq, 1ik4, 1ikt, 1ilh, 1imx, 1ind, 1iq1, 1is0, 1it6, 1iup, 1ivp, 1iwq, 1iy7, 1izh, 1izi, 1j01, 1j07, 1j14, 1j15, 1j16, 1j17, 1j19, 1j36, 1j37, 1j4r, 1j7z, 1j80, 1j81, 1jak, 1jao, 1jaq, 1jcx, 1jd0, 1jd5, 1jd6, 1jet, 1jeu, 1jev, 1jfh, 1jgl, 1jh1, 1jif, 1jjk, 1jlr, 1jmf, 1jmg, 1jmi, 1jn2, 1jn4, 1joc, 1jp5, 1jpl, 1jq8, 1jq9, 1jqd, 1jqe, 1jqy, 1jr1, 1jsv, 1jt1, 1jtq, 1juj, 1juq, 1jut, 1juy, 1jvu, 1jwm, 1jws, 1jwt, 1jwu, 1jyc, 1jyq, 1jys, 1jzs, 1k03, 1k06, 1k08, 1k1j, 1k1l, 1k1m, 1k1n, 1k1o, 1k1y, 1k21, 1k22, 1k27, 1k4g, 1k4h, 1k6c, 1k6p, 1k6t, 1k6v, 1k9s, 1kav, 1kc7, 1kdk, 1kel, 1kf0, 1kf6, 1kjr, 1kl3, 1kl5, 1kll, 1km3, 1kmy, 1kna, 1kne, 1koj, 1kpm, 1kqb, 1kr3, 1ksn, 1kti, 1kug, 1kui, 1kuk, 1kv1, 1kv2, 1kv5, 1kyv, 1kzk, 1kzn, 1l0a, 1l2s, 1l5q, 1l5r, 1l5s, 1l6m, 1l7x, 1l83, 1l8g, 1laf, 1lag, 1lah, 1lan, 1lb6, 1lbf, 1lbk, 1lcj, 1lcp, 1lee, 1leg, 1lek, 1lf2, 1lf3, 1lf8, 1lf9, 1lgt, 1lgw, 1lhu, 1lhv, 1lhw, 1li2, 1li3, 1li6, 1lke, 1lkk, 1lkl, 1lkx, 1ll4, 1lnm, 1lol, 1loq, 1lor, 1los, 1lox, 1lpk, 1lpz, 1lq2, 1lqd, 1lqe, 1lrh, 1lrt, 1lst, 1lv8, 1lvu, 1lyb, 1lyx, 1lzo, 1lzq, 1m0b, 1m0n, 1m0o, 1m0q, 1m13, 1m1b, 1m2p, 1m2q, 1m2r, 1m2x, 1m48, 1m4h, 1m5w, 1m6p, 1m74, 1m7d, 1m7i, 1m7y, 1m83, 1m9n, 1mai, 1mau, 1mcz, 1mdl, 1me7, 1me8, 1mes, 1met, 1meu, 1mfa, 1mfd, 1mfg, 1mfi, 1mh5, 1mhw, 1mj7, 1mjj, 1ml1, 1mmp, 1mmq, 1mmr, 1mn9, 1moq, 1mpa, 1mq5, 1mrn, 1mrs, 1mrw, 1mrx, 1msm, 1msn, 1mtr, 1mu6, 1mu8, 1mue, 1mui, 1my2, 1my4, 1n0s, 1n1m, 1n1t, 1n1v, 1n2v, 1n3i, 1n3w, 1n3z, 1n46, 1n4h, 1n4k, 1n4m, 1n51, 1n5r, 1n5z, 1n7i, 1n7j, 1n7m, 1n8u, 1n8v, 1nax, 1nde, 1ndv, 1ndw, 1ndy, 1ndz, 1nf8, 1nfu, 1nfw, 1nfx, 1nfy, 1ngw, 1nh0, 1nhg, 1nhu, 1nhv, 1nhw, 1nhz, 1nj1, 1nj5, 1nja, 1njb, 1njc, 1njd, 1nje, 1njf, 1njj, 1njs, 1nki, 1nl9, 1nli, 1nlt, 1nm6, 1nms, 1nnb, 1nnu, 1nny, 1no6, 1noi, 1noj, 1nok, 1nox, 1np0, 1nq7, 1nt1, 1ntk, 1ntv, 1nu3, 1nvr, 1nvs, 1nw4, 1nw5, 1nw7, 1nwl, 1ny2, 1nz7, 1nzl, 1nzv, 1o0f, 1o0m, 1o0n, 1o0o, 1o1s, 1o2g, 1o2h, 1o2j, 1o2k, 1o2n, 1o2o, 1o2p, 1o2q, 1o2r, 1o2s, 1o2u, 1o2v, 1o2w, 1o2x, 1o2y, 1o2z, 1o30, 1o32, 1o33, 1o34, 1o35, 1o36, 1o37, 1o38, 1o39, 1o3b, 1o3c, 1o3d, 1o3e, 1o3g, 1o3h, 1o3i, 1o3j, 1o3k, 1o3l, 1o3p, 1o5a, 1o5c, 1o5e, 1o5f, 1o5g, 1o5r, 1o6i, 1o7o, 1o86, 1o8b, 1o9d, 1o9e, 1o9k, 1oai, 1oar, 1oau, 1oay, 1oba, 1obx, 1ocn, 1ocq, 1od8, 1odi, 1odj, 1ody, 1oe7, 1oe8, 1oeb, 1ofz, 1ogd, 1ogg, 1ogx, 1ogz, 1oh4, 1ohr, 1oif, 1oim, 1oj5, 1ok7, 1okl, 1oko, 1ols, 1olu, 1olx, 1om1, 1om9, 1ony, 1onz, 1ork, 1os0, 1os5, 1osg, 1oss, 1osv, 1oth, 1ov3, 1ow4, 1ow6, 1ow7, 1ow8, 1owd, 1owe, 1owi, 1owk, 1ox9, 1oxn, 1oxq, 1oxr, 1oy7, 1oyq, 1oz0, 1p17, 1p19, 1p1o, 1p28, 1p2g, 1p4r, 1p4u, 1p57, 1p6d, 1p6e, 1p93, 1pa9, 1pb8, 1pb9, 1pbk, 1pbq, 1pcg, 1pdq, 1pdz, 1pf7, 1pf8, 1pfu, 1pg2, 1pgp, 1ph0, 1phw, 1pkx, 1pl0, 1pme, 1pot, 1ppc, 1pph, 1ppi, 1ppk, 1ppl, 1ppm, 1pr1, 1pr5, 1pro, 1pu7, 1pu8, 1pvn, 1pwp, 1pwu, 1pwy, 1px4, 1pxh, 1pxm, 1pxo, 1pxp, 1py1, 1pye, 1pyg, 1pyn, 1pz5, 1pzi, 1pzo, 1pzp, 1q1g, 1q1m, 1q4k, 1q4l, 1q4w, 1q4x, 1q54, 1q5k, 1q63, 1q65, 1q66, 1q72, 1q7a, 1q84, 1q8w, 1q91, 1q95, 1q9d, 1qan, 1qaq, 1qaw, 1qb1, 1qb6, 1qb9, 1qbn, 1qbo, 1qbq, 1qbr, 1qbs, 1qbt, 1qbu, 1qbv, 1qca, 1qf0, 1qf2, 1qft, 1qhc, 1qi0, 1qin, 1qiw, 1qja, 1qjb, 1qji, 1qk3, 1qk4, 1qka, 1qkb, 1ql7, 1ql9, 1qm4, 1qm5, 1qng, 1qnh, 1qon, 1qq9, 1qsc, 1qvt, 1qvu, 1qw7, 1qx1, 1qxk, 1qxl, 1qy1, 1qy2, 1qyg, 1r0p, 1r0x, 1r10, 1r17, 1r1h, 1r1i, 1r1j, 1r2b, 1r4w, 1r5n, 1r5v, 1r5w, 1r6n, 1r9l, 1rbp, 1rd4, 1rdi, 1rdl, 1rdt, 1re8, 1rej, 1rek, 1rev, 1rgk, 1rgl, 1rin, 1rjk, 1rm8, 1rmz, 1rnm, 1rnt, 1ro6, 1ro7, 1rp7, 1rpa, 1rpf, 1rpj, 1rql, 1rr6, 1rst, 1rt9, 1rtf, 1ru1, 1ru2, 1ryf, 1ryh, 1rzx, 1s19, 1s26, 1s39, 1s3k, 1s4d, 1s50, 1s5z, 1s63, 1s64, 1s89, 1sb1, 1sbg, 1sbr, 1sc8, 1sdt, 1sdu, 1sdv, 1sfi, 1sgu, 1sh9, 1siv, 1sje, 1sjh, 1sl3, 1sld, 1sle, 1slg, 1sln, 1sm3, 1sme, 1sps, 1sqc, 1sqn, 1sqo, 1sqq, 1sqt, 1sr7, 1srg, 1sri, 1ssq, 1stc, 1std, 1str, 1sts, 1sv3, 1sw1, 1sw2, 1swg, 1swi, 1swr, 1syh, 1szd, 1t08, 1t13, 1t29, 1t31, 1t32, 1t37, 1t3t, 1t4e, 1t4s, 1t4v, 1t5a, 1t5f, 1t69, 1t79, 1t7d, 1t7f, 1t7j, 1t7r, 1ta2, 1ta6, 1tcw, 1tcx, 1td7, 1tet, 1thl, 1thz, 1tjp, 1tka, 1tkb, 1tkc, 1tl7, 1tlp, 1tmm, 1tmn, 1tng, 1tnh, 1tni, 1tog, 1toi, 1toj, 1tok, 1tom, 1tpw, 1tpz, 1tq4, 1tr7, 1trd, 1tsi, 1tsl, 1tsm, 1tsv, 1tsy, 1ttm, 1tuf, 1tvo, 1tx7, 1txr, 1tyr, 1tys, 1u0g, 1u0h, 1u1w, 1u2r, 1u33, 1u65, 1u6q, 1u71, 1u8t, 1u9l, 1ua4, 1ucn, 1udt, 1udu, 1uef, 1ugp, 1ugw, 1ugx, 1ugy, 1uh1, 1uho, 1ui0, 1uj0, 1uj5, 1uj6, 1ujj, 1ujk, 1ukh, 1ule, 1ulg, 1uml, 1umw, 1uou, 1upf, 1upk, 1ur9, 1urg, 1usi, 1usk, 1usn, 1utc, 1uti, 1utj, 1utl, 1utm, 1utn, 1uv6, 1uvs, 1uvt, 1uvu, 1uw6, 1uwf, 1uwt, 1uwu, 1ux7, 1uxa, 1uxb, 1uys, 1uz1, 1uz4, 1uz8, 1v0k, 1v0l, 1v0m, 1v0n, 1v11, 1v16, 1v1j, 1v1m, 1v2j, 1v2k, 1v2l, 1v2m, 1v2n, 1v2o, 1v2p, 1v2q, 1v2r, 1v2s, 1v2t, 1v2u, 1v2v, 1v2w, 1v41, 1v48, 1v79, 1v7a, 1vcu, 1vea, 1vfn, 1vj9, 1vja, 1vjc, 1vjd, 1vkj, 1vot, 1vr1, 1vwf, 1vwl, 1vwn, 1vyf, 1vyg, 1vyj, 1vyq, 1vzq, 1w0y, 1w0z, 1w11, 1w13, 1w1d, 1w1g, 1w25, 1w2g, 1w2h, 1w2k, 1w2x, 1w3j, 1w3k, 1w3l, 1w4p, 1w4q, 1w5v, 1w5w, 1w5x, 1w5y, 1w6h, 1w6y, 1w70, 1w7g, 1w7x, 1w80, 1w96, 1w9u, 1w9v, 1wc1, 1wc6, 1wcq, 1wdn, 1wdr, 1we2, 1wht, 1wm1, 1wn6, 1ws1, 1ws4, 1ws5, 1wuq, 1wur, 1wvc, 1wvj, 1x07, 1x11, 1x1z, 1x38, 1x39, 1x6u, 1x7a, 1x8d, 1x8j, 1x8r, 1x8s, 1x8t, 1x9d, 1xa5, 1xap, 1xb7, 1xbb, 1xbo, 1xd0, 1xff, 1xge, 1xgi, 1xgj, 1xh4, 1xh5, 1xh6, 1xh7, 1xh9, 1xhy, 1xjd, 1xk5, 1xk9, 1xka, 1xkk, 1xn2, 1xn3, 1xnx, 1xow, 1xpz, 1xq0, 1xr8, 1xr9, 1xs7, 1xt3, 1xt8, 1xug, 1xws, 1xz8, 1xzx, 1y0l, 1y1z, 1y20, 1y2a, 1y2f, 1y2g, 1y3a, 1y3n, 1y3p, 1y3v, 1y3w, 1y3x, 1y3y, 1y4z, 1y6q, 1y8o, 1y8p, 1y98, 1ybg, 1ybo, 1yc4, 1yci, 1yda, 1ydb, 1ydd, 1ydk, 1yds, 1yei, 1yej, 1yet, 1yfz, 1yid, 1ykp, 1yon, 1yp9, 1ype, 1ypg, 1ypj, 1yq7, 1yqj, 1yqy, 1yvh, 1yvm, 1ywh, 1yxd, 1yy6, 1yyr, 1yys, 1yz3, 1z1h, 1z1r, 1z3t, 1z3v, 1z4n, 1z4o, 1z6d, 1z6s, 1z71, 1z9y, 1zc9, 1zdp, 1ze8, 1zea, 1zfk, 1zfq, 1zge, 1zgi, 1zhk, 1zhl, 1zhy, 1zkk, 1zky, 1zm6, 1zoe, 1zog, 1zoh, 1zp8, 1zpa, 1zs0, 1zsf, 1zsh, 1zsr, 1zvx, 1zxv, 1zyr, 220l, 223l, 2a14, 2a25, 2a2g, 2a3c, 2a4m, 2a4w, 2a58, 2a5b, 2a5c, 2a5s, 2a8g, 2aa6, 2aac, 2ad5, 2adm, 2adu, 2ael, 2afw, 2afx, 2agv, 2aj8, 2am4, 2amt, 2amv, 2an5, 2ans, 2ao6, 2aoc, 2aod, 2aoe, 2aof, 2aog, 2aoh, 2aoi, 2aoj, 2aou, 2aov, 2aow, 2aq9, 2aqu, 2arm, 2asu, 2ate, 2avm, 2avo, 2avq, 2avs, 2avv, 2aw1, 2ax6, 2ax9, 2ay1, 2ay3, 2ay4, 2ay5, 2ay6, 2ay7, 2ay8, 2ay9, 2ayr, 2az5, 2azm, 2azr, 2b07, 2b17, 2b1g, 2b1i, 2b1v, 2b2v, 2b4l, 2b4m, 2b7d, 2b7f, 2b9a, 2baj, 2bak, 2bal, 2bba, 2bes, 2bet, 2bfq, 2bfr, 2bgr, 2bj4, 2bjm, 2bmg, 2bmk, 2bmv, 2bmz, 2bo4, 2boh, 2boj, 2bok, 2bpv, 2bpx, 2bpy, 2bq6, 2bq7, 2bqv, 2bqw, 2br6, 2br8, 2brm, 2bt9, 2buv, 2bvd, 2bvr, 2bvs, 2bxt, 2bxu, 2byp, 2byr, 2bys, 2bz6, 2bz8, 2bza, 2bzz, 2c02, 2c1n, 2c1p, 2c3j, 2c3k, 2c3l, 2c4v, 2c4w, 2c6n, 2c6o, 2c80, 2c92, 2c94, 2c97, 2c9b, 2c9d, 2ca8, 2cbj, 2cbu, 2cbz, 2cc7, 2ccb, 2ccc, 2ce9, 2cej, 2cem, 2cen, 2ceo, 2ceq, 2cer, 2ces, 2cex, 2cf8, 2cf9, 2cgf, 2cgr, 2cht, 2ci9, 2cia, 2cji, 2cle, 2clf, 2clh, 2cli, 2clk, 2cll, 2clm, 2clo, 2clv, 2cmo, 2cn0, 2co0, 2csm, 2csn, 2ctc, 2cvd, 2d06, 2d0k, 2d1n, 2d1o, 2d3u, 2d3z, 2d41, 2df6, 2doo, 2drc, 2dri, 2dua, 2dw7, 2dwx, 2e1w, 2e27, 2e2p, 2e2r, 2e5y, 2e7f, 2e7l, 2e91, 2e92, 2e93, 2e94, 2e95, 2e9u, 2e9v, 2eg7, 2eg8, 2eh8, 2emt, 2epn, 2er0, 2er6, 2er9, 2erz, 2etk, 2etr, 2euk, 2eum, 2evl, 2ewa, 2ewb, 2ews, 2exm, 2ez7, 2f0z, 2f10, 2f14, 2f1g, 2f2h, 2f34, 2f35, 2f5t, 2f6j, 2f6t, 2f7i, 2f7o, 2f7p, 2f80, 2f81, 2f89, 2f8g, 2f8i, 2f94, 2f9b, 2f9k, 2fai, 2fb8, 2fdp, 2ff1, 2ff2, 2fgh, 2fgi, 2fgu, 2fgv, 2fhy, 2fkf, 2fl5, 2flb, 2fle, 2flh, 2flr, 2flu, 2fmb, 2fo4, 2fpz, 2fqo, 2fqt, 2fqw, 2fqx, 2fqy, 2fr3, 2fr8, 2fsa, 2fsv, 2fts, 2fu8, 2fw6, 2fwp, 2fx6, 2fx7, 2fx8, 2fx9, 2fxu, 2fxv, 2fys, 2fzc, 2fzg, 2fzk, 2fzz, 2g00, 2g2r, 2g5u, 2g6q, 2g70, 2g71, 2g72, 2g78, 2g79, 2g8n, 2g8r, 2g94, 2g96, 2g97, 2g9q, 2gej, 2gek, 2gfa, 2gfd, 2gh9, 2gj5, 2gkl, 2gl0, 2glm, 2glp, 2gmk, 2gph, 2gss, 2gst, 2gsu, 2gv6, 2gv7, 2gvd, 2gvj, 2gvv, 2gyi, 2gz2, 2gzl, 2h13, 2h15, 2h21, 2h23, 2h2d, 2h2g, 2h2h, 2h2j, 2h3e, 2h4g, 2h4k, 2h4n, 2h5a, 2h5e, 2h6b, 2h6k, 2h6q, 2h6t, 2h9m, 2h9n, 2h9p, 2ha2, 2ha3, 2ha4, 2ha5, 2ha6, 2ha7, 2hah, 2haw, 2hb3, 2hd6, 2hds, 2hdu, 2hdx, 2hf8, 2hh5, 2hhn, 2hj4, 2hjb, 2hkf, 2hl4, 2hmh, 2hmu, 2hmv, 2hmw, 2hnc, 2hnx, 2hoc, 2hpa, 2hqu, 2hr6, 2hrm, 2hrp, 2hs1, 2hs2, 2hu6, 2hvc, 2hw2, 2hwg, 2hxm, 2hzl, 2hzy, 2i0a, 2i0e, 2i0g, 2i0j, 2i19, 2i2b, 2i2c, 2i3h, 2i3i, 2i3v, 2i3z, 2i4d, 2i4j, 2i4p, 2i4t, 2i4u, 2i4v, 2i4w, 2i4x, 2i4z, 2i6b, 2i80, 2idk, 2idw, 2ien, 2ieo, 2ig0, 2igv, 2ihj, 2ihq, 2iko, 2iku, 2il2, 2ima, 2imd, 2ioa, 2ipo, 2isc, 2isv, 2isw, 2itk, 2itp, 2itt, 2itz, 2iu0, 2iuz, 2iv9, 2ivu, 2ivz, 2iw4, 2iws, 2iwu, 2iyf, 2izl, 2izx, 2j27, 2j2u, 2j34, 2j3q, 2j47, 2j4g, 2j4i, 2j4k, 2j4q, 2j62, 2j75, 2j77, 2j79, 2j7b, 2j7d, 2j7e, 2j7f, 2j7g, 2j94, 2j95, 2j9h, 2j9l, 2j9n, 2jb5, 2jb6, 2jbj, 2jbk, 2jdh, 2jdk, 2jdm, 2jdn, 2jdp, 2jds, 2jdu, 2jdv, 2jdy, 2jew, 2jf4, 2jfh, 2jfz, 2jg0, 2jg8, 2jgs, 2jh0, 2jh5, 2jh6, 2jiw, 2jj3, 2jjb, 2jk7, 2jk9, 2jke, 2jkh, 2jkp, 2jkr, 2jxr, 2kce, 2mas, 2mpa, 2nm1, 2nmx, 2nmy, 2nmz, 2nn1, 2nn7, 2nn8, 2nnd, 2nng, 2nnk, 2nno, 2nnp, 2nnq, 2nns, 2nnv, 2np9, 2nsj, 2nsl, 2nt7, 2nta, 2nw4, 2nwl, 2nwn, 2nxd, 2nxl, 2nxm, 2o0u, 2o1c, 2o1v, 2o2u, 2o3z, 2o4h, 2o4j, 2o4k, 2o4l, 2o4n, 2o4p, 2o4r, 2o4s, 2o4z, 2o7e, 2o8h, 2o9j, 2o9k, 2o9v, 2oag, 2oax, 2oaz, 2obf, 2oc2, 2oc4, 2oc9, 2oei, 2ogy, 2ohl, 2oi0, 2oi2, 2oiq, 2ojg, 2oji, 2ojj, 2ok1, 2olb, 2ole, 2on3, 2on6, 2onb, 2ony, 2onz, 2op9, 2opb, 2opy, 2oqi, 2oqv, 2or9, 2osf, 2osm, 2ot1, 2ov4, 2ovq, 2ovv, 2ovy, 2ovz, 2oxd, 2oxn, 2oxx, 2oxy, 2oyk, 2oyl, 2oym, 2oz5, 2oz6, 2oz7, 2p09, 2p0d, 2p16, 2p1c, 2p2a, 2p3a, 2p3b, 2p3c, 2p3d, 2p3i, 2p4j, 2p4s, 2p53, 2p7a, 2p7g, 2p7z, 2p8n, 2p93, 2p94, 2p95, 2pbw, 2pcp, 2peh, 2pem, 2pfy, 2pgz, 2ph9, 2phb, 2pk5, 2pk6, 2pl0, 2pl9, 2pmc, 2pmk, 2pnc, 2pnx, 2pou, 2pov, 2pow, 2pq9, 2pqb, 2pqc, 2pqj, 2pql, 2pqz, 2pr9, 2pri, 2prj, 2psj, 2psu, 2psv, 2ptz, 2pu0, 2pu1, 2pu2, 2puy, 2pv1, 2pv2, 2pvh, 2pvj, 2pvk, 2pvl, 2pvm, 2pvn, 2pvu, 2pwc, 2pwd, 2pwg, 2pwr, 2py4, 2pyi, 2pym, 2pyn, 2pyy, 2pze, 2q11, 2q15, 2q1q, 2q2a, 2q2c, 2q2n, 2q38, 2q54, 2q55, 2q5k, 2q63, 2q64, 2q6f, 2q70, 2q7o, 2q7q, 2q7y, 2q80, 2q88, 2q89, 2q8h, 2q8m, 2q8y, 2q8z, 2q9y, 2qbs, 2qbu, 2qbw, 2qbx, 2qci, 2qd6, 2qd7, 2qd8, 2qdt, 2qfo, 2qft, 2qfu, 2qg0, 2qg2, 2qhc, 2qhd, 2qhm, 2qhr, 2qhy, 2qhz, 2qi0, 2qi1, 2qi3, 2qi4, 2qi5, 2qi6, 2qi7, 2qic, 2qki, 2qlm, 2qln, 2qm7, 2qm9, 2qmg, 2qmj, 2qn3, 2qnb, 2qnn, 2qnp, 2qo8, 2qoa, 2qp6, 2qpq, 2qpu, 2qqs, 2qrg, 2qrh, 2qrk, 2qrl, 2qrm, 2qrp, 2qrq, 2qry, 2qt5, 2qta, 2qtg, 2qtn, 2qtr, 2qtt, 2qtu, 2qu5, 2qu6, 2qv7, 2qw1, 2qwb, 2qwc, 2qwd, 2qwe, 2qwf, 2qx0, 2qzr, 2r02, 2r03, 2r05, 2r0h, 2r0y, 2r0z, 2r1w, 2r1x, 2r1y, 2r23, 2r2b, 2r2m, 2r2w, 2r38, 2r3t, 2r3w, 2r3y, 2r43, 2r58, 2r59, 2r5a, 2r5b, 2r5d, 2r5p, 2r5q, 2r6w, 2r6y, 2r75, 2r7g, 2r9b, 2r9x, 2ra0, 2ra6, 2rcb, 2rcn, 2rcw, 2rd6, 2reg, 2rfh, 2rfy, 2ri9, 2rin, 2rio, 2rk7, 2rk8, 2rka, 2rkd, 2rke, 2rkf, 2rkg, 2rkm, 2rkn, 2sim, 2std, 2tmn, 2toh, 2tpi, 2tsr, 2upj, 2usn, 2uvm, 2uwd, 2uwl, 2uwo, 2uwp, 2uxi, 2uxu, 2uxx, 2uxz, 2uy0, 2uy3, 2uy4, 2uy5, 2uyn, 2uyq, 2uz6, 2uz9, 2v0n, 2v25, 2v2c, 2v2h, 2v2q, 2v2v, 2v3d, 2v3e, 2v3u, 2v54, 2v57, 2v58, 2v59, 2v5x, 2v77, 2v7d, 2v83, 2v85, 2v86, 2v87, 2v88, 2v8w, 2v95, 2vb8, 2vba, 2vc7, 2vc9, 2ves, 2vfk, 2vfz, 2vh0, 2vh6, 2vhj, 2vhq, 2vi5, 2vj8, 2vjx, 2vk2, 2vk6, 2vl4, 2vmc, 2vmd, 2vmf, 2vnf, 2vnp, 2vnt, 2vo4, 2vo5, 2vot, 2vpe, 2vpg, 2vpn, 2vpo, 2vqt, 2vr0, 2vr3, 2vr4, 2vrj, 2vsl, 2vt3, 2vuk, 2vvc, 2vvp, 2vvs, 2vvu, 2vvv, 2vw1, 2vw2, 2vwc, 2vwf, 2vwl, 2vwm, 2vwn, 2vwo, 2vx9, 2vxa, 2vxj, 2vxn, 2vyt, 2vzr, 2w08, 2w0p, 2w0s, 2w0z, 2w10, 2w16, 2w26, 2w2i, 2w2u, 2w3o, 2w47, 2w5g, 2w5i, 2w67, 2w68, 2w6t, 2w6u, 2w73, 2w76, 2w77, 2w78, 2w7y, 2w87, 2w8f, 2w8g, 2w8j, 2w8w, 2w8y, 2w9h, 2w9r, 2wa8, 2wb5, 2wc3, 2wc4, 2wcg, 2wd7, 2we3, 2web, 2wec, 2wed, 2weh, 2wej, 2weo, 2weq, 2wf5, 2wfj, 2wgj, 2whp, 2wib, 2wic, 2wjg, 2wji, 2wk2, 2wk6, 2wks, 2wkt, 2wky, 2wkz, 2wl0, 2wl4, 2wl5, 2wly, 2wlz, 2wm0, 2wnj, 2wnl, 2won, 2wor, 2wos, 2wp1, 2wpb, 2wq5, 2wqp, 2wr8, 2wtx, 2wuf, 2wva, 2wvz, 2ww0, 2ww2, 2wyf, 2wyg, 2wyi, 2wyj, 2wyn, 2wzf, 2wzm, 2wzs, 2wzy, 2x09, 2x0y, 2x2c, 2x2i, 2x2r, 2x4z, 2x6w, 2x6x, 2x6y, 2x7s, 2x7t, 2x7u, 2x7x, 2x85, 2x8z, 2x91, 2x95, 2x96, 2x97, 2xab, 2xb7, 2xb9, 2xba, 2xbp, 2xbw, 2xbx, 2xc0, 2xc4, 2xcg, 2xd9, 2xda, 2xde, 2xdk, 2xdx, 2xef, 2xeg, 2xei, 2xej, 2xg3, 2xg9, 2xgm, 2xgo, 2xgs, 2xhm, 2xhr, 2xhs, 2xht, 2xhx, 2xi7, 2xib, 2xj1, 2xj2, 2xjg, 2xjj, 2xjx, 2xl2, 2xl3, 2xm1, 2xm2, 2xmy, 2xn3, 2xn5, 2xn6, 2xog, 2xoi, 2xp7, 2xpc, 2xpk, 2xqq, 2xrw, 2xs0, 2xs8, 2xtn, 2xto, 2xuz, 2xv1, 2xwd, 2xwe, 2xxn, 2xxr, 2xxt, 2xxw, 2xxx, 2xxy, 2xy9, 2xyd, 2xye, 2xyf, 2xyn, 2xyt, 2xz5, 2xzq, 2xzw, 2y06, 2y07, 2y1n, 2y1w, 2y34, 2y36, 2y3p, 2y4k, 2y4l, 2y4s, 2y5f, 2y5g, 2y6s, 2y71, 2y76, 2y77, 2y7i, 2y7k, 2y7p, 2y7w, 2y7x, 2y7z, 2y80, 2y81, 2y82, 2y8c, 2y8l, 2y8o, 2y8q, 2y9g, 2y9q, 2ya6, 2ya7, 2ya8, 2yay, 2yaz, 2yb0, 2ybu, 2ydf, 2ydo, 2ydt, 2ydv, 2ydw, 2yek, 2yel, 2yfa, 2yfx, 2yga, 2ygf, 2ygu, 2yhd, 2yhw, 2yhy, 2yi0, 2yi5, 2yi7, 2yim, 2yiv, 2yix, 2yjq, 2yk1, 2yln, 2yme, 2ynd, 2yne, 2ynn, 2ynr, 2yns, 2yof, 2yog, 2yoh, 2ypi, 2ypo, 2ypp, 2yq6, 2yxj, 2yz3, 2z1w, 2z3h, 2z4b, 2z4o, 2z5s, 2z5t, 2z6w, 2z7g, 2z7h, 2z8e, 2z92, 2z94, 2z9g, 2za0, 2za3, 2za5, 2zaz, 2zb0, 2zc9, 2zcs, 2zdk, 2zdl, 2zdm, 2zdn, 2zfp, 2zfs, 2zft, 2zg1, 2zg3, 2zga, 2zgx, 2zhd, 2zif, 2zit, 2zju, 2zjv, 2zjw, 2zkj, 2zm3, 2zmd, 2zmm, 2zn7, 2zns, 2znt, 2znu, 2zo3, 2zpk, 2zq0, 2zq1, 2zq2, 2zsc, 2zwz, 2zx6, 2zx7, 2zx8, 2zx9, 2zxa, 2zxb, 2zxd, 2zxg, 2zym, 2zyn, 2zz1, 2zz2, 3a1c, 3a1d, 3a1e, 3a1s, 3a1t, 3a2c, 3a2o, 3a3y, 3a5y, 3a6t, 3a73, 3a9i, 3aaq, 3aas, 3aau, 3acl, 3acx, 3ad7, 3ad8, 3ads, 3adt, 3adu, 3adv, 3afk, 3agl, 3agm, 3ahn, 3aho, 3ai8, 3aid, 3aje, 3al3, 3alt, 3ama, 3amb, 3amv, 3anq, 3ao1, 3ao2, 3ao5, 3aox, 3ap4, 3ap7, 3aqa, 3aqt, 3arb, 3are, 3arf, 3arg, 3arr, 3art, 3arw, 3arx, 3arz, 3as0, 3ask, 3asl, 3at1, 3atp, 3atu, 3atv, 3ax5, 3axk, 3axm, 3axz, 3ay9, 3aya, 3ayc, 3ayd, 3b24, 3b25, 3b26, 3b28, 3b2q, 3b2t, 3b3c, 3b3s, 3b3w, 3b3x, 3b4f, 3b4p, 3b50, 3b5j, 3b66, 3b67, 3b78, 3b7i, 3b7j, 3b7r, 3b7u, 3b82, 3b8h, 3b92, 3b95, 3b9g, 3bb1, 3bbb, 3bbf, 3bbt, 3bc3, 3bc4, 3bcs, 3be9, 3bex, 3bf1, 3bft, 3bfu, 3bgb, 3bgc, 3bgp, 3bgq, 3bgs, 3bim, 3bkk, 3bkl, 3bl0, 3bl1, 3bl2, 3bla, 3blr, 3bmn, 3bmo, 3bmq, 3bpc, 3bqc, 3bra, 3brn, 3bt9, 3btc, 3bti, 3btj, 3btl, 3btr, 3bu1, 3bu6, 3bu8, 3buf, 3bug, 3buh, 3bum, 3bun, 3buo, 3buw, 3bux, 3bva, 3bvb, 3bwj, 3bxe, 3bxf, 3bxg, 3bxh, 3bxs, 3bxz, 3bze, 3bzf, 3bzi, 3c14, 3c1k, 3c1n, 3c2f, 3c2o, 3c2r, 3c2u, 3c39, 3c3o, 3c3q, 3c3r, 3c49, 3c4h, 3c52, 3c56, 3c6w, 3c79, 3c84, 3c88, 3c89, 3c8a, 3c8b, 3c8e, 3c94, 3caj, 3cct, 3ccw, 3ccz, 3cd0, 3cd5, 3cd7, 3cda, 3cdb, 3ce0, 3cen, 3cf8, 3cf9, 3cfn, 3cfs, 3cft, 3cfv, 3cgy, 3chg, 3cj2, 3cj5, 3ck7, 3ck8, 3ckb, 3cke, 3ckp, 3ckr, 3ckt, 3ckz, 3cl0, 3cl2, 3clp, 3cm2, 3cow, 3cph, 3cpj, 3cr4, 3cr5, 3cs7, 3cso, 3ctr, 3ctt, 3cwk, 3cx9, 3cyu, 3cyw, 3cyx, 3cyy, 3cyz, 3cz1, 3czv, 3d0b, 3d0e, 3d1e, 3d1f, 3d1g, 3d1v, 3d1x, 3d1y, 3d1z, 3d20, 3d2e, 3d2r, 3d2t, 3d32, 3d3x, 3d4y, 3d50, 3d51, 3d52, 3d6o, 3d6p, 3d78, 3d7b, 3d7k, 3d7m, 3d7z, 3d83, 3d8w, 3d8y, 3d8z, 3d91, 3d94, 3d9k, 3d9l, 3d9m, 3d9n, 3d9o, 3d9p, 3d9z, 3da9, 3dab, 3daz, 3dba, 3dbu, 3dc2, 3dc3, 3dcc, 3dcs, 3dcw, 3dd8, 3ddf, 3ddg, 3dgl, 3dgn, 3dgo, 3dhk, 3dhv, 3diw, 3djk, 3djo, 3djp, 3djq, 3djv, 3djx, 3dk1, 3dkf, 3dla, 3dln, 3dnd, 3dne, 3dnj, 3dnt, 3dow, 3dp4, 3dp9, 3dpc, 3dpo, 3drf, 3drg, 3dri, 3ds0, 3ds1, 3ds3, 3ds4, 3ds9, 3dst, 3dsu, 3dsz, 3dux, 3dvp, 3dx0, 3dx3, 3dx4, 3dxh, 3dxj, 3dxk, 3dxm, 3dyo, 3dzt, 3e12, 3e1r, 3e3c, 3e4a, 3e5u, 3e64, 3e6y, 3e73, 3e85, 3e8r, 3e8u, 3e9h, 3e9i, 3eax, 3eb1, 3ebb, 3ebh, 3ebi, 3ebl, 3ebo, 3ed0, 3eeb, 3efj, 3efk, 3efr, 3efs, 3eft, 3eg6, 3egk, 3egt, 3ehx, 3eig, 3ejp, 3ejq, 3eko, 3ekp, 3ekq, 3ekr, 3eks, 3ekt, 3ekv, 3ekw, 3ekx, 3eky, 3el0, 3el1, 3el4, 3el5, 3el9, 3elc, 3elm, 3emg, 3emh, 3eml, 3eos, 3eou, 3eov, 3eqr, 3eqs, 3eqy, 3er3, 3er5, 3erd, 3ert, 3ery, 3esj, 3eu5, 3eu7, 3evc, 3evd, 3evf, 3ew2, 3ewc, 3ewj, 3exe, 3exf, 3exh, 3eyf, 3eys, 3eyu, 3f0r, 3f15, 3f16, 3f17, 3f18, 3f19, 3f1a, 3f33, 3f34, 3f35, 3f36, 3f37, 3f38, 3f3t, 3f3u, 3f3v, 3f48, 3f5j, 3f5k, 3f5l, 3f68, 3f6e, 3f6g, 3f6h, 3f70, 3f78, 3f7g, 3f7h, 3f7i, 3f80, 3f82, 3f8c, 3f8e, 3f8f, 3f9w, 3f9y, 3fa3, 3fas, 3fat, 3fdm, 3fdt, 3fe7, 3fea, 3fed, 3fee, 3feg, 3ff3, 3ffg, 3ffp, 3ffu, 3fgc, 3fh7, 3fhb, 3fj7, 3fjg, 3fjz, 3fk1, 3fl5, 3fmz, 3fn0, 3fqe, 3fql, 3fsj, 3fsm, 3ftq, 3fuc, 3fuz, 3fv3, 3fvg, 3fvh, 3fvk, 3fvl, 3fvn, 3fw4, 3fwv, 3fx6, 3fxb, 3fzn, 3fzy, 3g08, 3g0e, 3g0f, 3g0i, 3g15, 3g19, 3g1d, 3g1v, 3g2h, 3g2i, 3g2j, 3g2k, 3g2l, 3g2s, 3g2t, 3g2u, 3g2v, 3g2w, 3g2y, 3g30, 3g32, 3g34, 3g35, 3g3r, 3g5d, 3g5k, 3g5v, 3g5y, 3g7l, 3g8e, 3g90, 3g9l, 3g9n, 3ga5, 3gba, 3gbe, 3gc4, 3gcp, 3gcq, 3gcs, 3gcu, 3gcv, 3gds, 3gdt, 3gep, 3gfw, 3ggc, 3ggj, 3ggu, 3ggw, 3ghe, 3gi4, 3gi5, 3gi6, 3gjd, 3gjw, 3gk1, 3gk2, 3gk4, 3gkz, 3gl6, 3gm0, 3gn7, 3gp0, 3gpo, 3gqo, 3gqz, 3grj, 3gs6, 3gsg, 3gsm, 3gss, 3gst, 3gt9, 3gta, 3gtc, 3gus, 3guz, 3gvb, 3gvu, 3gws, 3gx0, 3gxt, 3gxy, 3gxz, 3gy2, 3gy3, 3gy7, 3h0a, 3h1x, 3h1z, 3h30, 3h52, 3h5b, 3h6z, 3h78, 3h85, 3h89, 3h8b, 3h8c, 3h91, 3h9f, 3h9k, 3hau, 3hav, 3haw, 3hb4, 3hb8, 3hbo, 3hcm, 3hdk, 3hec, 3heg, 3hek, 3hf8, 3hfb, 3hfv, 3hfz, 3hg1, 3hig, 3hii, 3hik, 3hio, 3hit, 3hiv, 3hiw, 3hjo, 3hk1, 3hkn, 3hkq, 3hkt, 3hku, 3hkw, 3hky, 3hl5, 3hl7, 3hl8, 3hll, 3hlo, 3hmo, 3hmp, 3hp9, 3hpt, 3hqh, 3hqr, 3hrf, 3hs4, 3hs8, 3hs9, 3hu1, 3hu2, 3hu3, 3hub, 3huc, 3hv7, 3hv8, 3hvh, 3hvi, 3hvj, 3hvk, 3hww, 3hx3, 3hxi, 3hzk, 3hzm, 3hzv, 3hzy, 3i02, 3i25, 3i3b, 3i4b, 3i4y, 3i51, 3i5r, 3i5z, 3i60, 3i6c, 3i6o, 3i73, 3i7e, 3i8t, 3i9g, 3iae, 3iaf, 3iaw, 3ibi, 3ibl, 3ibn, 3ibu, 3ie3, 3ieo, 3ies, 3iet, 3if7, 3ifl, 3ifo, 3ifp, 3ig1, 3igp, 3iiw, 3iiy, 3ij0, 3ij1, 3ijg, 3ijh, 3ijy, 3ijz, 3ik1, 3ikc, 3ikd, 3ikg, 3ilq, 3imc, 3ime, 3img, 3imy, 3iny, 3io7, 3iob, 3ioc, 3iod, 3ioe, 3iof, 3iog, 3iok, 3ip5, 3ip6, 3ip8, 3ip9, 3ipa, 3iph, 3ipq, 3ips, 3ipu, 3iqg, 3iqh, 3iqi, 3iqj, 3iqq, 3iqu, 3iqv, 3isj, 3iss, 3isw, 3ith, 3iub, 3iuc, 3iue, 3iux, 3ivc, 3ivq, 3ivv, 3ivx, 3iw5, 3iw6, 3iw7, 3iw8, 3iww, 3iwy, 3jdw, 3jpx, 3jq7, 3jq8, 3jq9, 3jqa, 3jqb, 3jqf, 3jqg, 3jrs, 3jrx, 3juk, 3juo, 3jup, 3juq, 3jvk, 3jwq, 3jwr, 3jxw, 3jy0, 3jy9, 3jyj, 3jyr, 3jzg, 3jzh, 3jzj, 3jzo, 3k00, 3k02, 3k05, 3k0h, 3k0k, 3k15, 3k16, 3k1j, 3k26, 3k27, 3k2f, 3k37, 3k39, 3k3a, 3k4d, 3k4q, 3k54, 3k5i, 3k5k, 3k5x, 3k8c, 3k8d, 3k8o, 3k8q, 3k97, 3k99, 3ka2, 3kcf, 3kck, 3kd7, 3kdb, 3kdc, 3kdd, 3kdm, 3kej, 3kek, 3kgq, 3kgt, 3kgu, 3kid, 3kiv, 3kjd, 3kku, 3kmc, 3kme, 3kmm, 3kmx, 3kmy, 3kpu, 3kpv, 3kpw, 3kqb, 3kqc, 3kqd, 3kqe, 3kqm, 3kqo, 3kqp, 3kqr, 3kqs, 3kqt, 3kqw, 3kqy, 3kr0, 3kr1, 3kr2, 3kr4, 3kr5, 3ktr, 3kv2, 3kyq, 3kze, 3l08, 3l0v, 3l1n, 3l1s, 3l2y, 3l3l, 3l3m, 3l3n, 3l3q, 3l4t, 3l4u, 3l4v, 3l4w, 3l4x, 3l4y, 3l4z, 3l59, 3l5b, 3l6h, 3l6x, 3l79, 3l7a, 3l7c, 3l7d, 3l7g, 3l81, 3l8s, 3l8x, 3lbj, 3lbk, 3lbl, 3lbz, 3lc3, 3lc5, 3lcu, 3lcv, 3ldp, 3ldq, 3ldw, 3le8, 3le9, 3lea, 3lf0, 3lgl, 3lgp, 3lgs, 3lik, 3lil, 3lir, 3liw, 3ljg, 3ljj, 3ljo, 3ljz, 3lk0, 3lk1, 3lk8, 3ll8, 3llm, 3lm1, 3lmk, 3lnj, 3lnz, 3loo, 3lp4, 3lp7, 3lpf, 3lpg, 3lpi, 3lpk, 3lpl, 3lpp, 3lq2, 3lq4, 3lqi, 3lqj, 3lrh, 3luo, 3lvw, 3lxe, 3lxk, 3lxo, 3lzs, 3lzu, 3lzv, 3lzz, 3m17, 3m1k, 3m2u, 3m35, 3m36, 3m37, 3m3c, 3m3e, 3m3o, 3m3r, 3m3x, 3m3z, 3m40, 3m53, 3m54, 3m55, 3m57, 3m58, 3m59, 3m5a, 3m5e, 3m67, 3m6p, 3m6q, 3m6r, 3m89, 3m8t, 3m8u, 3m93, 3m94, 3m96, 3mag, 3mam, 3mbp, 3mct, 3mdz, 3me9, 3mea, 3met, 3meu, 3mf5, 3mfv, 3mfw, 3mg6, 3mhc, 3mhi, 3mhl, 3mhm, 3mho, 3mhw, 3mi2, 3mi3, 3miy, 3mj1, 3mjl, 3mkn, 3ml2, 3ml4, 3ml5, 3mle, 3mmf, 3mn8, 3mna, 3mo2, 3mo5, 3mo8, 3moe, 3mof, 3moh, 3mp1, 3mp6, 3ms9, 3mtw, 3muf, 3muk, 3muz, 3mv0, 3mxc, 3mxd, 3mxe, 3mxf, 3mxy, 3myq, 3mz6, 3mzc, 3n0h, 3n0n, 3n1c, 3n2c, 3n2e, 3n2p, 3n2u, 3n2v, 3n35, 3n3g, 3n3j, 3n4b, 3n5e, 3n6k, 3n75, 3n7h, 3n7o, 3n7r, 3n7s, 3n87, 3n8k, 3n8n, 3n9r, 3n9s, 3nal, 3nb5, 3nba, 3ncq, 3ncr, 3nee, 3neo, 3nes, 3new, 3nex, 3nf3, 3nfk, 3nfl, 3ng4, 3nga, 3nhi, 3nht, 3ni5, 3nii, 3nij, 3nik, 3nil, 3nim, 3nin, 3njq, 3nkk, 3nkx, 3nmq, 3nox, 3npc, 3nq3, 3nrz, 3nsn, 3nsq, 3nth, 3nti, 3nu3, 3nu4, 3nu5, 3nu6, 3nu9, 3nuj, 3nuo, 3nw3, 3nxq, 3ny3, 3nyd, 3nyn, 3nyx, 3nzk, 3o0g, 3o1d, 3o1e, 3o2m, 3o3j, 3o4k, 3o4l, 3o56, 3o5n, 3o5x, 3o64, 3o6l, 3o6m, 3o75, 3o84, 3o8p, 3o99, 3o9a, 3o9d, 3o9e, 3o9p, 3oaf, 3oap, 3oaw, 3ob1, 3ob2, 3obq, 3obu, 3obx, 3ocp, 3ocz, 3od0, 3odi, 3odl, 3odu, 3oe0, 3ogx, 3ohi, 3oik, 3oil, 3oim, 3ok9, 3oka, 3okc, 3okp, 3oku, 3okv, 3old, 3ole, 3olg, 3oli, 3omc, 3omg, 3oob, 3oq5, 3orn, 3otf, 3ouj, 3ov1, 3ove, 3ovn, 3owj, 3own, 3oxc, 3oy0, 3oy8, 3oyq, 3oys, 3oyw, 3ozg, 3ozj, 3ozp, 3ozr, 3p17, 3p2e, 3p2h, 3p2k, 3p3g, 3p3r, 3p3s, 3p3t, 3p4f, 3p4v, 3p58, 3p5l, 3p7i, 3p8h, 3p8n, 3p8o, 3p8p, 3p8z, 3p9h, 3p9l, 3p9m, 3p9t, 3pab, 3pb3, 3pb7, 3pb8, 3pb9, 3pbb, 3pcb, 3pcc, 3pce, 3pcf, 3pcg, 3pch, 3pcj, 3pck, 3pcn, 3pcu, 3pd2, 3pd3, 3pd4, 3pd8, 3pd9, 3pdh, 3pe1, 3pe2, 3pfp, 3pgl, 3pgu, 3pjg, 3pjt, 3pju, 3pkn, 3plu, 3pm1, 3pma, 3pn1, 3pn3, 3pn4, 3po1, 3po6, 3poa, 3pp7, 3ppm, 3ppo, 3ppp, 3ppq, 3ppr, 3pqz, 3prz, 3ps1, 3ps6, 3psl, 3pty, 3pwd, 3pwk, 3pwm, 3pxe, 3pxq, 3q1x, 3q2j, 3q2m, 3q43, 3q44, 3q4j, 3q4k, 3q4l, 3q5u, 3q6k, 3q6s, 3q6w, 3q6z, 3q71, 3q72, 3q7p, 3q7q, 3q8d, 3q8h, 3qaa, 3qak, 3qbc, 3qc9, 3qce, 3qcf, 3qdd, 3qel, 3qem, 3qfd, 3qfy, 3qfz, 3qgw, 3qin, 3qio, 3qip, 3qiy, 3qiz, 3qj0, 3qk0, 3qkd, 3ql9, 3qlc, 3qlm, 3qmk, 3qn7, 3qnd, 3qnj, 3qo2, 3qox, 3qps, 3qqa, 3qs4, 3qs5, 3qs6, 3qs8, 3qt6, 3qt7, 3qto, 3qtv, 3que, 3qvu, 3qvv, 3qw5, 3qwc, 3qx5, 3qx8, 3qx9, 3qxc, 3qxd, 3qxh, 3qxm, 3qxt, 3qxv, 3qyy, 3qzt, 3qzv, 3r0h, 3r0y, 3r16, 3r17, 3r1v, 3r24, 3r42, 3r4m, 3r4n, 3r4o, 3r4p, 3r5m, 3r5n, 3r5t, 3r6c, 3r6u, 3r7o, 3r8i, 3r93, 3rbm, 3rbq, 3rbu, 3rdo, 3rdq, 3rdv, 3re4, 3rf4, 3rf5, 3ri1, 3rjw, 3rl7, 3rl8, 3rlb, 3rlp, 3rlq, 3rm4, 3rm9, 3roc, 3rqe, 3rqf, 3rqg, 3rqw, 3rse, 3rsr, 3rt4, 3rt6, 3rt8, 3rtf, 3rth, 3rti, 3rtm, 3rtx, 3ru1, 3rul, 3rum, 3run, 3rux, 3rv4, 3rv6, 3rv7, 3rv8, 3rv9, 3rw9, 3rwp, 3rwq, 3rx5, 3rx7, 3rx8, 3ry8, 3ryv, 3ryw, 3ryx, 3ryy, 3ryz, 3rz0, 3rz1, 3rz5, 3rz7, 3rz8, 3rz9, 3rzi, 3s0b, 3s0d, 3s0e, 3s1g, 3s2v, 3s3v, 3s43, 3s45, 3s53, 3s54, 3s56, 3s5y, 3s6t, 3s71, 3s72, 3s73, 3s74, 3s75, 3s76, 3s77, 3s78, 3s7b, 3s7f, 3s8l, 3s8n, 3s8o, 3s8x, 3s9e, 3s9t, 3s9y, 3sap, 3sax, 3sbh, 3sbi, 3sfg, 3sh1, 3sha, 3shb, 3shc, 3shj, 3shv, 3si3, 3si4, 3sio, 3sjf, 3sk2, 3slz, 3sm0, 3sm1, 3sm2, 3smq, 3so6, 3sou, 3sow, 3spf, 3sr4, 3st5, 3st6, 3std, 3stj, 3str, 3su0, 3su1, 3su2, 3su3, 3su4, 3su5, 3su6, 3sud, 3sue, 3suf, 3sug, 3sur, 3sus, 3sut, 3suu, 3suv, 3suw, 3sv2, 3svj, 3sw2, 3sw8, 3sww, 3sx4, 3sx9, 3sxf, 3sxu, 3sym, 3szm, 3t01, 3t03, 3t08, 3t09, 3t0b, 3t0d, 3t0w, 3t0x, 3t19, 3t1a, 3t1l, 3t1m, 3t1n, 3t2p, 3t2q, 3t2v, 3t2w, 3t3c, 3t3d, 3t3e, 3t3g, 3t3h, 3t3i, 3t3u, 3t3v, 3t4n, 3t4p, 3t5i, 3t5u, 3t60, 3t64, 3t6b, 3t6j, 3t6r, 3t6y, 3t70, 3t7g, 3t82, 3t83, 3t84, 3t85, 3t8v, 3t8w, 3ta0, 3ta1, 3tam, 3tao, 3tay, 3tb6, 3tc5, 3tcg, 3tcp, 3tct, 3tcy, 3td4, 3tdh, 3tdj, 3tdu, 3te5, 3tf6, 3tfk, 3tfn, 3tfp, 3tfu, 3tfv, 3tg5, 3tgs, 3th0, 3th9, 3tif, 3tiw, 3tjh, 3tk2, 3tkw, 3tkz, 3tl0, 3tlh, 3tll, 3tmk, 3tne, 3tpx, 3ts4, 3tsd, 3tsz, 3tt4, 3ttm, 3ttn, 3ttp, 3tu1, 3tu7, 3tv5, 3tvc, 3tvw, 3tvx, 3twr, 3tws, 3twu, 3twv, 3tww, 3twx, 3tz0, 3tz2, 3tz4, 3tza, 3tzd, 3tzm, 3u0d, 3u0p, 3u0t, 3u10, 3u15, 3u2q, 3u3u, 3u3z, 3u51, 3u5l, 3u6h, 3u6i, 3u6w, 3u78, 3u7k, 3u7l, 3u7m, 3u7n, 3u7s, 3u81, 3u8j, 3u8l, 3u8m, 3u90, 3u92, 3u93, 3ual, 3uat, 3ubd, 3ucj, 3ud7, 3ud8, 3ud9, 3uda, 3udd, 3udv, 3uec, 3ued, 3uef, 3ueo, 3uf9, 3ug2, 3uib, 3uig, 3uih, 3uii, 3uij, 3uik, 3uil, 3uj9, 3ujb, 3ujc, 3ujd, 3umo, 3ump, 3umq, 3unn, 3unz, 3uo5, 3uo6, 3uod, 3uoh, 3uoj, 3upk, 3upv, 3upx, 3usx, 3ut5, 3uu1, 3uug, 3uvk, 3uvl, 3uvm, 3uvn, 3uvo, 3uvq, 3uvu, 3uvw, 3uvx, 3uw4, 3uw5, 3uw9, 3uwl, 3ux0, 3uxd, 3uxg, 3uxk, 3uxl, 3uyr, 3uz5, 3uzd, 3uzj, 3v0l, 3v0p, 3v2n, 3v2o, 3v2p, 3v2q, 3v2x, 3v30, 3v31, 3v3b, 3v3l, 3v3q, 3v43, 3v4t, 3v51, 3v5g, 3v5p, 3v5t, 3v78, 3v7c, 3v7d, 3v7t, 3v7x, 3va4, 3vbd, 3vd4, 3vd7, 3vd9, 3vdb, 3vdc, 3veh, 3vf5, 3vf7, 3vfa, 3vfb, 3vfj, 3vfq, 3vh9, 3vha, 3vhc, 3vhd, 3vhk, 3vjc, 3vje, 3vtr, 3vvy, 3vvz, 3vw0, 3vw1, 3vw2, 3vw7, 3vws, 3vx3, 3w07, 3w0l, 3w37, 3w5n, 3w9k, 3w9r, 3wb4, 3wb5, 3wdz, 3wgg, 3wha, 3wig, 3wjw, 3wmb, 3wmc, 3wp0, 3wp1, 3wq5, 3wq6, 3wqm, 3wqv, 3wqw, 3wth, 3wti, 3wtk, 3wtl, 3wtm, 3wtn, 3wto, 3wut, 3wuu, 3wuv, 3wvm, 3wz6, 3wz7, 3wzn, 3wzp, 3wzq, 3x00, 3x1k, 3zbx, 3zc5, 3zcl, 3zcw, 3zdh, 3zdv, 3zev, 3zh8, 3zhf, 3zhx, 3zhz, 3zi0, 3zi8, 3zj6, 3zk6, 3zke, 3zkf, 3zll, 3zln, 3zlr, 3zm9, 3zmm, 3zmv, 3zmz, 3zn0, 3zns, 3zos, 3zpq, 3zpr, 3zps, 3zpt, 3zpu, 3zq9, 3zqe, 3zqi, 3zrc, 3zsq, 3zsy, 3zt3, 3zt4, 3ztc, 3ztd, 3zuk, 3zv7, 3zvy, 3zxe, 3zxz, 3zy2, 3zy5, 3zyb, 3zyf, 3zyh, 3zyr, 3zyu, 3zze, 456c, 4a0j, 4a1w, 4a4c, 4a4q, 4a4v, 4a4w, 4a50, 4a51, 4a6b, 4a6c, 4a6l, 4a6s, 4a7i, 4a7j, 4a95, 4aa1, 4aa2, 4ab8, 4ab9, 4aba, 4abb, 4abd, 4abe, 4abf, 4abh, 4abi, 4abj, 4abk, 4acc, 4acd, 4acg, 4ach, 4aci, 4acm, 4ad2, 4ad3, 4ad6, 4afg, 4afh, 4ag8, 4agc, 4agd, 4agl, 4agm, 4ago, 4ah9, 4ahr, 4ahs, 4ahu, 4ai5, 4aia, 4aif, 4aj1, 4aj2, 4aj4, 4aje, 4aji, 4ajk, 4ajl, 4ajn, 4ajo, 4al4, 4alx, 4aoc, 4aoi, 4aom, 4ap7, 4aph, 4apo, 4app, 4apr, 4aq4, 4aq6, 4aqh, 4ara, 4arb, 4arw, 4as9, 4asd, 4ase, 4asj, 4att, 4au7, 4auj, 4auy, 4av0, 4av4, 4av5, 4avh, 4avi, 4avj, 4avs, 4aw8, 4ax9, 4axd, 4ayp, 4ayq, 4ayr, 4ayt, 4ayu, 4ayv, 4ayx, 4ayy, 4az2, 4az5, 4az6, 4aza, 4azb, 4azc, 4azf, 4azg, 4azi, 4b05, 4b0b, 4b0c, 4b0j, 4b1j, 4b2d, 4b2i, 4b2l, 4b32, 4b33, 4b34, 4b35, 4b3b, 4b3c, 4b3d, 4b4n, 4b4q, 4b5d, 4b5s, 4b5t, 4b5w, 4b60, 4b6o, 4b6p, 4b6q, 4b6r, 4b6s, 4b73, 4b74, 4b76, 4b7j, 4b7n, 4b7p, 4b7q, 4b7r, 4b8o, 4b8p, 4b8y, 4b9h, 4b9k, 4b9w, 4b9z, 4ba3, 4bah, 4bak, 4bam, 4ban, 4bao, 4baq, 4bb9, 4bbg, 4bbh, 4bc5, 4bch, 4bck, 4bcm, 4bcn, 4bco, 4bcp, 4bcq, 4bcs, 4bds, 4bea, 4bf1, 4bf6, 4bg6, 4bgy, 4bi6, 4bi7, 4bj8, 4bkj, 4bks, 4blb, 4bny, 4bps, 4bqg, 4bqh, 4bqs, 4bqt, 4br3, 4bs0, 4bt3, 4bt4, 4bt5, 4bt9, 4btb, 4btk, 4btm, 4btx, 4bup, 4buq, 4bvb, 4bxk, 4bzs, 4c0r, 4c16, 4c1m, 4c1t, 4c1u, 4c1w, 4c1y, 4c2v, 4c4n, 4c52, 4c5d, 4c6u, 4c6v, 4c6x, 4c6z, 4c70, 4c71, 4c72, 4c73, 4c94, 4c9w, 4c9x, 4ca4, 4ca5, 4ca6, 4ca7, 4ca8, 4cae, 4caf, 4cc2, 4cc3, 4cc5, 4cc7, 4cd0, 4cd1, 4cd4, 4cd5, 4cd6, 4cd8, 4ce1, 4ce2, 4ce3, 4ceb, 4cfl, 4cft, 4cg8, 4cg9, 4cga, 4cgi, 4cgj, 4ch2, 4ch8, 4ci1, 4ci2, 4civ, 4cix, 4ciy, 4cj4, 4cjn, 4cjp, 4cjq, 4cjr, 4ck3, 4cki, 4ckj, 4cl6, 4clj, 4cmo, 4cnh, 4cp5, 4cp7, 4cpq, 4cpr, 4cps, 4cpt, 4cpu, 4cpw, 4cpx, 4cpy, 4cpz, 4cq0, 4cr5, 4crb, 4crd, 4crf, 4crj, 4crl, 4cs9, 4csd, 4css, 4cst, 4csy, 4cu1, 4cu7, 4cu8, 4cwb, 4cwf, 4cwn, 4cwo, 4cwp, 4cwq, 4cwr, 4cws, 4cwt, 4cy1, 4czs, 4d1j, 4d1y, 4d3h, 4d4d, 4d52, 4d62, 4d7b, 4d8z, 4da5, 4daf, 4dai, 4db7, 4dbm, 4dcs, 4dcv, 4ddm, 4dds, 4ddy, 4de0, 4de5, 4de7, 4del, 4der, 4des, 4det, 4deu, 4dew, 4dfb, 4dff, 4dfg, 4dfu, 4dgb, 4dhl, 4dj7, 4djh, 4djo, 4djp, 4djq, 4djr, 4dju, 4djw, 4djx, 4djy, 4dk5, 4dko, 4dkp, 4dkq, 4dkr, 4dlj, 4dma, 4dmw, 4dn0, 4do4, 4do5, 4dow, 4dpt, 4dpu, 4dpy, 4dq2, 4dru, 4ds1, 4dst, 4dsu, 4dsy, 4dt2, 4du8, 4duh, 4dv8, 4dve, 4dx9, 4dxg, 4dy6, 4dzy, 4e0w, 4e0x, 4e1k, 4e28, 4e34, 4e35, 4e3b, 4e3g, 4e3h, 4e49, 4e4l, 4e4n, 4e5i, 4e5j, 4e67, 4e6c, 4e6d, 4e70, 4e7r, 4e81, 4e8y, 4e9c, 4e9d, 4e9u, 4ea1, 4ear, 4eb8, 4ef4, 4ef6, 4efk, 4efs, 4egk, 4ehz, 4ei4, 4ej2, 4ej8, 4ejl, 4ek9, 4eke, 4ekg, 4eki, 4el0, 4el5, 4elb, 4elf, 4elg, 4elh, 4emf, 4emr, 4en4, 4eo4, 4eo6, 4eoh, 4eoi, 4eok, 4eol, 4eon, 4eop, 4eos, 4eoy, 4ep2, 4epy, 4eqf, 4eqj, 4er1, 4er2, 4er4, 4erf, 4erq, 4ery, 4erz, 4es0, 4esg, 4etz, 4eu0, 4euc, 4euo, 4euv, 4ew2, 4ew3, 4ewh, 4ewn, 4ewr, 4exh, 4exs, 4ezo, 4ezq, 4ezr, 4ezt, 4ezw, 4ezx, 4ezy, 4ezz, 4f08, 4f0c, 4f14, 4f1l, 4f1q, 4f1s, 4f20, 4f39, 4f3i, 4f3k, 4f5y, 4f6s, 4f6u, 4f6w, 4f70, 4f7j, 4f7l, 4f7n, 4f7v, 4f8j, 4f9g, 4f9u, 4f9v, 4f9y, 4fab, 4fai, 4fak, 4fbe, 4fbx, 4fcf, 4fcm, 4fcq, 4fcr, 4fe9, 4fem, 4fev, 4few, 4ffs, 4fgt, 4fht, 4fiv, 4fjz, 4fk6, 4fk7, 4fkk, 4fl1, 4fl2, 4fl3, 4flh, 4flp, 4fm7, 4fm8, 4fmn, 4fmq, 4fmu, 4fn5, 4fnn, 4fns, 4fp1, 4fqo, 4fr3, 4frs, 4fs4, 4fse, 4fsl, 4fut, 4fvq, 4fvr, 4fxp, 4fxq, 4fxy, 4fxz, 4fys, 4fz3, 4fzj, 4g0c, 4g0k, 4g0l, 4g0p, 4g0q, 4g0y, 4g0z, 4g2r, 4g3e, 4g3f, 4g3g, 4g4p, 4g5f, 4g68, 4g69, 4g8m, 4g8n, 4g8o, 4g8r, 4g8v, 4g8y, 4g90, 4g95, 4gah, 4gbd, 4gby, 4gbz, 4ge1, 4ge4, 4ge6, 4ge7, 4ge9, 4gfd, 4gfn, 4gfo, 4gg7, 4ggz, 4ghi, 4gih, 4gii, 4giu, 4gj2, 4gj3, 4gkh, 4gki, 4glr, 4glw, 4glx, 4gly, 4gmy, 4gne, 4gnf, 4gng, 4gny, 4gpl, 4gq4, 4gq6, 4gql, 4gqp, 4gqq, 4gqr, 4gr3, 4gr8, 4gu6, 4gu9, 4gue, 4gui, 4guj, 4gv8, 4gvc, 4gvd, 4gw1, 4gw5, 4gwi, 4gwk, 4gxl, 4gy5, 4gzp, 4gzt, 4gzw, 4gzx, 4h1e, 4h3f, 4h3g, 4h3i, 4h3j, 4h3q, 4h42, 4h4b, 4h5e, 4h75, 4h7q, 4h81, 4h85, 4ha5, 4hbm, 4hbn, 4hcz, 4hdb, 4hdf, 4hdp, 4heg, 4hev, 4hf4, 4hfp, 4hfz, 4hgc, 4hiq, 4his, 4hj2, 4hla, 4hmk, 4hnc, 4hnn, 4hp0, 4hpi, 4hpy, 4hs6, 4hs8, 4hso, 4ht0, 4ht2, 4ht6, 4htp, 4hu1, 4hva, 4hvb, 4hw2, 4hw3, 4hwb, 4hwo, 4hwp, 4hwr, 4hws, 4hxw, 4hxz, 4hy1, 4hy9, 4hyb, 4hym, 4hz5, 4hzm, 4i1r, 4i2z, 4i3z, 4i47, 4i54, 4i5c, 4i67, 4i71, 4i72, 4i73, 4i74, 4i7j, 4i7k, 4i7l, 4i7m, 4i7p, 4i8n, 4i8w, 4i8x, 4i8z, 4i9h, 4i9o, 4i9u, 4iaw, 4iax, 4ib5, 4ibb, 4ibc, 4ibd, 4ibe, 4ibf, 4ibg, 4ibi, 4ibj, 4ibk, 4idn, 4ido, 4ieh, 4ifi, 4igk, 4igq, 4igr, 4igt, 4ih3, 4ih6, 4iho, 4iic, 4iid, 4iie, 4iif, 4ij1, 4ijh, 4ijl, 4ijq, 4ikn, 4in9, 4io2, 4io3, 4io4, 4io5, 4io6, 4io7, 4io8, 4ipi, 4ipj, 4ipn, 4irx, 4ish, 4isi, 4isu, 4itp, 4iue, 4iuo, 4iur, 4iut, 4iuu, 4iuv, 4iva, 4iwz, 4j03, 4j09, 4j22, 4j26, 4j2c, 4j3u, 4j44, 4j45, 4j46, 4j47, 4j48, 4j4v, 4j73, 4j74, 4j77, 4j78, 4j7d, 4j7e, 4j7i, 4j81, 4j82, 4j84, 4j86, 4j8b, 4j8g, 4j8r, 4j8t, 4j93, 4jal, 4jaz, 4jbl, 4jc1, 4jck, 4jdf, 4je7, 4je8, 4jfd, 4jfe, 4jff, 4jfi, 4jfj, 4jfk, 4jfl, 4jfm, 4jft, 4jfx, 4jfz, 4jg0, 4jg1, 4jh0, 4jhz, 4jin, 4jit, 4jiz, 4jjm, 4jjq, 4jk5, 4jk6, 4jkw, 4jls, 4jmg, 4jmh, 4jn2, 4jn4, 4jne, 4jnj, 4joe, 4jof, 4jog, 4joh, 4joj, 4jok, 4jpx, 4jpy, 4jr0, 4jsa, 4jss, 4jv6, 4jv8, 4jvb, 4jwk, 4jx9, 4jxv, 4jxw, 4jyb, 4jyc, 4jym, 4jyt, 4jyu, 4jyv, 4jz1, 4jzb, 4jzi, 4k0o, 4k0u, 4k0y, 4k10, 4k19, 4k1b, 4k1e, 4k3h, 4k3m, 4k3n, 4k3o, 4k3p, 4k3q, 4k3r, 4k42, 4k43, 4k4j, 4k55, 4k5l, 4k5m, 4k5n, 4k5o, 4k5p, 4k5y, 4k66, 4k67, 4k6i, 4k6t, 4k6u, 4k6y, 4k6z, 4k75, 4k76, 4k78, 4k7i, 4k7n, 4k7o, 4k8a, 4k8o, 4k9y, 4kab, 4kao, 4kax, 4kb9, 4kby, 4kcx, 4keq, 4kfq, 4kif, 4kij, 4kiu, 4kiw, 4kln, 4klv, 4km0, 4km2, 4kmd, 4kmz, 4kn0, 4kn1, 4kn2, 4kne, 4kni, 4knj, 4knm, 4knn, 4knz, 4ko8, 4kod, 4kom, 4kon, 4kot, 4kov, 4kow, 4kox, 4kp0, 4kp4, 4kp5, 4kp8, 4kqp, 4ks1, 4ks2, 4ks3, 4ks4, 4ks5, 4ksp, 4ksy, 4ktu, 4kup, 4kv9, 4kva, 4kvm, 4kwf, 4kwg, 4kwo, 4kww, 4kx8, 4kxb, 4kxl, 4kxm, 4kxn, 4kyh, 4kyk, 4kz3, 4kz4, 4kz7, 4kz8, 4kza, 4kzb, 4l19, 4l1u, 4l2l, 4l3o, 4l4v, 4l4z, 4l50, 4l51, 4l58, 4l6t, 4l9i, 4lar, 4lbl, 4lbo, 4lbp, 4lbu, 4lch, 4leq, 4lg6, 4lhm, 4lhv, 4lj5, 4lj8, 4ljh, 4lk6, 4lk7, 4lkd, 4lke, 4lkf, 4lkg, 4lkj, 4lkk, 4lko, 4lkq, 4lkt, 4ll3, 4llj, 4llk, 4llp, 4lm0, 4lm1, 4lm2, 4lm3, 4lm4, 4lmn, 4ln2, 4lno, 4lnp, 4lnw, 4loh, 4loi, 4loo, 4lop, 4loq, 4lov, 4loy, 4lp6, 4lpf, 4lpg, 4lph, 4lps, 4lrh, 4lrr, 4lsj, 4luo, 4luv, 4luz, 4lvt, 4lw1, 4lwc, 4lxd, 4lxz, 4ly1, 4ly9, 4lys, 4lyw, 4lzr, 4m0e, 4m0f, 4m0r, 4m12, 4m13, 4m14, 4m1d, 4m2r, 4m2u, 4m2v, 4m2w, 4m3p, 4m48, 4m5g, 4m5h, 4m5i, 4m5j, 4m5k, 4m5l, 4m5m, 4m5n, 4m6u, 4m7c, 4m7j, 4m8e, 4m8h, 4m8x, 4m8y, 4mbp, 4mc1, 4mc2, 4mc6, 4mc9, 4mdn, 4mdq, 4mdr, 4mf0, 4mf1, 4mfe, 4mg5, 4mg6, 4mg7, 4mg8, 4mg9, 4mga, 4mgb, 4mgc, 4mho, 4mhs, 4mhy, 4mhz, 4mi3, 4mi6, 4mi9, 4mic, 4mji, 4mjo, 4mjp, 4mjq, 4mjr, 4mlt, 4mlx, 4mm4, 4mm7, 4mm9, 4mmf, 4mmm, 4mmp, 4mn3, 4mnp, 4mnq, 4mnw, 4mnx, 4mny, 4mo4, 4mo8, 4mp2, 4mp7, 4mpc, 4mpe, 4mpn, 4mq6, 4mqp, 4mr3, 4mr4, 4mr5, 4mr6, 4mrd, 4mre, 4mrg, 4mrw, 4mrz, 4ms0, 4msa, 4msc, 4mse, 4msl, 4msn, 4mss, 4mue, 4muf, 4mul, 4muv, 4my6, 4myd, 4mz5, 4mz6, 4mzf, 4mzh, 4n07, 4n1z, 4n3l, 4n3w, 4n4s, 4n5d, 4n5t, 4n6g, 4n6h, 4n6y, 4n6z, 4n70, 4n7g, 4n7h, 4n7j, 4n7m, 4n7u, 4n7y, 4n84, 4n8q, 4n98, 4n99, 4n9a, 4n9b, 4n9c, 4na7, 4na8, 4na9, 4nb3, 4nbk, 4nbl, 4nbn, 4ncm, 4ncn, 4nct, 4ndu, 4ng9, 4nga, 4ngm, 4ngn, 4ngp, 4ngq, 4ngr, 4ngs, 4ngt, 4nh7, 4nh8, 4nh9, 4nj3, 4nj9, 4nja, 4nk9, 4nka, 4nks, 4nkt, 4nku, 4nl1, 4nmo, 4nmp, 4nmq, 4nmr, 4nms, 4nmt, 4nmv, 4nmx, 4nni, 4nnr, 4non, 4np2, 4np3, 4np9, 4nq6, 4nra, 4nrk, 4nrl, 4ntj, 4nuc, 4nue, 4nvp, 4nw2, 4nwc, 4nwd, 4nxq, 4nxr, 4nxu, 4nxv, 4ny3, 4nyf, 4nyi, 4nyj, 4nyt, 4nze, 4nzo, 4o04, 4o05, 4o07, 4o09, 4o0a, 4o0b, 4o0r, 4o0t, 4o0v, 4o0x, 4o0y, 4o2a, 4o2b, 4o2c, 4o2p, 4o36, 4o37, 4o3a, 4o3b, 4o3c, 4o3f, 4o3t, 4o42, 4o44, 4o45, 4o4g, 4o4y, 4o61, 4o62, 4o6w, 4o97, 4o9v, 4o9w, 4oag, 4oak, 4oc0, 4oc1, 4oc2, 4oc3, 4oc4, 4oc5, 4ock, 4ocp, 4ocq, 4oct, 4ocv, 4ocz, 4od0, 4od7, 4odk, 4odl, 4odm, 4odn, 4odp, 4odq, 4oee, 4oef, 4oeg, 4oeu, 4oew, 4oex, 4ofl, 4og3, 4og4, 4ogi, 4oi6, 4oiv, 4oks, 4oma, 4omc, 4omd, 4omj, 4omk, 4ono, 4oo9, 4or0, 4or4, 4or6, 4oru, 4orx, 4ory, 4os1, 4os2, 4os4, 4os5, 4os6, 4os7, 4otw, 4ou3, 4ouj, 4ovf, 4ovg, 4ovh, 4own, 4owo, 4owv, 4oyk, 4oys, 4oyt, 4oz1, 4ozj, 4ozl, 4ozn, 4ozo, 4p02, 4p0a, 4p0b, 4p3h, 4p4t, 4p58, 4p5d, 4p5e, 4p5z, 4p6c, 4p6w, 4p6x, 4p7s, 4pb1, 4pb2, 4pct, 4pd5, 4pd6, 4pd7, 4pd8, 4pda, 4pdk, 4pee, 4pf5, 4pft, 4pfu, 4pg3, 4pg9, 4pgb, 4pgc, 4pgd, 4pge, 4pgh, 4phu, 4pin, 4pio, 4pl6, 4pli, 4pmm, 4pms, 4pn1, 4pnu, 4pnw, 4po0, 4po7, 4poh, 4poj, 4pop, 4pov, 4pow, 4pox, 4pp0, 4pp3, 4pp5, 4pp9, 4ppa, 4ppb, 4ppc, 4pqa, 4pqn, 4pr5, 4pra, 4prb, 4prd, 4pre, 4prg, 4prh, 4pri, 4prj, 4prn, 4prp, 4ps3, 4ps7, 4ps8, 4psb, 4psh, 4psx, 4puj, 4puk, 4pul, 4pum, 4pv5, 4pvv, 4pvx, 4pvy, 4pxf, 4pxm, 4pyx, 4pyy, 4pz5, 4pzv, 4q06, 4q07, 4q08, 4q09, 4q0a, 4q0k, 4q0l, 4q18, 4q19, 4q1a, 4q1b, 4q1c, 4q1d, 4q1e, 4q1f, 4q1w, 4q1x, 4q1y, 4q3t, 4q3u, 4q46, 4q4o, 4q4p, 4q4q, 4q4r, 4q4s, 4q6d, 4q6e, 4q6f, 4q7p, 4q7s, 4q7v, 4q7w, 4q81, 4q83, 4q87, 4q8x, 4q8y, 4q90, 4q93, 4q99, 4q9m, 4q9o, 4q9y, 4qaa, 4qab, 4qb3, 4qbm, 4qc1, 4qdk, 4qem, 4qer, 4qev, 4qew, 4qf7, 4qf8, 4qf9, 4qfl, 4qfn, 4qfo, 4qfp, 4qgd, 4qge, 4qgi, 4qh7, 4qh8, 4qhc, 4qhp, 4qht, 4qij, 4qip, 4qir, 4qiy, 4qiz, 4qj0, 4qjm, 4qjo, 4qjp, 4qjr, 4qjw, 4qjx, 4qk4, 4qkd, 4ql1, 4qlk, 4qll, 4qme, 4qn9, 4qnb, 4qnu, 4qok, 4qp1, 4qp2, 4qpd, 4qpl, 4qq4, 4qqi, 4qrh, 4qsh, 4qsu, 4qsv, 4qtl, 4qtn, 4quo, 4qxo, 4qxq, 4qxr, 4qxt, 4qy3, 4qy8, 4qyo, 4qyy, 4qzs, 4r06, 4r07, 4r0a, 4r0i, 4r1e, 4r3c, 4r3s, 4r3w, 4r4c, 4r4i, 4r4o, 4r4q, 4r4t, 4r59, 4r5a, 4r5b, 4r5t, 4r5v, 4r5x, 4r6t, 4r6w, 4r6x, 4r73, 4r74, 4r75, 4r76, 4r7m, 4r8y, 4r91, 4r92, 4r93, 4r95, 4ra1, 4rab, 4rac, 4rad, 4rak, 4ran, 4rao, 4raq, 4rcg, 4rd0, 4rd3, 4rd6, 4rdn, 4re2, 4re3, 4re4, 4rfc, 4rfd, 4rfr, 4rgd, 4rh5, 4rhu, 4rhx, 4rhy, 4ris, 4riu, 4riv, 4rj4, 4rj7, 4rj8, 4rkx, 4rlt, 4rlu, 4rlw, 4rme, 4rn4, 4rn6, 4rpn, 4rpo, 4rqi, 4rqk, 4rqv, 4rqz, 4rr6, 4rra, 4rrf, 4rrg, 4rrq, 4rrr, 4rrv, 4rsk, 4rt0, 4rt1, 4rux, 4ruy, 4ruz, 4rvr, 4rwj, 4rwk, 4rwl, 4rww, 4rxh, 4rxz, 4ryd, 4ryl, 4s1g, 4sga, 4std, 4tim, 4tjz, 4tk1, 4tk3, 4tkb, 4tkh, 4tkj, 4tkn, 4tky, 4tln, 4tmk, 4tmp, 4tpk, 4tpw, 4tq3, 4tqn, 4trc, 4ts1, 4tsx, 4tsz, 4tt2, 4tte, 4tu4, 4tun, 4tv3, 4tw6, 4tw7, 4tw8, 4twt, 4txc, 4txs, 4ty1, 4ty6, 4ty8, 4ty9, 4tya, 4tyl, 4tyo, 4tz2, 4tz8, 4tzm, 4tzq, 4u0a, 4u0b, 4u0c, 4u0d, 4u0e, 4u0f, 4u0s, 4u0u, 4u0w, 4u1b, 4u2w, 4u43, 4u4x, 4u54, 4u5l, 4u5n, 4u5o, 4u5s, 4u5u, 4u5v, 4u68, 4u69, 4u6c, 4u6w, 4u6z, 4u70, 4u71, 4u73, 4u7o, 4u7q, 4u7t, 4u7v, 4u8w, 4u90, 4u91, 4ua8, 4uac, 4ual, 4uc5, 4ucc, 4ucd, 4uce, 4ud7, 4ue1, 4ufd, 4ufe, 4uff, 4ufg, 4ufh, 4ufi, 4ufj, 4ufk, 4ufl, 4ufm, 4uil, 4uin, 4uj1, 4uj2, 4uj9, 4uja, 4ujb, 4um1, 4um3, 4um9, 4uma, 4umb, 4umc, 4umn, 4und, 4unp, 4uof, 4uoh, 4up5, 4uru, 4urv, 4urw, 4urx, 4ury, 4urz, 4us3, 4us4, 4usi, 4usw, 4utn, 4utr, 4utv, 4uu5, 4uu7, 4uu8, 4uua, 4uub, 4uwl, 4ux9, 4uxq, 4uye, 4uyf, 4uyg, 4v01, 4v04, 4v05, 4v11, 4v1c, 4v24, 4v27, 4w4z, 4w50, 4w52, 4w53, 4w54, 4w55, 4w57, 4w5a, 4w5j, 4w97, 4w9d, 4w9e, 4w9f, 4w9g, 4w9j, 4w9k, 4w9o, 4w9p, 4w9w, 4w9x, 4wa9, 4wcf, 4wci, 4wef, 4wet, 4wey, 4wf2, 4wf4, 4whq, 4whr, 4whs, 4wht, 4why, 4wj7, 4wk1, 4wk2, 4wkb, 4wke, 4wkn, 4wko, 4wkp, 4wmu, 4wmv, 4wmx, 4wmy, 4wn5, 4wop, 4wov, 4wph, 4wr7, 4wr8, 4wrb, 4wri, 4wrq, 4wt2, 4wup, 4wv6, 4wvl, 4ww6, 4ww8, 4wxi, 4wy7, 4wym, 4wyp, 4wyz, 4wz6, 4x0z, 4x11, 4x13, 4x14, 4x1f, 4x1n, 4x1p, 4x1q, 4x1r, 4x1s, 4x21, 4x24, 4x34, 4x3e, 4x3h, 4x3i, 4x3k, 4x3r, 4x3s, 4x3t, 4x48, 4x50, 4x5p, 4x5q, 4x5r, 4x5y, 4x5z, 4x6h, 4x6i, 4x6m, 4x6n, 4x6o, 4x6s, 4x8n, 4x8o, 4x8p, 4x8t, 4x8u, 4x8v, 4xaq, 4xar, 4xas, 4xbo, 4xc2, 4xcb, 4xe1, 4xek, 4xgz, 4xh2, 4xhv, 4xii, 4xip, 4xiq, 4xir, 4xit, 4xj0, 4xk9, 4xkb, 4xmb, 4xmr, 4xnv, 4xnw, 4xo8, 4xoc, 4xoe, 4xqa, 4xqb, 4xrq, 4xt2, 4xtm, 4xtp, 4xtt, 4xtv, 4xtw, 4xtx, 4xty, 4xtz, 4xu0, 4xu1, 4xu2, 4xu3, 4xx3, 4xx4, 4xx9, 4xxh, 4xy8, 4xy9, 4xya, 4xyn, 4y0a, 4y16, 4y24, 4y2q, 4y38, 4y3b, 4y3j, 4y3y, 4y4j, 4y4v, 4y59, 4y5d, 4y63, 4y64, 4y79, 4y7r, 4y8d, 4y8x, 4y8y, 4y8z, 4y94, 4yb5, 4yb6, 4yb7, 4ybj, 4ybk, 4yc0, 4yc8, 4yc9, 4ycu, 4ycw, 4ydf, 4ydn, 4ye3, 4yee, 4yef, 4yes, 4ygf, 4yha, 4yhm, 4yho, 4yhp, 4yhq, 4yhz, 4yih, 4yik, 4yje, 4yjl, 4yk0, 4yk6, 4ykj, 4ykk, 4ym2, 4ymb, 4ymg, 4ymh, 4yml, 4ymq, 4ymx, 4ynb, 4ynl, 4yo8, 4yoz, 4yp1, 4yrd, 4ysi, 4ysl, 4yt6, 4yt7, 4ytc, 4ytf, 4yth, 4yti, 4yur, 4yv5, 4yw2, 4yw6, 4yw7, 4ywa, 4yx4, 4yxi, 4yxo, 4yxu, 4yy6, 4yyi, 4yym, 4yyn, 4yyt, 4yz5, 4yzu, 4z0d, 4z0e, 4z0f, 4z0k, 4z0q, 4z0u, 4z1e, 4z1j, 4z1k, 4z1n, 4z2b, 4z2o, 4z2p, 4z5w, 4z68, 4z6h, 4z6i, 4z7n, 4z7o, 4z7q, 4z83, 4z84, 4z88, 4z89, 4z8a, 4z8m, 4z93, 4zae, 4zam, 4zb6, 4zb8, 4zba, 4zbf, 4zbi, 4zcs, 4zdu, 4ze6, 4zeb, 4zec, 4zed, 4zei, 4zek, 4zfi, 4zgk, 4zhl, 4zhm, 4zij, 4zip, 4zj4, 4zj6, 4zj8, 4zjc, 4zji, 4zjj, 4zl4, 4zls, 4zme, 4zmf, 4znx, 4zo5, 4zow, 4zph, 4zqt, 4zs9, 4zsh, 4zt8, 4zud, 4zv1, 4zv2, 4zvi, 4zw3, 4zw5, 4zw6, 4zw7, 4zw8, 4zwx, 4zwy, 4zwz, 4zx0, 4zx1, 4zx3, 4zx4, 4zx5, 4zx6, 4zx8, 4zx9, 4zxx, 4zxy, 4zy0, 4zy1, 4zy2, 4zy4, 4zy5, 4zy6, 4zyf, 4zyq, 4zyt, 4zyu, 4zyx, 4zyy, 4zyz, 4zz0, 4zz1, 4zz2, 4zz3, 4zzd, 4zzx, 4zzy, 4zzz, 5a0e, 5a14, 5a2i, 5a2j, 5a2k, 5a2s, 5a3o, 5a46, 5a4c, 5a5q, 5a5v, 5a69, 5a6a, 5a6b, 5a6h, 5a6i, 5a6k, 5a6x, 5a7y, 5a81, 5a9u, 5aa8, 5aa9, 5aaa, 5aab, 5aac, 5aan, 5ab9, 5abe, 5abf, 5abg, 5abh, 5abp, 5acw, 5acx, 5acy, 5ad1, 5aei, 5ael, 5aer, 5afv, 5agu, 5agv, 5ah2, 5ahu, 5ahw, 5ai1, 5aip, 5ais, 5aiv, 5aix, 5ajc, 5ajo, 5ajp, 5alb, 5alc, 5am6, 5am7, 5amd, 5amg, 5aml, 5ant, 5anu, 5anv, 5anw, 5aoi, 5aoj, 5aol, 5aom, 5apr, 5aqf, 5aqg, 5aqh, 5aqj, 5aqk, 5aqn, 5aqo, 5aqp, 5aqq, 5aqr, 5aqt, 5aqu, 5aqv, 5aqz, 5ar0, 5arf, 5aut, 5ave, 5avf, 5avi, 5awt, 5awu, 5ayf, 5ayt, 5azf, 5azg, 5b25, 5b2d, 5b4w, 5b56, 5b5f, 5b5g, 5b6c, 5bml, 5bms, 5bnj, 5boj, 5brn, 5bry, 5brz, 5bs0, 5bs4, 5bsk, 5btv, 5btx, 5bv3, 5bw4, 5bwb, 5bwc, 5byi, 5c0m, 5c11, 5c13, 5c1m, 5c1w, 5c29, 5c2a, 5c2e, 5c2o, 5c3p, 5c4l, 5c5h, 5c5t, 5c7n, 5c85, 5c8k, 5c8m, 5c8n, 5cal, 5can, 5cao, 5cap, 5caq, 5cas, 5cau, 5cav, 5cbm, 5cbr, 5cbs, 5cc2, 5cdh, 5cep, 5ceq, 5cfa, 5cgd, 5cgv, 5chk, 5cil, 5cin, 5cj6, 5cjf, 5ckr, 5cks, 5cnj, 5cnm, 5cp5, 5cp9, 5cpr, 5cqt, 5cqu, 5cqx, 5cr7, 5cs3, 5cs6, 5csd, 5csh, 5cso, 5csp, 5cst, 5csw, 5csx, 5csz, 5ct2, 5cu2, 5cu4, 5cvd, 5cw8, 5cxa, 5cxi, 5cy9, 5cyv, 5czm, 5d0c, 5d0j, 5d0r, 5d1n, 5d1r, 5d21, 5d24, 5d25, 5d26, 5d2a, 5d2r, 5d3c, 5d3h, 5d3j, 5d3l, 5d3n, 5d3p, 5d3s, 5d3t, 5d3x, 5d45, 5d47, 5d48, 5d6j, 5d6y, 5d75, 5d7e, 5d7x, 5dah, 5dbm, 5dd0, 5dex, 5dey, 5dfp, 5dgu, 5dgw, 5dgz, 5dh4, 5dh5, 5dhg, 5dhh, 5dhj, 5dhp, 5dhq, 5dhr, 5dhs, 5dht, 5dhu, 5dia, 5dif, 5dit, 5diu, 5div, 5dk4, 5dkn, 5dkr, 5dlx, 5dlz, 5dms, 5dnu, 5doh, 5dpx, 5dq8, 5dqc, 5dqe, 5dqf, 5dro, 5drq, 5drr, 5drs, 5dtj, 5dtk, 5dts, 5dtt, 5dtw, 5du4, 5du6, 5du8, 5duc, 5dus, 5duw, 5dva, 5dw2, 5dx3, 5dx4, 5dxb, 5dxh, 5dxt, 5dxu, 5dyo, 5e0a, 5e0l, 5e0m, 5e13, 5e1b, 5e1d, 5e1m, 5e1o, 5e1s, 5e28, 5e2k, 5e2l, 5e2m, 5e2n, 5e2o, 5e2p, 5e2q, 5e2r, 5e2s, 5e2v, 5e2w, 5e3a, 5e3d, 5e4w, 5e5g, 5e6o, 5e73, 5e74, 5e7n, 5e80, 5e88, 5e89, 5e8a, 5e8f, 5e8r, 5ea3, 5ea4, 5ea6, 5ea7, 5eay, 5ech, 5eci, 5ect, 5edb, 5edc, 5edq, 5edr, 5edu, 5ee7, 5eef, 5eei, 5eek, 5eel, 5een, 5ef7, 5ef8, 5efa, 5efb, 5efc, 5efh, 5efj, 5eg4, 5egm, 5egs, 5egu, 5eh5, 5eh7, 5eh8, 5ehe, 5ehn, 5ehq, 5ehr, 5ehv, 5ehw, 5ei3, 5eie, 5eij, 5eis, 5eje, 5ejw, 5ek9, 5ekg, 5ekh, 5ekj, 5ekm, 5ekn, 5el2, 5el9, 5eld, 5elf, 5elq, 5elv, 5elw, 5em5, 5em6, 5em7, 5em8, 5em9, 5ema, 5emb, 5en3, 5eng, 5eoc, 5eok, 5eom, 5eou, 5ep7, 5epk, 5epl, 5epn, 5epp, 5epr, 5eps, 5epy, 5eq0, 5eq1, 5eqe, 5eqp, 5eqq, 5eqy, 5er1, 5er2, 5er4, 5er5, 5esq, 5eta, 5etb, 5etf, 5etj, 5etk, 5etl, 5etm, 5etn, 5eto, 5etp, 5etq, 5etr, 5ets, 5ett, 5etu, 5etv, 5etx, 5eu1, 5ev8, 5evb, 5evd, 5evk, 5evz, 5ew0, 5ewa, 5ewh, 5ewk, 5ewz, 5exl, 5exm, 5exn, 5exw, 5ey0, 5ey4, 5ey9, 5eyr, 5eyz, 5ez0, 5ezg, 5ezh, 5f08, 5f0f, 5f1c, 5f1h, 5f1j, 5f1r, 5f1v, 5f1x, 5f25, 5f2k, 5f2p, 5f2r, 5f2u, 5f3t, 5f3z, 5f41, 5f4l, 5f4p, 5f4r, 5f5z, 5f60, 5f61, 5f62, 5f63, 5f74, 5f8y, 5f90, 5f91, 5f9b, 5fb0, 5fb1, 5fb7, 5fbi, 5fck, 5fcz, 5fdc, 5fdi, 5fdo, 5fdr, 5fdz, 5fe6, 5fe7, 5fe9, 5ff6, 5fh6, 5fh7, 5fh8, 5fhn, 5fho, 5fiv, 5fjx, 5fky, 5fl0, 5fl1, 5fl4, 5fl5, 5fl6, 5flo, 5flp, 5flq, 5fls, 5flt, 5fnc, 5fnd, 5fnf, 5fng, 5fni, 5fnj, 5fnr, 5fns, 5fnt, 5fnu, 5fog, 5fol, 5fos, 5fot, 5fou, 5fov, 5fow, 5fox, 5fpi, 5fpk, 5fpp, 5fs5, 5fsc, 5fsl, 5fsn, 5fso, 5fsx, 5fsy, 5ftg, 5fto, 5fut, 5fv7, 5fwg, 5fwr, 5fyq, 5fyx, 5g17, 5g1a, 5g1n, 5g1z, 5g22, 5g2b, 5g2g, 5g43, 5g45, 5g46, 5g4m, 5g4n, 5g4o, 5g57, 5g5f, 5g5v, 5g5z, 5g60, 5g61, 5ggk, 5ggn, 5ggo, 5ggp, 5gic, 5gid, 5gj9, 5gja, 5gjd, 5glu, 5gmh, 5gmm, 5gmn, 5gmv, 5goe, 5gof, 5gp7, 5gr9, 5gs4, 5gs9, 5gsa, 5gtr, 5gu4, 5gvm, 5gvn, 5gvp, 5h08, 5h1e, 5h1t, 5h1u, 5h1v, 5h4j, 5h5f, 5h5o, 5h5q, 5h5r, 5h5s, 5h63, 5h7g, 5h85, 5h8b, 5h8e, 5h8g, 5h9p, 5h9q, 5h9r, 5h9s, 5ha1, 5hbn, 5hbs, 5hct, 5hcv, 5hcx, 5hcy, 5hd0, 5hda, 5hdu, 5hdv, 5hdx, 5hdz, 5he4, 5he5, 5he7, 5heb, 5hed, 5hes, 5hey, 5hf1, 5hfb, 5hfc, 5hff, 5hgc, 5hhx, 5hi7, 5hip, 5his, 5hjb, 5hjc, 5hjd, 5hjq, 5hk1, 5hkh, 5hpm, 5hrv, 5hrw, 5hrx, 5htb, 5htc, 5htz, 5hu0, 5hu1, 5hu9, 5huw, 5huy, 5hv0, 5hva, 5hvs, 5hvt, 5hvu, 5hwu, 5hwv, 5hyq, 5hyr, 5hyx, 5hz5, 5hz6, 5hz8, 5hz9, 5i1q, 5i25, 5i29, 5i2e, 5i2f, 5i38, 5i3a, 5i3v, 5i3w, 5i3x, 5i3y, 5i4v, 5i7x, 5i7y, 5i80, 5i88, 5i8c, 5i8g, 5i8p, 5i9i, 5i9x, 5i9y, 5i9z, 5ia0, 5ia1, 5ia2, 5ia3, 5ia4, 5ia5, 5icx, 5icy, 5icz, 5id0, 5id1, 5ie1, 5iez, 5if6, 5igk, 5igl, 5igm, 5ih2, 5ih9, 5ihh, 5ii1, 5ii2, 5ij7, 5ijj, 5ijp, 5ijr, 5ikb, 5il1, 5ime, 5in9, 5iok, 5iop, 5ioy, 5ioz, 5ipa, 5ipc, 5ipj, 5iq6, 5iql, 5irr, 5isl, 5isz, 5itf, 5ito, 5itp, 5iu4, 5iu6, 5iu7, 5iu8, 5iua, 5iub, 5iug, 5ivc, 5ive, 5ivj, 5ivn, 5ivv, 5ivy, 5iw0, 5iwg, 5ix0, 5ix1, 5ixq, 5ixt, 5iy4, 5iyv, 5iyy, 5izf, 5izj, 5izl, 5izu, 5j0d, 5j19, 5j1r, 5j1x, 5j20, 5j27, 5j2x, 5j32, 5j3l, 5j41, 5j4n, 5j5r, 5j5x, 5j64, 5j6a, 5j6l, 5j6m, 5j6n, 5j71, 5j74, 5j7q, 5j7w, 5j82, 5j86, 5j8m, 5j8u, 5j8z, 5j9f, 5j9x, 5ja0, 5jal, 5jan, 5jcb, 5jek, 5jeo, 5jf1, 5jf2, 5jf3, 5jf4, 5jf5, 5jf6, 5jf7, 5jf8, 5jfp, 5jfu, 5jg1, 5jgi, 5jgq, 5jha, 5jhb, 5jhd, 5jhk, 5ji8, 5jjm, 5jm4, 5jop, 5jox, 5jq5, 5jq7, 5jqb, 5jr2, 5js3, 5jsg, 5jsj, 5jsq, 5jss, 5jt9, 5jur, 5jvi, 5jxn, 5jxq, 5jy0, 5jy3, 5jzi, 5k03, 5k0h, 5k0m, 5k13, 5k1d, 5k1f, 5k48, 5k4z, 5k51, 5k6s, 5k7h, 5k8o, 5k8s, 5k9w, 5ka1, 5ka3, 5ka7, 5ka9, 5kab, 5kad, 5kam, 5kap, 5kau, 5kax, 5kbe, 5kbf, 5kbg, 5kbh, 5kbi, 5kby, 5kcb, 5kdr, 5kej, 5kew, 5kez, 5kh3, 5kh7, 5khg, 5khh, 5khi, 5khj, 5khk, 5khm, 5klp, 5klr, 5klt, 5kly, 5klz, 5km0, 5km1, 5km2, 5km3, 5km5, 5km9, 5kma, 5knj, 5knt, 5ko1, 5ko5, 5kqx, 5kqy, 5kr0, 5kr1, 5kr2, 5ks7, 5kva, 5kw2, 5kww, 5kxc, 5kya, 5kyj, 5kz0, 5kzp, 5kzq, 5l0h, 5l2i, 5l2s, 5l2t, 5l2w, 5l2y, 5l2z, 5l30, 5l3a, 5l3e, 5l3j, 5l4f, 5l4i, 5l4j, 5l4m, 5l7e, 5l7f, 5l7g, 5l7h, 5l7k, 5l87, 5l8a, 5l8c, 5l8n, 5l8o, 5l8y, 5l9g, 5l9i, 5l9l, 5l9o, 5laq, 5lb7, 5ld8, 5ldk, 5ldm, 5ldo, 5ldp, 5lif, 5ljj, 5ljq, 5ljt, 5ll4, 5ll5, 5ll9, 5lla, 5llc, 5lle, 5llg, 5llh, 5lli, 5llo, 5llp, 5lne, 5lny, 5lom, 5lp6, 5lpj, 5lpk, 5lpl, 5lpm, 5lsg, 5lsh, 5lso, 5ltn, 5lu2, 5lud, 5luu, 5lvd, 5lvl, 5lvn, 5lvq, 5lvr, 5lwd, 5lwe, 5lwm, 5lws, 5lxb, 5ly1, 5ly3, 5lyn, 5lyr, 5lz4, 5lz5, 5lz7, 5m04, 5m0m, 5m17, 5m1z, 5m23, 5m25, 5m28, 5m44, 5m4c, 5m4f, 5m4i, 5m4k, 5m4q, 5m4u, 5m56, 5m5d, 5m63, 5m77, 5m7s, 5m7t, 5m7u, 5m9w, 5ma7, 5mav, 5may, 5mb1, 5mby, 5meh, 5mek, 5mes, 5mev, 5mf6, 5mfr, 5mfs, 5mft, 5mg2, 5mge, 5mgf, 5mgj, 5mgk, 5mgx, 5mhp, 5mi3, 5mi5, 5mi6, 5mi7, 5mim, 5mk1, 5mk3, 5mk9, 5mka, 5mkr, 5mks, 5ml2, 5ml3, 5ml6, 5ml8, 5mlj, 5mlo, 5mlw, 5mme, 5mmg, 5mn1, 5mnb, 5mng, 5mnh, 5mnn, 5mno, 5mnr, 5mnx, 5mo0, 5mo2, 5mo8, 5mob, 5moc, 5mod, 5moe, 5mon, 5moq, 5mos, 5mpk, 5mpn, 5mpz, 5mqe, 5mrb, 5mrm, 5mro, 5mrp, 5msb, 5mte, 5mtv, 5mtx, 5mty, 5muc, 5mw4, 5mwh, 5mwj, 5mwp, 5mwy, 5mwz, 5mxf, 5mxo, 5my8, 5myg, 5myk, 5myo, 5myx, 5mz8, 5n0d, 5n0e, 5n0f, 5n16, 5n17, 5n18, 5n1p, 5n1r, 5n1s, 5n1x, 5n1z, 5n21, 5n24, 5n25, 5n2t, 5n2x, 5n2z, 5n31, 5n34, 5n3v, 5n3y, 5n53, 5n55, 5n58, 5n69, 5n6s, 5n70, 5n7g, 5n7v, 5n7x, 5n84, 5n87, 5n8b, 5n8e, 5n8j, 5n8t, 5n8v, 5n8w, 5n93, 5n99, 5n9k, 5n9l, 5n9n, 5n9r, 5n9s, 5na0, 5nad, 5nai, 5nap, 5nau, 5nbw, 5ndd, 5ndf, 5ne5, 5nea, 5neb, 5nee, 5nf5, 5nf6, 5nf9, 5nfa, 5nfb, 5nfh, 5ng9, 5nge, 5ngz, 5nih, 5nin, 5niy, 5njx, 5njz, 5nk2, 5nk3, 5nk4, 5nk6, 5nk7, 5nk8, 5nk9, 5nka, 5nkb, 5nkc, 5nkd, 5nkg, 5nkh, 5nki, 5nkk, 5nkn, 5nlk, 5nme, 5nmf, 5nmg, 5nn0, 5nn5, 5nn6, 5nne, 5npr, 5nps, 5ntt, 5nu3, 5nu5, 5nvv, 5nvw, 5nvx, 5nvy, 5nvz, 5nw0, 5nw1, 5nw2, 5nw7, 5nwe, 5nwi, 5nxg, 5nxi, 5nxo, 5nxp, 5nxq, 5nxv, 5nxw, 5ny1, 5ny3, 5ny6, 5nya, 5nyh, 5nyz, 5nz2, 5nz4, 5nze, 5nzf, 5nzn, 5nzo, 5nzp, 5nzq, 5o07, 5o0a, 5o0b, 5o0j, 5o1a, 5o1b, 5o1c, 5o1d, 5o1e, 5o1f, 5o1g, 5o1h, 5o1i, 5o22, 5o2d, 5o3q, 5o3r, 5o4f, 5o4z, 5o55, 5o58, 5o5a, 5o9o, 5o9p, 5o9q, 5o9r, 5o9y, 5oa2, 5oa6, 5oah, 5oax, 5obj, 5obr, 5oci, 5odx, 5ody, 5oei, 5ofi, 5ofu, 5ofw, 5ogl, 5oh1, 5oh2, 5oh3, 5oh4, 5oh7, 5oh9, 5oha, 5oje, 5oku, 5olk, 5om2, 5om3, 5om7, 5oot, 5op2, 5op4, 5op5, 5opb, 5opr, 5ops, 5opu, 5opv, 5oq4, 5oq5, 5oq6, 5oq7, 5oq8, 5oqu, 5org, 5orh, 5orj, 5ork, 5orl, 5orr, 5ort, 5orv, 5orw, 5ory, 5orz, 5os0, 5os1, 5os2, 5os3, 5os4, 5os5, 5os7, 5os8, 5osd, 5ose, 5osk, 5osl, 5oss, 5ost, 5ot8, 5ot9, 5ota, 5otc, 5otr, 5otz, 5ou1, 5ou2, 5ou3, 5oug, 5ouh, 5ov8, 5ovc, 5ovp, 5ovr, 5ovv, 5ovx, 5ow1, 5owa, 5owf, 5owh, 5owl, 5oxk, 5oxl, 5q0d, 5q0e, 5q0f, 5q0g, 5q0h, 5qa4, 5qa5, 5qa6, 5qa7, 5qa8, 5qa9, 5qaa, 5qab, 5qac, 5qad, 5qae, 5qaf, 5qag, 5qah, 5qai, 5qaj, 5qak, 5qal, 5qam, 5qan, 5qao, 5qap, 5qaq, 5qar, 5qas, 5qat, 5qau, 5qav, 5qaw, 5qax, 5qay, 5qaz, 5qb0, 5qb1, 5qb2, 5qb3, 5qck, 5qcl, 5qcm, 5qcn, 5std, 5sve, 5svi, 5svk, 5svl, 5svx, 5svy, 5svz, 5swf, 5sxm, 5sym, 5syn, 5sz0, 5sz1, 5sz2, 5sz3, 5sz4, 5sz5, 5sz6, 5sz7, 5t18, 5t19, 5t1i, 5t1k, 5t1l, 5t1m, 5t2t, 5t35, 5t52, 5t54, 5t5g, 5t6j, 5t6z, 5t70, 5t78, 5t7s, 5t8o, 5t8p, 5t8q, 5t8r, 5t9u, 5t9w, 5t9z, 5ta2, 5ta4, 5tb6, 5tbe, 5tbm, 5tbp, 5tci, 5tcj, 5tco, 5tcy, 5tdb, 5tdr, 5tdw, 5tef, 5teg, 5tfx, 5th2, 5th4, 5tha, 5thi, 5thj, 5thn, 5ti0, 5tkj, 5tkk, 5tks, 5tkt, 5tku, 5tln, 5tmp, 5tp0, 5tpb, 5tpc, 5tpx, 5tq1, 5tqe, 5tqg, 5tqs, 5trf, 5tt3, 5tt8, 5ttf, 5ttg, 5ttw, 5tuo, 5tus, 5tuy, 5tuz, 5tv1, 5tv3, 5tvn, 5tw2, 5tw5, 5twg, 5twh, 5twj, 5two, 5txy, 5ty1, 5ty8, 5ty9, 5tya, 5tzd, 5tzf, 5tzg, 5tzo, 5u0d, 5u0e, 5u0f, 5u0g, 5u0w, 5u0z, 5u11, 5u12, 5u13, 5u14, 5u28, 5u2j, 5u49, 5u4b, 5u4d, 5u4x, 5u5l, 5u69, 5u6b, 5u6d, 5u6j, 5u6k, 5u8a, 5u8f, 5u98, 5u9i, 5uc1, 5uc4, 5uch, 5uci, 5ucj, 5ueu, 5uex, 5uey, 5uez, 5uf0, 5ufc, 5uff, 5ufi, 5ufo, 5ufp, 5ufr, 5ufs, 5uga, 5ugb, 5ugh, 5ujv, 5uk8, 5ukj, 5ul1, 5ul6, 5ula, 5uln, 5ulp, 5ult, 5umw, 5umx, 5umy, 5umz, 5unf, 5ung, 5unh, 5unj, 5unp, 5uoo, 5uov, 5upe, 5upf, 5upj, 5upz, 5ur9, 5ura, 5usy, 5usz, 5ut0, 5ut4, 5ut6, 5uv1, 5uv2, 5uv5, 5uw5, 5uwi, 5uwj, 5uwk, 5uwp, 5uxf, 5uxm, 5uxn, 5uzk, 5v0n, 5v13, 5v1y, 5v2l, 5v2p, 5v2q, 5v35, 5v3r, 5v4b, 5v5y, 5v6u, 5v6y, 5v79, 5v7a, 5v82, 5v8h, 5v8j, 5v8p, 5vad, 5var, 5vb5, 5vb6, 5vb7, 5vb9, 5vc3, 5vc4, 5vc5, 5vc6, 5vcv, 5vcw, 5vcx, 5vcy, 5vcz, 5vd0, 5vd1, 5vd2, 5vd3, 5vdk, 5vdp, 5vdt, 5vfj, 5vfm, 5vgy, 5vi6, 5vih, 5vii, 5vij, 5vja, 5vjn, 5vjp, 5vkc, 5vkm, 5vl2, 5vlh, 5vll, 5vm0, 5vnb, 5vo1, 5vo2, 5voj, 5vp9, 5vqi, 5vr8, 5vs6, 5vsb, 5vsc, 5vsd, 5vse, 5vsf, 5vsj, 5vwi, 5vwk, 5vyy, 5vzu, 5vzy, 5w10, 5w1e, 5w3i, 5w44, 5w4r, 5w4v, 5w5s, 5w5u, 5w6r, 5w6t, 5w6u, 5w73, 5w7i, 5w7j, 5w7u, 5w7x, 5w8j, 5w92, 5w99, 5w9g, 5wa1, 5wa4, 5wa7, 5wa8, 5wa9, 5wal, 5wap, 5wb3, 5wb6, 5wbm, 5wbo, 5wbp, 5wcm, 5wdc, 5wdw, 5we9, 5web, 5wef, 5wei, 5wev, 5wex, 5wf3, 5wf5, 5wf6, 5wfm, 5wfw, 5wg9, 5wgp, 5wic, 5wii, 5wio, 5wip, 5wir, 5wj6, 5wkf, 5wl0, 5wle, 5wlo, 5wlt, 5wlv, 5wmt, 5wou, 5wp5, 5wqc, 5wqd, 5wqj, 5wqk, 5wrs, 5ws3, 5wtt, 5wuk, 5wxf, 5wxg, 5wxh, 5wxo, 5wxp, 5wyx, 5wyz, 5wzr, 5x13, 5x4m, 5x4n, 5x4o, 5x4p, 5x4q, 5x54, 5x62, 5x72, 5x73, 5x74, 5x9o, 5xg5, 5xhs, 5xhz, 5xiw, 5xmx, 5xn3, 5xo2, 5xo7, 5xof, 5xpi, 5xs8, 5xsr, 5xsu, 5xup, 5xva, 5xvf, 5xvg, 5xwr, 5xxk, 5xyf, 5y0f, 5y0g, 5y0x, 5y12, 5y13, 5y1u, 5y20, 5y24, 5y2f, 5y59, 5y6k, 5y7w, 5y7z, 5y80, 5y8y, 5y94, 5ya5, 5yas, 5yba, 5ybe, 5ybi, 5yc1, 5yc2, 5yc3, 5yc4, 5yc8, 5yco, 5yf1, 5yfs, 5yft, 5ygd, 5ygf, 5yh8, 5yhe, 5yhg, 5yia, 5yie, 5yj8, 5yjb, 5yjm, 5yl2, 5ylj, 5yls, 5ylu, 5ylv, 5ypo, 5yqw, 5yto, 5ytu, 5yv5, 5yvt, 5yvx, 5ywx, 5yy4, 5yyb, 5yyf, 5yyz, 5yz2, 5z1c, 5z5f, 5z68, 5z7b, 5z7j, 5z89, 5z95, 5z99, 5za7, 5za8, 5za9, 5zae, 5zaf, 5zag, 5zah, 5zaj, 5zc5, 5zia, 5zk3, 5zk8, 5zkb, 5zkc, 5zku, 5zla, 5znp, 5zo8, 5zo9, 5zoo, 5zu0, 5zuj, 5zzw, 6a6w, 6abp, 6afa, 6afh, 6agg, 6am8, 6ami, 6an1, 6aol, 6aom, 6ap6, 6ap7, 6ap8, 6aps, 6apt, 6apu, 6apv, 6aqf, 6aqo, 6aqq, 6aqs, 6ar2, 6ar9, 6arj, 6asz, 6at0, 6atv, 6au5, 6axj, 6axk, 6axl, 6axp, 6ayh, 6ayi, 6ayn, 6ayo, 6ayq, 6ayr, 6azk, 6azl, 6b16, 6b1c, 6b1k, 6b27, 6b2c, 6b2q, 6b3v, 6b4d, 6b4l, 6b4n, 6b4u, 6b59, 6b5a, 6b5i, 6b5o, 6b5q, 6b5r, 6b5t, 6b67, 6b7a, 6b7b, 6b7c, 6b7d, 6b7e, 6b7f, 6b7h, 6b8y, 6b95, 6b96, 6b97, 6b98, 6baw, 6bbs, 6bc9, 6bdy, 6beb, 6bec, 6bed, 6beh, 6bgu, 6bgv, 6bgw, 6bgx, 6bgy, 6bgz, 6bh0, 6bh1, 6bh2, 6bh5, 6bhd, 6bhe, 6bhh, 6bhi, 6bhv, 6bj3, 6bm5, 6bm6, 6bnl, 6bqg, 6bqh, 6bt6, 6bu0, 6bu3, 6buu, 6bvb, 6bw2, 6c0s, 6c1s, 6c28, 6c3e, 6c4d, 6c4u, 6c5f, 6c7q, 6c7w, 6c7x, 6c91, 6c99, 6cb5, 6cbf, 6cbg, 6cbh, 6cct, 6ccu, 6cd8, 6cd9, 6cdc, 6cdg, 6cdj, 6cdl, 6cdm, 6cdo, 6cdp, 6ce2, 6ce6, 6ced, 6cf6, 6cfc, 6cfd, 6cgt, 6chh, 6chl, 6chn, 6chp, 6cjv, 6ckc, 6ckr, 6cks, 6ckw, 6clv, 6cms, 6cn5, 6coj, 6cpa, 6cpw, 6csp, 6csq, 6csr, 6css, 6ct7, 6cve, 6cvf, 6cvv, 6cvw, 6cvx, 6cvy, 6cwf, 6cwh, 6cwi, 6cwn, 6czb, 6czc, 6czd, 6cze, 6czi, 6d1u, 6d28, 6d2o, 6d50, 6d55, 6d56, 6d5e, 6d5g, 6d5h, 6d5j, 6d9x, 6dai, 6dak, 6dar, 6das, 6df1, 6df2, 6dh0, 6dh1, 6dh2, 6dh6, 6dh7, 6dh8, 6dhc, 6dif, 6dik, 6dil, 6dj1, 6dj2, 6dj5, 6dj7, 6djc, 6dkb, 6dkg, 6dki, 6dm8, 6dne, 6dq4, 6dq5, 6dq6, 6dq8, 6drt, 6dry, 6dub, 6dxl, 6e2o, 6e49, 6e4a, 6e7j, 6e8k, 6e8m, 6e9a, 6ea1, 6ea2, 6eaa, 6eab, 6ebe, 6ecz, 6eda, 6edr, 6ee2, 6ee3, 6ee4, 6ee6, 6eea, 6eed, 6eee, 6eeh, 6eeo, 6egs, 6ei5, 6eif, 6eij, 6eip, 6eiq, 6eir, 6eis, 6eiz, 6ej2, 6ej3, 6ej4, 6ekq, 6el5, 6eln, 6elo, 6elp, 6emu, 6en5, 6en6, 6eo8, 6eo9, 6eog, 6eol, 6ep4, 6epa, 6epy, 6epz, 6eq1, 6eq8, 6eqp, 6equ, 6eqv, 6eqw, 6eqx, 6er3, 6er4, 6esj, 6et8, 6euw, 6eux, 6evn, 6evo, 6evp, 6evr, 6eww, 6ex1, 6exi, 6exj, 6exs, 6ey8, 6ey9, 6eya, 6eyb, 6eyt, 6ezi, 6ezq, 6f05, 6f08, 6f09, 6f1j, 6f1n, 6f20, 6f28, 6f29, 6f3b, 6f3f, 6f4w, 6f4x, 6f5u, 6f5w, 6f6i, 6f6n, 6f6s, 6f7q, 6f7t, 6f8g, 6f90, 6f92, 6f9g, 6f9r, 6f9t, 6f9u, 6f9v, 6fa2, 6fa3, 6fa4, 6fa5, 6faa, 6fac, 6faf, 6fam, 6fap, 6fau, 6fav, 6faw, 6fba, 6fbw, 6fby, 6fc6, 6fcl, 6fe0, 6fe1, 6fgf, 6fgg, 6fhk, 6fhq, 6fhu, 6fi1, 6fi4, 6fi5, 6fii, 6fiv, 6fjf, 6fjm, 6fky, 6flg, 6fmc, 6fmf, 6fmi, 6fmj, 6fmk, 6fnf, 6fng, 6fni, 6fnj, 6fnq, 6fnr, 6fns, 6fnt, 6fo5, 6fs0, 6fs1, 6fsd, 6fse, 6ftp, 6ftz, 6fu4, 6fug, 6fuh, 6fui, 6fuj, 6fut, 6fv4, 6fx1, 6fyz, 6g0q, 6g0z, 6g14, 6g15, 6g22, 6g2l, 6g2m, 6g2n, 6g34, 6g35, 6g36, 6g37, 6g38, 6g39, 6g3a, 6g3o, 6g3q, 6g3v, 6g46, 6g47, 6g4y, 6g4z, 6g6y, 6g6z, 6g84, 6g85, 6g86, 6g98, 6g9b, 6g9i, 6g9u, 6gbx, 6ge7, 6gf9, 6gfs, 6gfx, 6gfy, 6gfz, 6gg4, 6ggv, 6ghh, 6ghj, 6ghp, 6gji, 6gjj, 6gjl, 6gjm, 6gjn, 6gjr, 6gl8, 6gl9, 6gla, 6glb, 6gmq, 6gmx, 6gnm, 6gnp, 6gnr, 6gnw, 6gon, 6goo, 6got, 6gpb, 6gr7, 6grp, 6gu2, 6gu3, 6gu6, 6gu7, 6gub, 6guc, 6gue, 6guf, 6guh, 6guk, 6gvz, 6gw1, 6gw4, 6gwr, 6gzd, 6gzl, 6gzm, 6h0b, 6h1u, 6h29, 6h2t, 6h2z, 6h33, 6h34, 6h36, 6h37, 6h38, 6h41, 6h5w, 6h5x, 6h7b, 6h7j, 6h7k, 6h7l, 6h7m, 6h7n, 6h7o, 6h8s, 6h96, 6hai, 6hd6, 6hgz, 6hh3, 6hh5, 6hke, 6hlx, 6hly, 6hlz, 6hm2, 6hm4, 6hmg, 6hoy, 6hpg, 6hpw, 6hqy, 6hrq, 6hsh, 6ht1, 6htg, 6iiu, 6iiv, 6m9t, 6ma1, 6ma2, 6ma3, 6ma4, 6ma5, 6mi6, 6mil, 6mim, 6min, 6miq, 6mj7, 6mjf, 6mnf, 6msy, 6mu3, 6mub, 6mvu, 6mwe, 6mxe, 6q3q, 6rnt, 6std, 6tim, 6upj, 7abp, 7gpb, 7hvp, 7kme, 7std, 7upj, 830c, 8a3h, 8abp, 8cpa, 8gpb, 966c, 9abp, 9hvp, 9icd |
| --- |

Test set

| 1a30, 1bcu, 1bzc, 1c5z, 1e66, 1eby, 1g2k, 1gpk, 1gpn, 1h22, 1h23, 1k1i, 1lpg, 1mq6, 1nc1, 1nc3, 1nvq, 1o0h, 1o3f, 1o5b, 1owh, 1oyt, 1p1n, 1p1q, 1ps3, 1pxn, 1q8t, 1q8u, 1qf1, 1qkt, 1r5y, 1s38, 1sqa, 1syi, 1u1b, 1uto, 1vso, 1w4o, 1y6r, 1yc1, 1ydr, 1ydt, 1z6e, 1z95, 1z9g, 2al5, 2br1, 2brb, 2c3i, 2cbv, 2cet, 2fvd, 2fxs, 2hb1, 2iwx, 2j78, 2j7h, 2p15, 2p4y, 2pog, 2qbp, 2qbq, 2qbr, 2qe4, 2qnq, 2r9w, 2v00, 2v7a, 2vkm, 2vvn, 2vw5, 2w4x, 2w66, 2wbg, 2wca, 2weg, 2wer, 2wn9, 2wnc, 2wtv, 2wvt, 2x00, 2xb8, 2xbv, 2xdl, 2xii, 2xj7, 2xnb, 2xys, 2y5h, 2yfe, 2yge, 2yki, 2ymd, 2zb1, 2zcq, 2zcr, 2zda, 2zy1, 3acw, 3ag9, 3ao4, 3arp, 3arq, 3aru, 3arv, 3ary, 3b1m, 3b27, 3b5r, 3b65, 3b68, 3bgz, 3bv9, 3cj4, 3coy, 3coz, 3d4z, 3d6q, 3dd0, 3dx1, 3dx2, 3dxg, 3e5a, 3e92, 3e93, 3ebp, 3ehy, 3ejr, 3f3a, 3f3c, 3f3d, 3f3e, 3fcq, 3fur, 3fv1, 3fv2, 3g0w, 3g2n, 3g2z, 3g31, 3gbb, 3gc5, 3ge7, 3gnw, 3gr2, 3gv9, 3gy4, 3ivg, 3jvr, 3jvs, 3jya, 3k5v, 3kgp, 3kr8, 3kwa, 3l7b, 3lka, 3mss, 3myg, 3n76, 3n7a, 3n86, 3nq9, 3nw9, 3nx7, 3o9i, 3oe4, 3oe5, 3ozs, 3ozt, 3p5o, 3prs, 3pww, 3pxf, 3pyy, 3qgy, 3qqs, 3r88, 3rlr, 3rr4, 3rsx, 3ryj, 3syr, 3tsk, 3twp, 3u5j, 3u8k, 3u8n, 3u9q, 3udh, 3ueu, 3uev, 3uew, 3uex, 3ui7, 3uo4, 3up2, 3uri, 3utu, 3uuo, 3wtj, 3wz8, 3zdg, 3zso, 3zsx, 3zt2, 4abg, 4agn, 4agp, 4agq, 4bkt, 4cig, 4ciw, 4cr9, 4cra, 4crc, 4ddh, 4ddk, 4de1, 4de2, 4de3, 4djv, 4dld, 4dli, 4e5w, 4e6q, 4ea2, 4eky, 4eo8, 4eor, 4f09, 4f2w, 4f3c, 4f9w, 4gfm, 4gid, 4gkm, 4gr0, 4hge, 4ih5, 4ih7, 4ivb, 4ivc, 4ivd, 4j21, 4j28, 4j3l, 4jfs, 4jia, 4jsz, 4jxs, 4k18, 4k77, 4kz6, 4kzq, 4kzu, 4llx, 4lzs, 4m0y, 4m0z, 4mgd, 4mme, 4ogj, 4owm, 4pcs, 4qac, 4qd6, 4rfm, 4tmn, 4twp, 4ty7, 4u4s, 4w9c, 4w9h, 4w9i, 4w9l, 4wiv, 4x6p, 5a7b, 5aba, 5c28, 5c2h, 5dwr, 5tmn |
| --- |

# Table S3. RDkit ligand descriptors used for training along with multi-shelled ECIF and weighted ECIF features.

| MaxEStateIndex, MinEStateIndex, MaxAbsEStateIndex, MinAbsEStateIndex, qed, MolWt, HeavyAtomMolWt, ExactMolWt, NumValenceElectrons, FpDensityMorgan1, FpDensityMorgan2, FpDensityMorgan3, BalabanJ, BertzCT, Chi0, Chi0n, Chi0v, Chi1, Chi1n, Chi1v, Chi2n, Chi2v, Chi3n, Chi3v, Chi4n, Chi4v, HallKierAlpha, Kappa1, Kappa2, Kappa3, LabuteASA, PEOE_VSA14, SMR_VSA1, SMR_VSA10, SMR_VSA2, SMR_VSA3, SMR_VSA4, SMR_VSA5, SMR_VSA6, SMR_VSA7, SMR_VSA9, SlogP_VSA1, SlogP_VSA10, SlogP_VSA11, SlogP_VSA12, SlogP_VSA2, SlogP_VSA3, SlogP_VSA4, SlogP_VSA5, SlogP_VSA6, SlogP_VSA7, SlogP_VSA8, TPSA, EState_VSA1, EState_VSA10, EState_VSA11, EState_VSA2, EState_VSA3, EState_VSA4, EState_VSA5, EState_VSA6, EState_VSA7, EState_VSA8, EState_VSA9, VSA_EState1, VSA_EState10, VSA_EState2, VSA_EState3, VSA_EState4, VSA_EState5, VSA_EState6, VSA_EState7, VSA_EState8, VSA_EState9, FractionCSP3, HeavyAtomCount, NHOHCount, NOCount, NumAliphaticCarbocycles, NumAliphaticHeterocycles, NumAliphaticRings, NumAromaticCarbocycles, NumAromaticHeterocycles, NumAromaticRings, NumHAcceptors, NumHDonors, NumHeteroatoms, NumRotatableBonds, NumSaturatedCarbocycles, NumSaturatedHeterocycles, NumSaturatedRings, RingCount, MolLogP, MolMR, fr_Al_COO, fr_Al_OH, fr_Al_OH_noTert, fr_ArN, fr_Ar_N, fr_Ar_NH, fr_Ar_OH, fr_COO, fr_COO2, fr_C_O, fr_C_O_noCOO, fr_C_S, fr_HOCCN, fr_Imine, fr_NH0, fr_NH1, fr_NH2, fr_N_O, fr_Ndealkylation1, fr_Ndealkylation2, fr_Nhpyrrole, fr_SH, fr_aldehyde, fr_alkyl_carbamate, fr_alkyl_halide, fr_allylic_oxid, fr_amide, fr_amidine, fr_aniline, fr_aryl_methyl, fr_azo, fr_barbitur, fr_benzene, fr_bicyclic, fr_dihydropyridine, fr_epoxide, fr_ester, fr_ether, fr_furan, fr_guanido, fr_halogen, fr_hdrzine, fr_hdrzone, fr_imidazole, fr_imide, fr_isocyan, fr_isothiocyan, fr_ketone, fr_ketone_Topliss, fr_lactam, fr_lactone, fr_methoxy, fr_morpholine, fr_nitrile, fr_nitro, fr_nitro_arom, fr_nitroso, fr_oxazole, fr_oxime, fr_para_hydroxylation, fr_phenol, fr_phenol_noOrthoHbond, fr_piperdine, fr_piperzine, fr_priamide, fr_pyridine, fr_quatN, fr_sulfide, fr_sulfonamd, fr_sulfone, fr_term_acetylene, fr_tetrazole, fr_thiazole, fr_thiocyan, fr_thiophene, fr_urea |
| --- |

# Table S4. Evaluation result of other CASF datasets.

Those with the notation "::LD" include the ligand descriptor as input. Orhobor et al report the results of several distance hyperparameters for PDECIF and ECIF, so only the best from each dataset is cited. For each dataset, the best Pearson's R and RMSE for "+ ligand" and non "+ ligand" each are marked in bold.

| dataset | representation | R | RMSE |
| --- | --- | --- | --- |
| CASF-2007 | ECIF | 0.812 | 1.472 |
| CASF-2007 | ECIF::LD | 0.815 | 1.472 |
| CASF-2007 | Multi-shelled ECIF | **0.824** | **1.44** |
| CASF-2007 | Multi-shelled ECIF::LD | **0.828** | 1.423 |
| CASF-2007 | PDECIF | 0.816 | 1.455 |
| CASF-2007 | PDECIF::LD | 0.827 | **1.418** |
| CASF-2013 | ECIF | 0.781 | 1.464 |
| CASF-2013 | ECIF::LD | 0.801 | 1.419 |
| CASF-2013 | Multi-shelled ECIF | **0.803** | **1.442** |
| CASF-2013 | Multi-shelled ECIF::LD | 0.81 | 1.414 |
| CASF-2013 | PDECIF | 0.792 | 1.449 |
| CASF-2013 | PDECIF::LD | **0.811** | **1.405** |
| CASF-2016 | ECIF | 0.815 | 1.32 |
| CASF-2016 | ECIF::LD | 0.844 | 1.245 |
| CASF-2016 | Multi-shelled ECIF | **0.843** | 1.28 |
| CASF-2016 | Multi-shelled ECIF::LD | **0.851** | **1.252** |
| CASF-2016 | PDECIF | 0.833 | **1.277** |
| CASF-2016 | PDECIF::LD | 0.843 | **1.252** |
| CASF-2019 | ECIF | 0.832 | 1.284 |
| CASF-2019 | ECIF::LD | 0.856 | 1.211 |
| CASF-2019 | Multi-shelled ECIF | **0.868** | **1.199** |
| CASF-2019 | Multi-shelled ECIF::LD | **0.875** | **1.171** |
| CASF-2019 | PDECIF | 0.854 | 1.235 |
| CASF-2019 | PDECIF::LD | 0.859 | 1.217 |

| 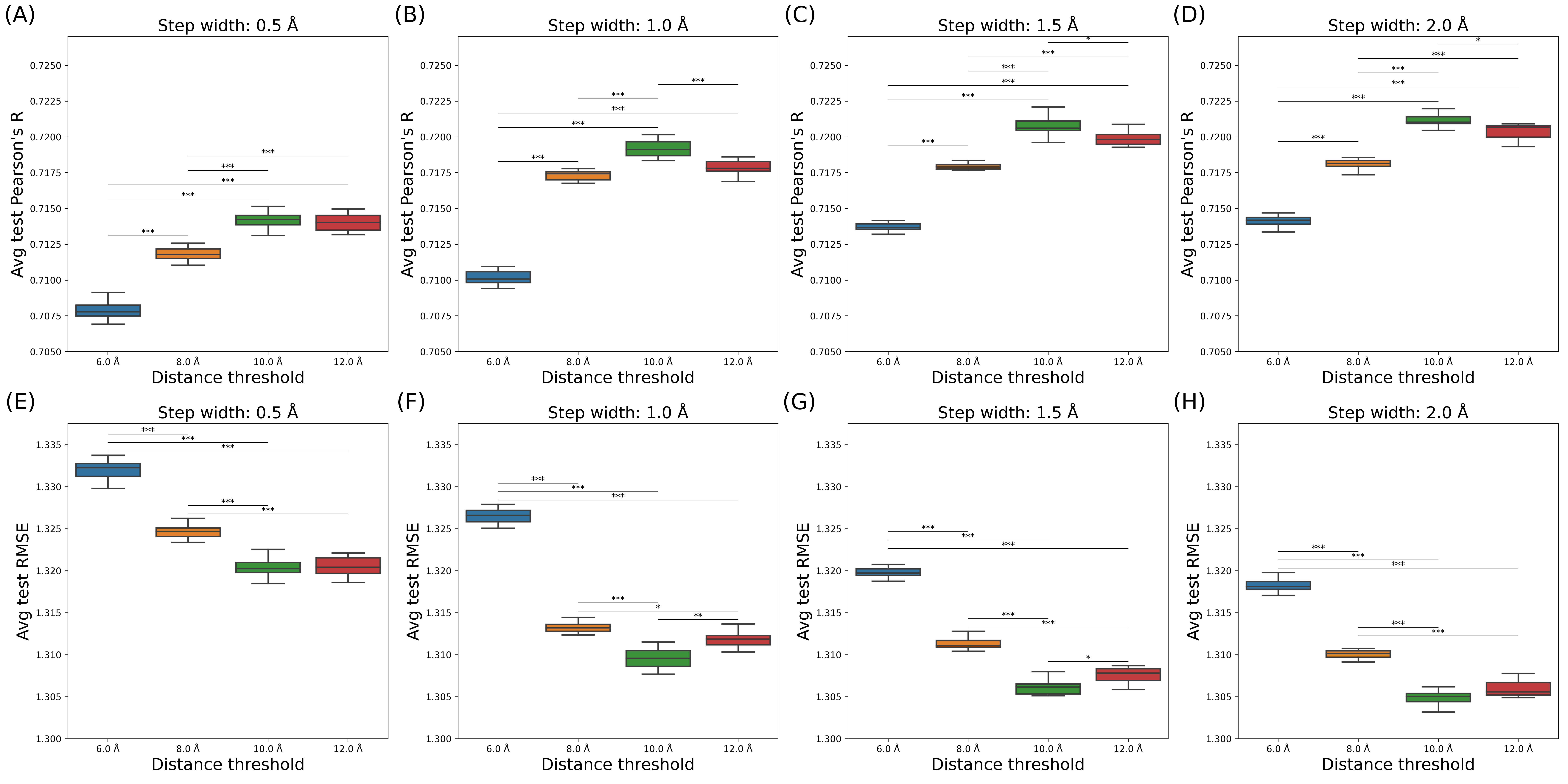 Supplementary Figure 1. Exploration of the optimal distance threshold of multi-shelled ECIF.  Each boxplot represents the results of 10 runs of 10-fold cross-validation over the training set. The upper four panels (A~D) show the average Pearson’s R of 10 runs, and the bottom four panels (E~H) show the average RMSE. The step width is indicated at the top of each plot. The horizontal axis represents the distance threshold. Combinations that are statistically significant by Bonferroni-corrected independent t-test are marked with *. (*** : p < 0.001, ** : 0.001 <= p < 0.01, * : 0.01 <= p < 0.05.) |
| --- |

| 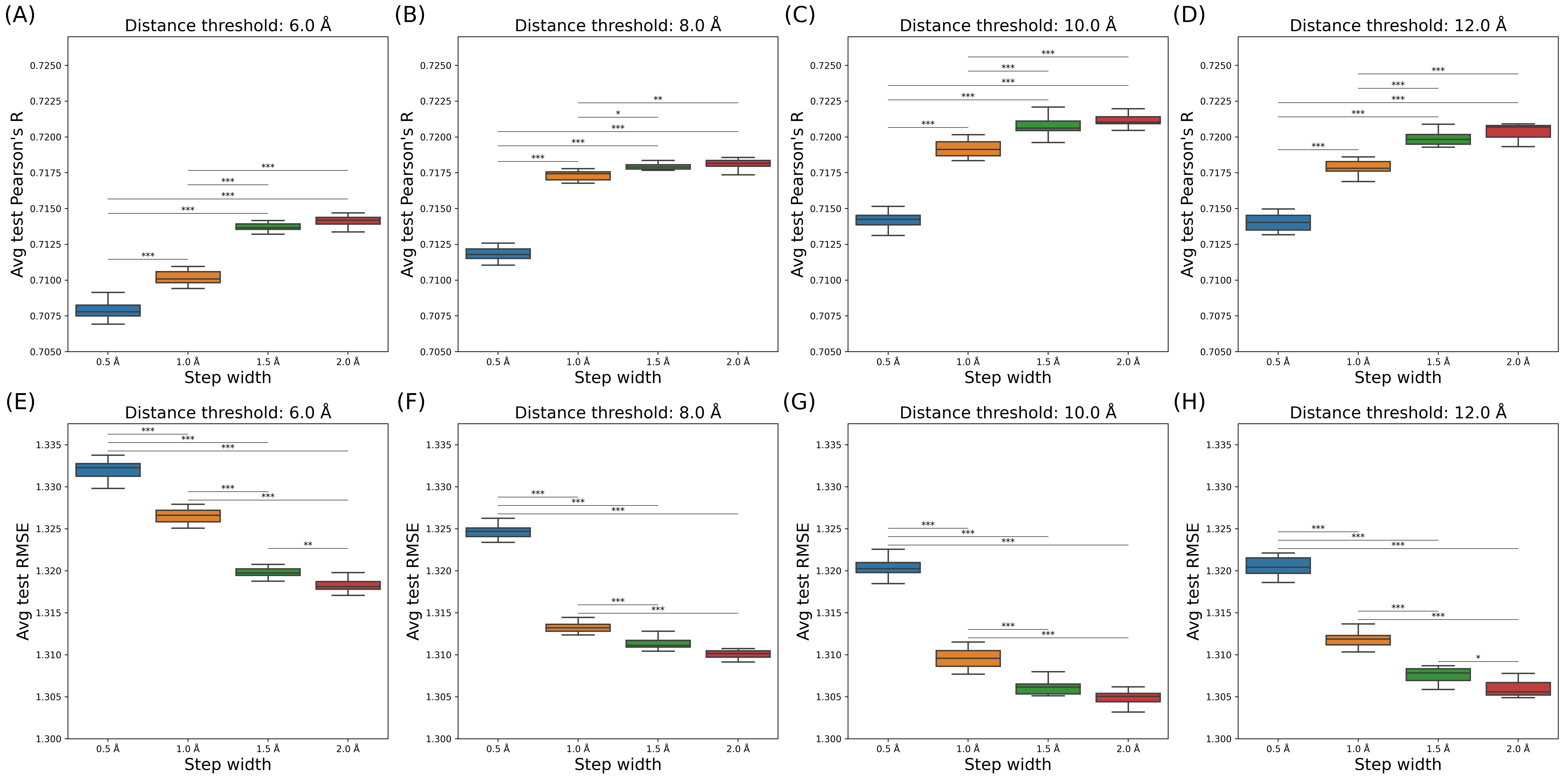 Supplementary Figure 2. Exploration of the optimal step width of multi-shelled ECIF.  Each boxplot represents the results of 10 runs of 10-fold cross-validation over the training set. The upper four panels (A~D) show the average Pearson’s R of 10 runs, and the bottom four panels (E~H) show the average RMSE. The distance threshold is indicated at the top of each plot. The horizontal axis represents the distance threshold. Combinations that are statistically significant by Bonferroni-corrected independent t-test are marked with *. (*** : p < 0.001, ** : 0.001 <= p < 0.01, * : 0.01 <= p < 0.05.) |
| --- |


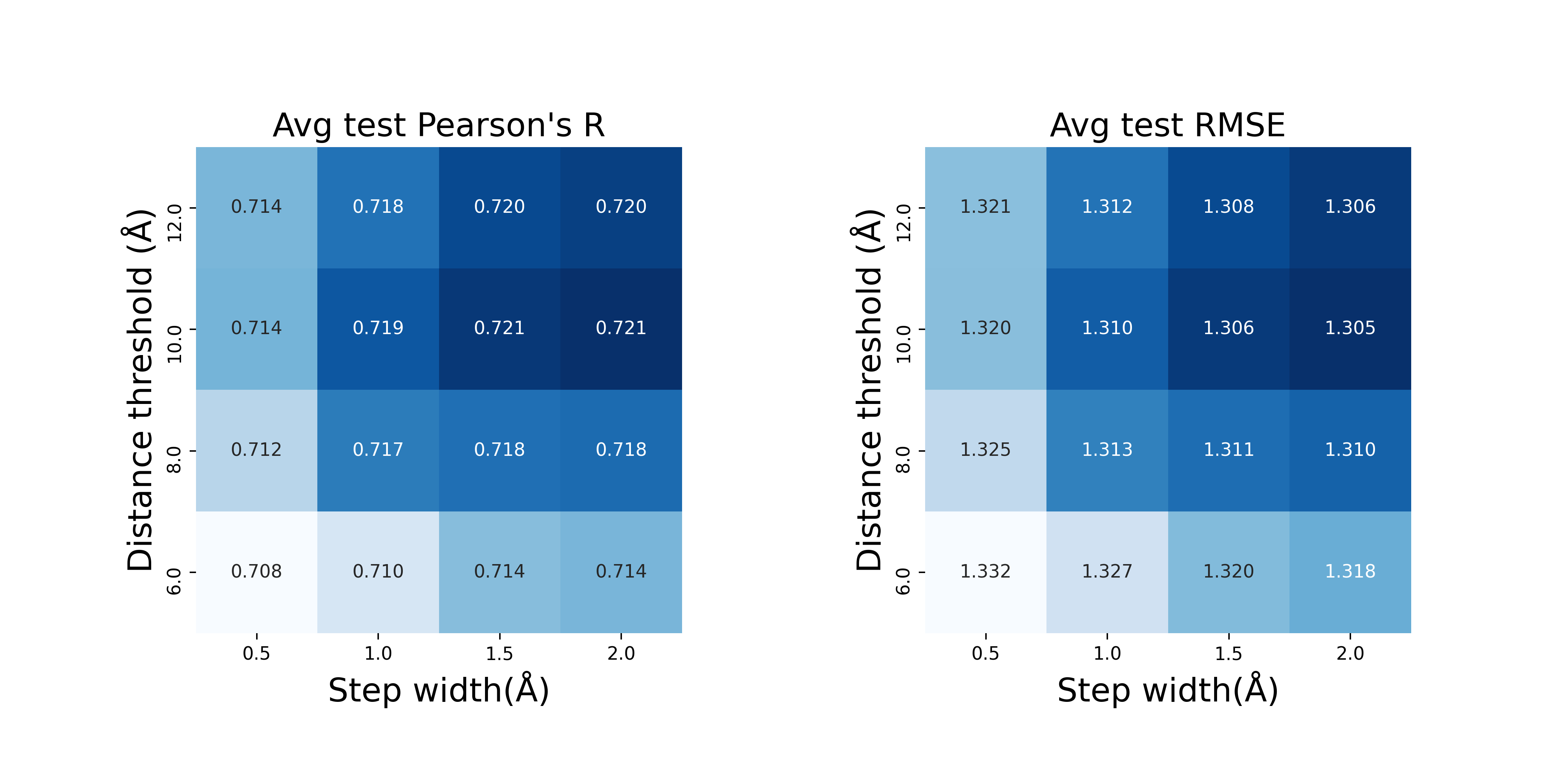


# Supplementary Figure 3. Result of the multi-shelled ECIF feature parameter exploration.

Each cell of the heat map represents the average Pearson’s R (left) and average RMSE (right) of 10 runs of 10 fold cross-validation under each condition. The horizontal axis of the heat map shows step width, and the vertical axis shows the distance threshold.


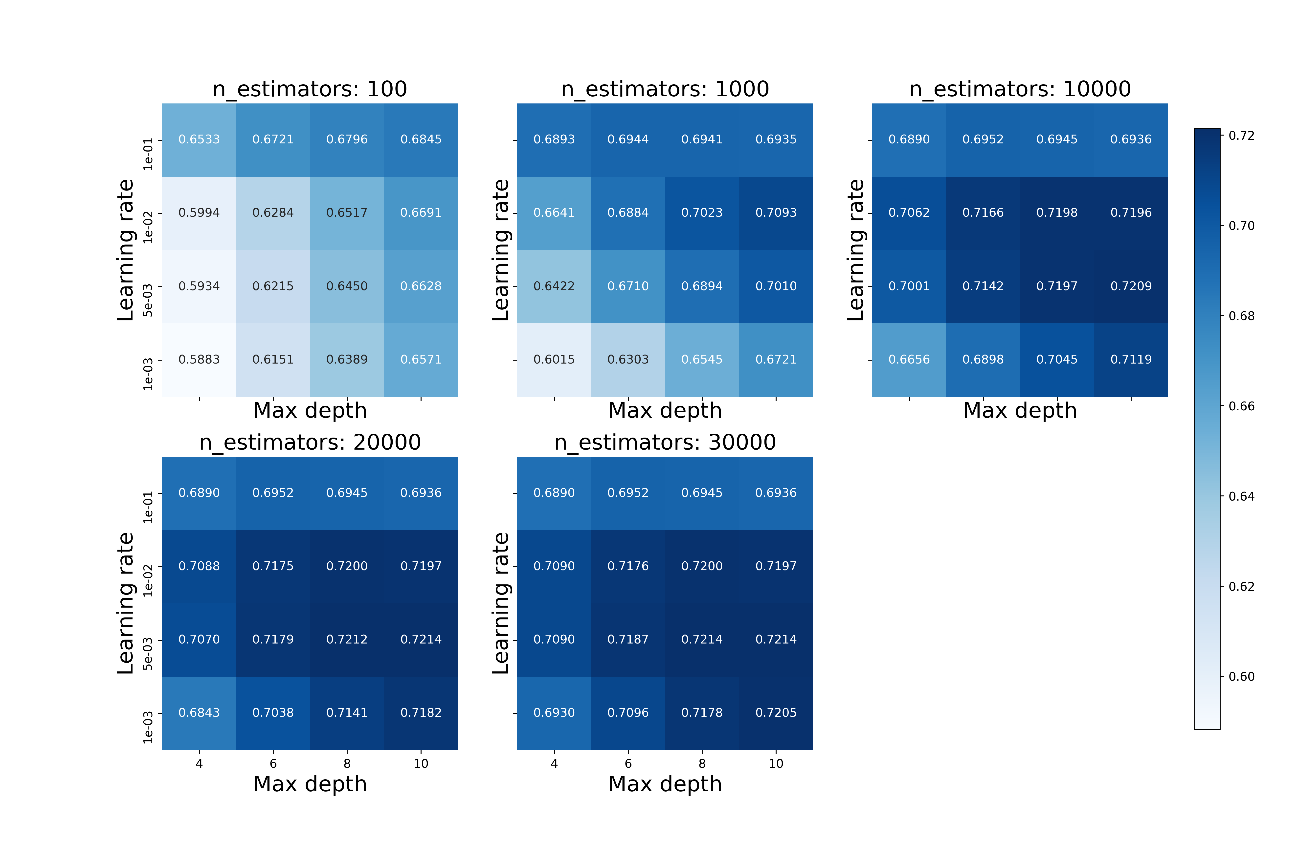

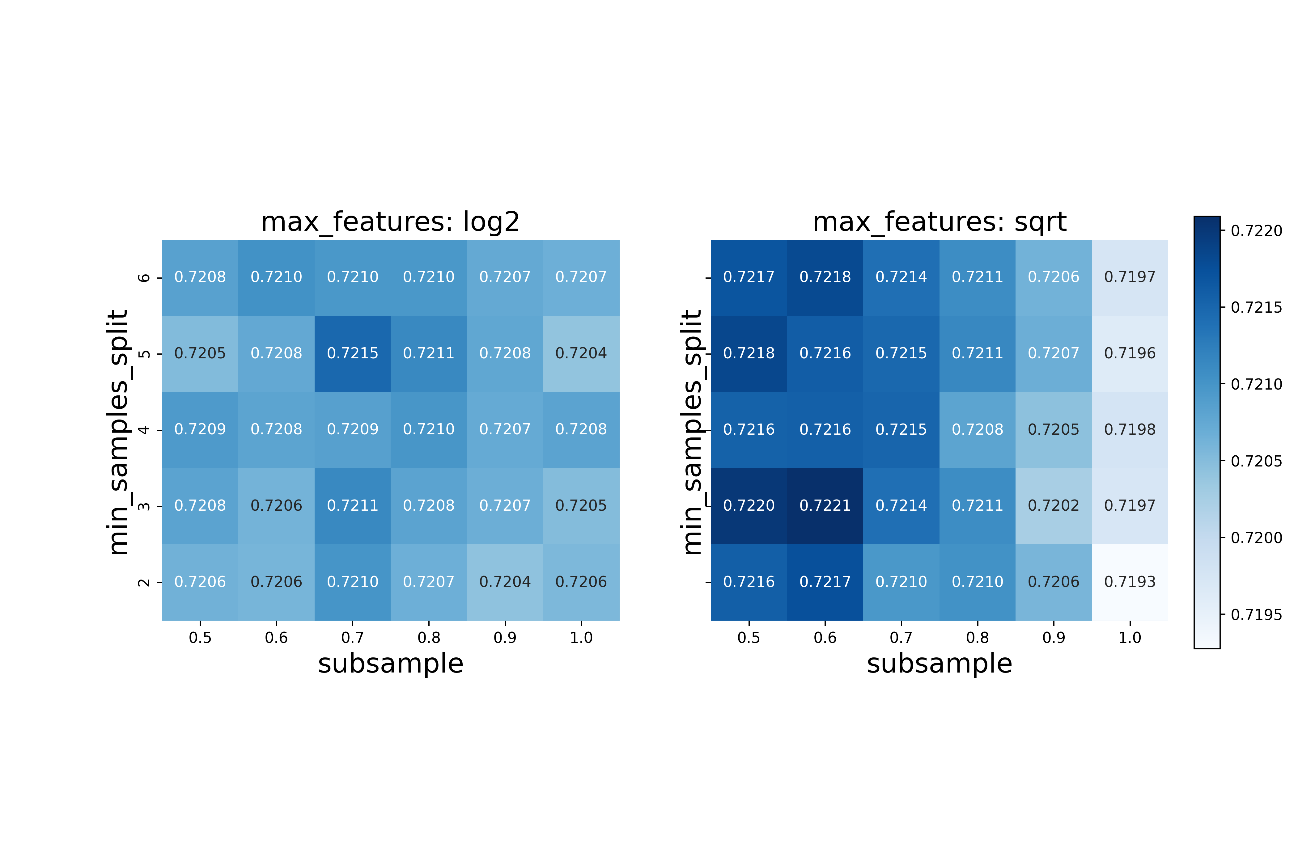


# Supplementary Figure 4. Result of the GBT parameters optimization of the multi-shelled ECIF.

Each cell in the heat map represents the average Pearson’s R of the 10 runs of 10 fold cross-validation trained under each condition. The horizontal axis of the heat map shows step width, and the vertical axis shows the distance threshold. The upper five panels show the optimization results for n_estimators, learning_rate, and max_depth, while the lower two panels show the optimization results for min_sample_split, max_features, and subsample.

| 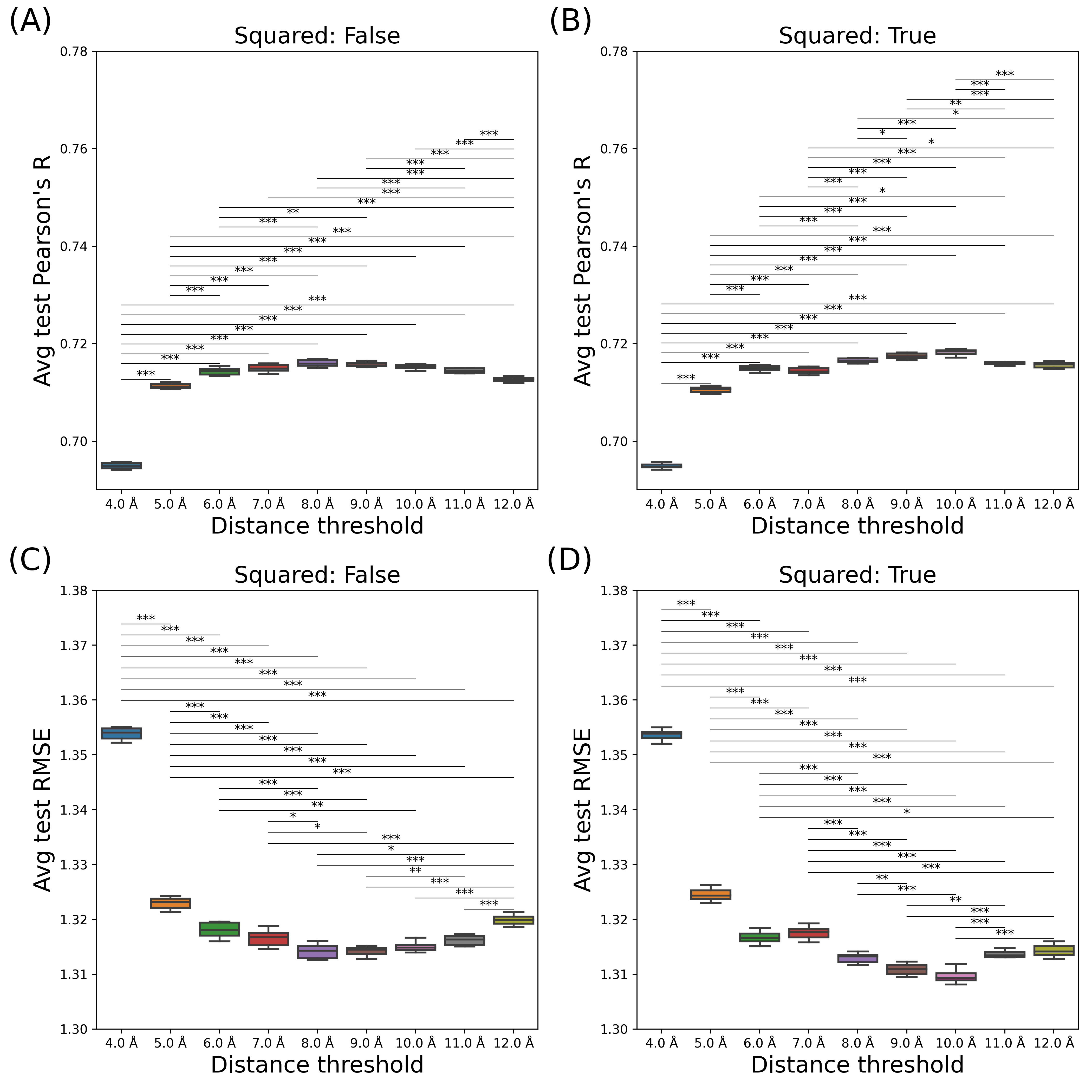 Supplementary Figure 5. Exploration of the optimal distance threshold of weighted ECIF.  Each boxplot represents the results of 10 runs of 10-fold cross-validation over the training set. The upper four panels (A, B) show the average Pearson’s R of 10 runs, and the bottom four panels (C, D) show the average RMSE. The “squared” parameter is indicated at the top of each plot. The horizontal axis represents the distance threshold. Combinations that are statistically significant by Bonferroni-corrected independent t-test are marked with *. (*** : p < 0.001, ** : 0.001 <= p < 0.01, * : 0.01 <= p < 0.05.) |
| --- |

|  Supplementary Figure 6. Exploration of the optimal “squared” parameter of weighted ECIF.  Each boxplot represents the results of 10 runs of 10-fold cross-validation over the training set. The upper four panels (A~I) show the average Pearson’s R of 10 runs, and the bottom four panels (J~R) show the average RMSE. The distance threshold is indicated at the top of each plot. The horizontal axis represents the “squared” parameter. Combinations that are statistically significant by Bonferroni-corrected independent t-test are marked with *. (*** : p < 0.001, ** : 0.001 <= p < 0.01, * : 0.01 <= p < 0.05.) |
| --- |


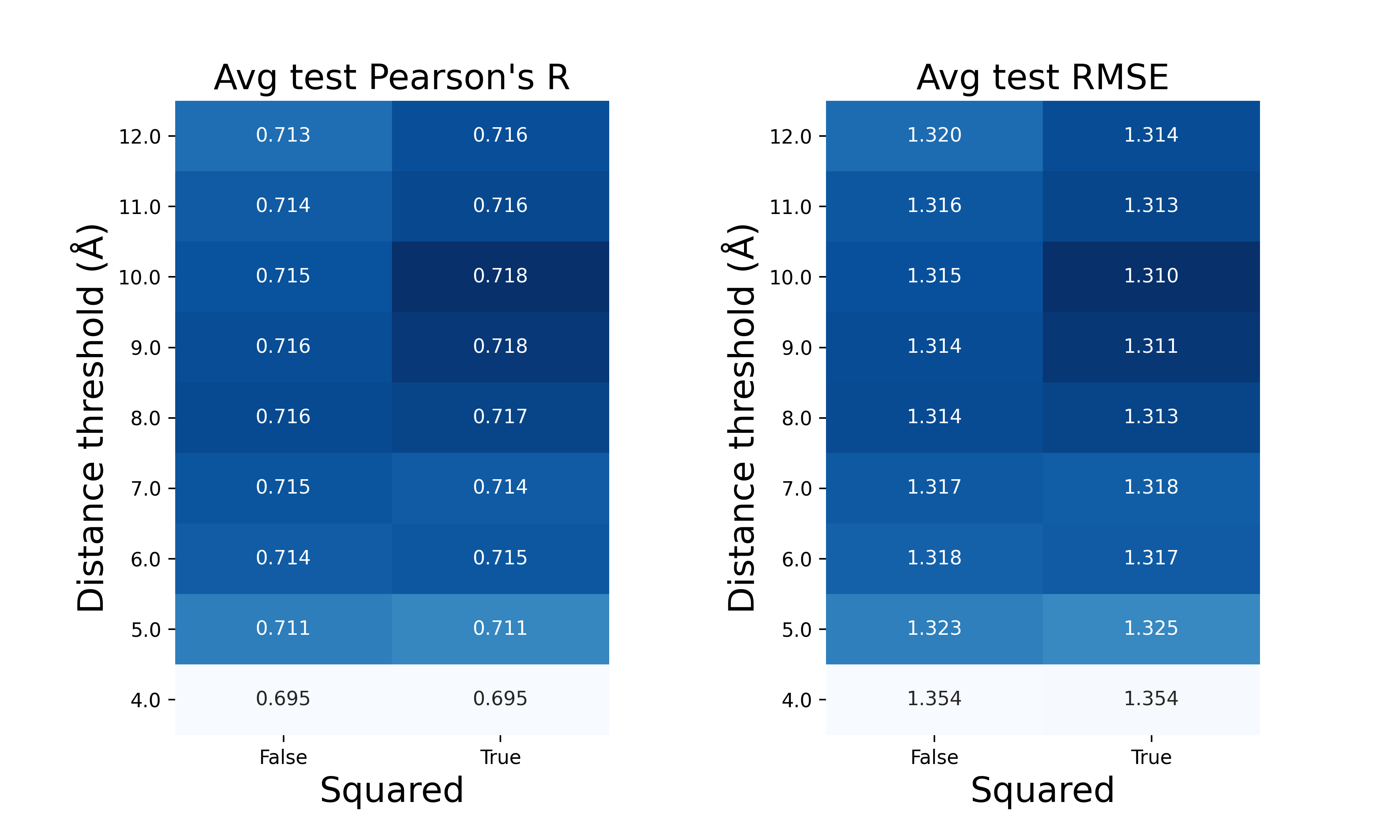


# Supplementary Figure 7. Result of the weighted ECIF feature parameter exploration.

Each cell in the heatmap represents the average Pearson’s R (left) and average RMSE (right) of 10 runs of 10 fold cross-validation under each condition. The horizontal axis of the heat map shows the “squared” parameter, and the vertical axis shows the distance threshold.


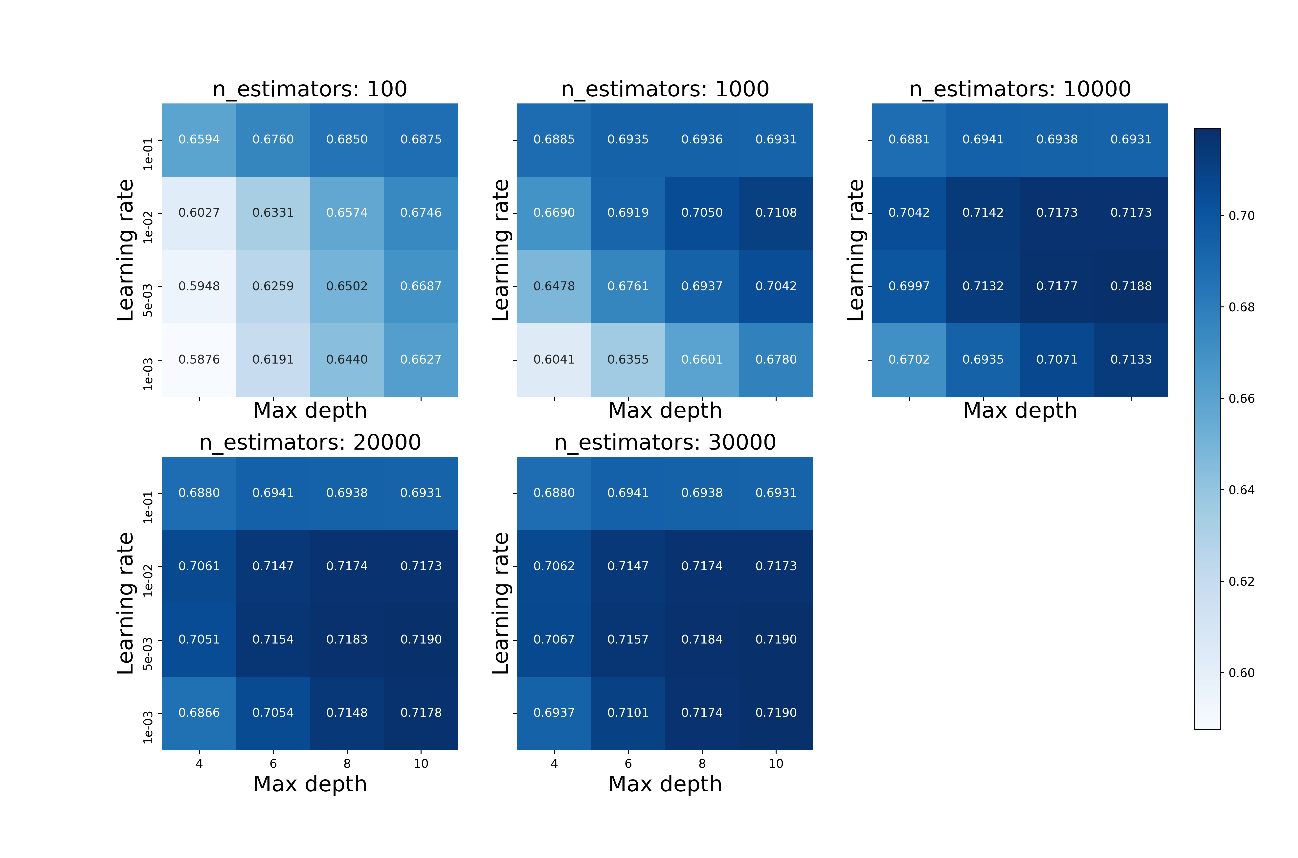

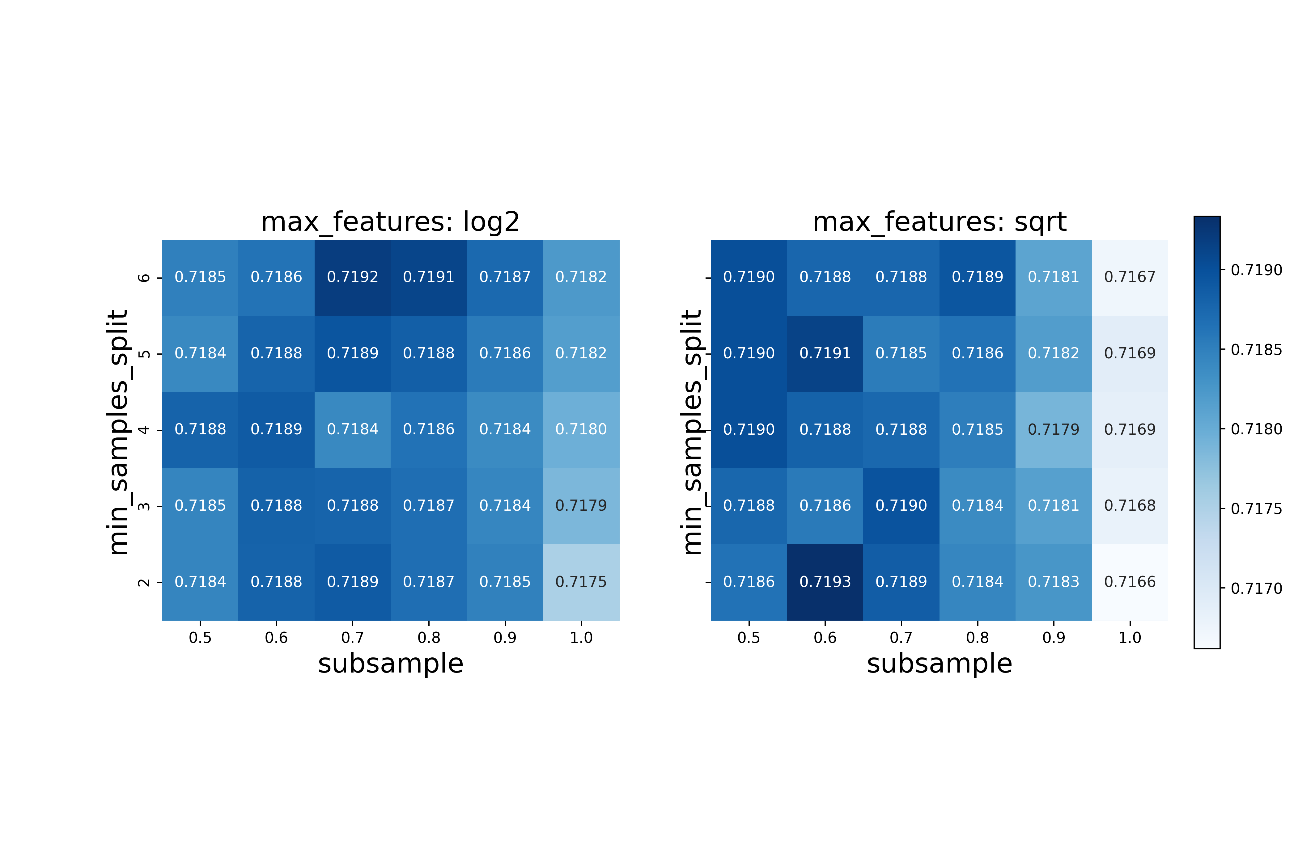


# Supplementary Figure 8. Result of the GBT parameters optimization of the weighted ECIF.

Each cell in the heat map represents the average Pearson’s R of the 10 runs of 10 fold cross-validation trained under each condition. The horizontal axis of the heat map shows step width, and the vertical axis shows the distance threshold. The upper five panels show the optimization results for n_estimators, learning_rate, and max_depth, while the lower two panels show the optimization results for min_sample_split, max_features, and subsample.


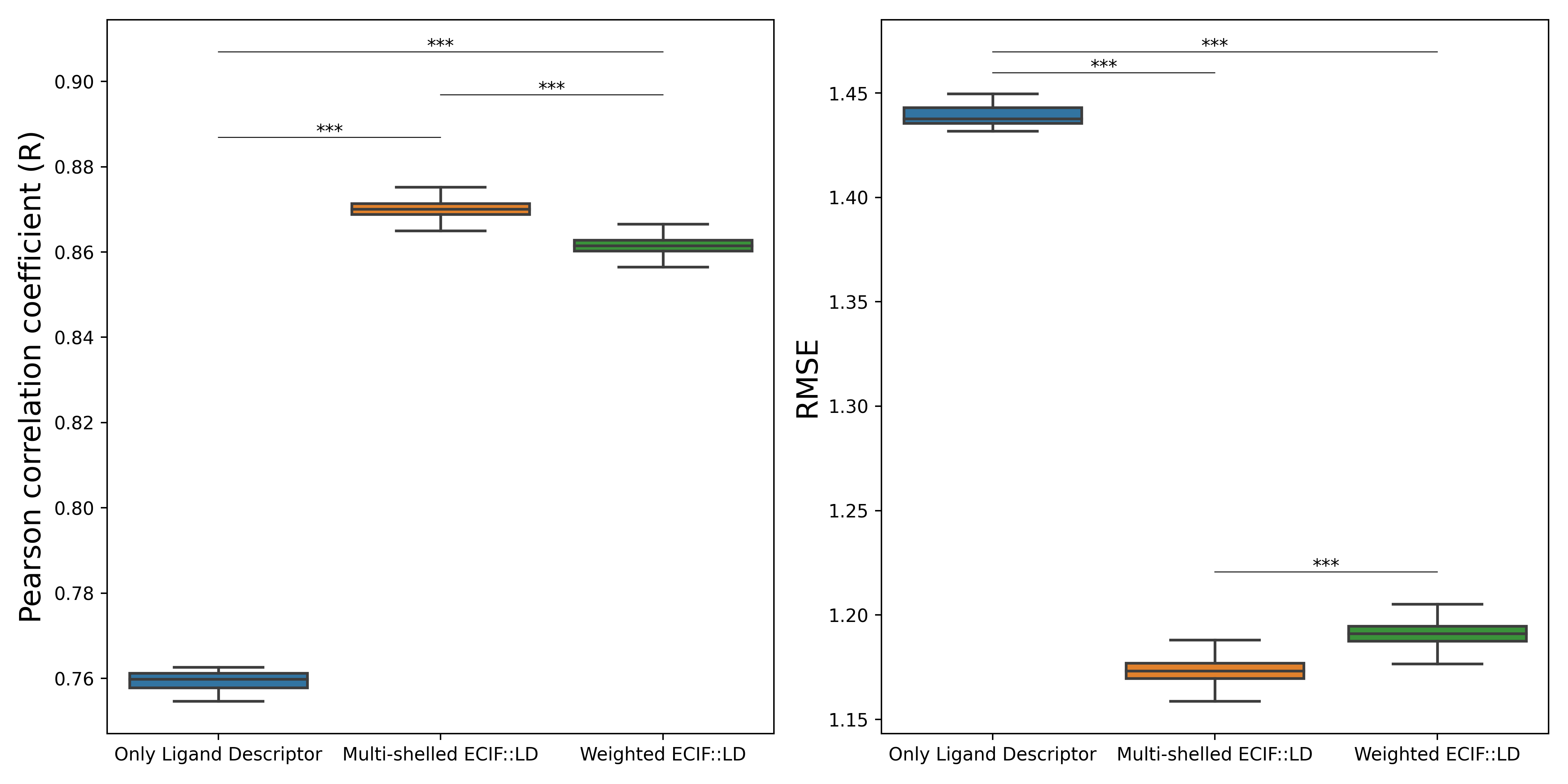


# Supplementary Figure 9. Comparison with the models trained by only ligand descriptors.

Each boxplot represents the average Pearson’s R (left) and average RMSE (right) of the models for each method trained with different random seeds and evaluated on the CASF 2016 "core set". The boxplot of “Only Ligand Descriptor” shows the average of 10 models and the boxplots of Multi-shelled ECIF and Weighted ECIF show the average of 5000 models. The vertical axis shows the distance range of the features. Combinations that are statistically significant by Bonferroni-corrected independent t-test are marked with *. (*** : p < 0.001, ** : 0.001 <= p < 0.01, * : 0.01 <= p < 0.05.)


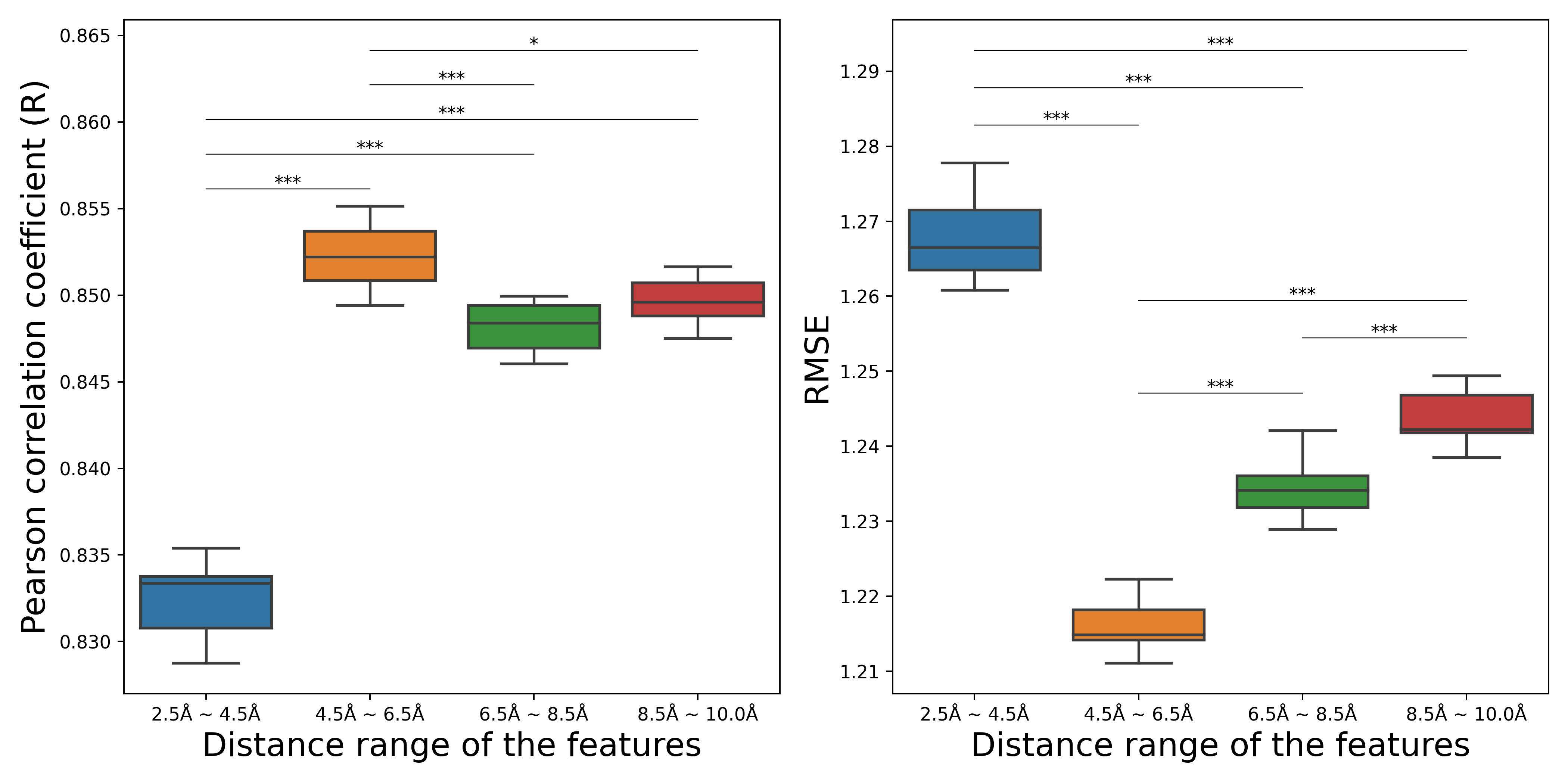


# Supplementary Figure 10. Comparison with the models trained by the features at specific distance range.

Each boxplot represents the average Pearson’s R (left) and average RMSE (right) of 10 models trained under each condition with different random seeds and evaluated with CASF2016 ‘core set’. The vertical axis shows the distance range of the features. Combinations that are statistically significant by Bonferroni-corrected independent t-test are marked with *. (*** : p < 0.001, ** : 0.001 <= p < 0.01, * : 0.01 <= p < 0.05.)
